# Transcription-dependent domain-scale three-dimensional genome organization in the dinoflagellate *Breviolum minutum*

**DOI:** 10.1038/s41588-021-00848-5

**Published:** 2021-04-29

**Authors:** Georgi K. Marinov, Alexandro E. Trevino, Tingting Xiang, Anshul Kundaje, Arthur R. Grossman, William J. Greenleaf

**Affiliations:** 1 Department of Genetics, Stanford University, Stanford, CA USA; 2 Center for Personal Dynamic Regulomes, Stanford University, Stanford, CA USA; 3 Department of Bioengineering, Stanford University, Stanford, CA USA; 4 Department of Plant Biology, Carnegie Institution for Science, Stanford, CA USA; 5 Department of Biological Sciences, University of North Carolina at Charlotte, Charlotte, NC USA; 6 Department of Computer Science, Stanford University, Stanford, CA USA; 7 Department of Applied Physics, Stanford University, Stanford, CA USA; 8 Chan Zuckerberg Biohub, San Francisco, CA USA

**Keywords:** Genomics, Epigenomics

## Abstract

Dinoflagellate chromosomes represent a unique evolutionary experiment, as they exist in a permanently condensed, liquid crystalline state; are not packaged by histones; and contain genes organized into tandem gene arrays, with minimal transcriptional regulation. We analyze the three-dimensional genome of *Breviolum minutum*, and find large topological domains (dinoflagellate topologically associating domains, which we term ‘dinoTADs’) without chromatin loops, which are demarcated by convergent gene array boundaries. Transcriptional inhibition disrupts dinoTADs, implicating transcription-induced supercoiling as the primary topological force in dinoflagellates.

## Abstract (web summary)

Analysis of the three-dimensional architecture of the dinoflagellate *Breviolum minutum* genome identifies large topological domains (dinoTADs) demarcated by convergent gene array boundaries. Transcriptional inhibition disrupts dinoTADs.

## Main

The three-dimensional (3D) genome architecture of cells has functional consequences for gene regulation, organismal development, DNA replication and mutational processes. Topologically associating domains (TADs) and compartments on the submegabase scale are conserved architectural features of eukaryote genomes, defined by increased intradomain contact frequencies and interdomain contact insulation^1^. Mechanisms known to drive the folding of such domains include constraints on cohesin-mediated loop extrusion—imposed in part by CTCF in vertebrates—and self-associations between similar chromatin states^2^. Other mechanisms, including insulation of domains by polymerases or specific boundary proteins, have also been proposed to play roles in genome architecture^3^. However, the extent to which genome function may influence genome folding, for example, through transcriptional activity, is poorly understood. There has also been little exploration of 3D organization across eukaryotes, even though major deviations from conventional norms are known to exist, presenting natural experiments that may reveal deeper underlying organizational principles masked in other lineages.

Dinoflagellates are the most radical such departure. They are a diverse, widespread clade playing major roles in aquatic ecosystems, for example, as symbionts of corals, providing the metabolic basis for reef ecosystems. Dinoflagellates possess numerous highly divergent molecular features^4^, including, uniquely among eukaryotes, the loss of nucleosomal packaging of chromatin. Histones are extremely conserved across eukaryotes, were present in their current form already in the last eukaryotic common ancestor^5^, and they and their post-translational modifications are pivotal to all biochemical processes involving chromatin.

Dinoflagellates are the sole known exception. Their chromosomes exist in a liquid crystalline state and are permanently condensed throughout the cell cycle, and, although highly divergent histone genes are retained in their genomes^6^, a combination of virus-derived nucleoproteins and bacterial-derived histone-like proteins have taken over as the main packaging components^7^. Dinoflagellate genomes are often huge (up to ≥200 gigabases), genes are organized into tandem gene arrays, individual messenger RNAs are generated through *trans*-splicing and transcriptional regulation is largely absent^7^. These fascinating features simultaneously pose intriguing questions regarding the adaptation of transcriptional and regulatory mechanisms to the absence of nucleosomes, and provide a unique opportunity to explore the biophysical forces underlying genomic organization in the context of a large eukaryotic genome nearly devoid of nucleosomes.

**Fig. 1:**
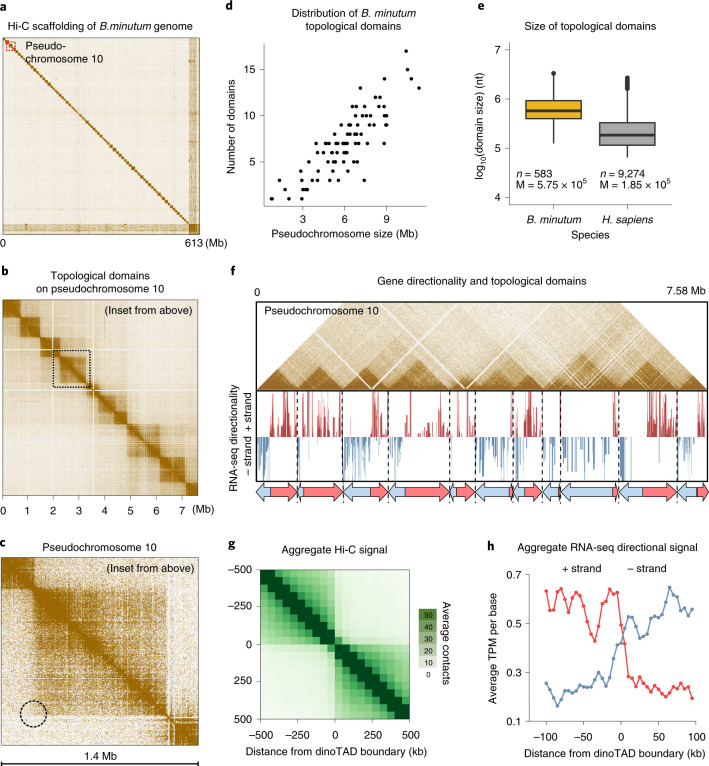
*B. minutum* genome is physically partitioned into dinoTADs defined by tandem gene arrays. **a**, Hi-C scaffolding of the *B. minutum* draft genome assembly. **b**, Inset from **a**. KR-normalized 5-kb resolution Hi-C map for pseudochromosome 10. **c**, Inset from **b**. Hi-C loops and stripes are not observed in dinoTADs (dotted circle notes where a loop would be). **d**, Scaling of chromosome size with dinoTAD number. **e**, Comparison of human and *B. minutum* topological domain sizes. Box plots show the 25th, 50th and 75th percentiles; whiskers show the 5–95% intervals; *n* = 583 for dinoTADs; *n* = 9,274 for human TADs. **f**, Hi-C map (5-kb resolution) for pseudochromosome 10 together with forward- and reverse-strand transcript levels and gene arrays. **g**, Average Hi-C contacts across dinoTAD boundaries (dinoTADs were called at a 50-kb resolution using HiCExplorer^22^; see the Methods for details). **h**, Average forward- and reverse-strand RNA-seq levels across dinoTAD boundaries. *H. sapiens*, *Homo sapiens*; KR, KR normalization; M, mean (bp); nt, nucleotides; TPM, transcripts per million.

To explore these questions, we applied chromosome conformation capture using Hi-C on the coral symbiont *Breviolum minutum*. We generated multiple libraries under standard growth conditions and for cells grown at elevated temperature, obtaining ∼150–220 million Hi-C contacts for each (Supplementary Table 1). We pooled these libraries to generate a chromosome-level scaffolding of the previously fragmented *B. minutum* assembly^8^. We identified 91 major pseudochromosomes (≥500 kilobases (kb)), encompassing ∼94% of the total sequence (Fig. 1a,b and Supplementary Fig. 1a), the longest being ∼11 megabases (Mb) in size, with a median length of 6.7 Mb (Supplementary Fig. 1a). At 1-Mb resolution, they exhibit a bipartite (occasionally tripartite) structure (Supplementary Fig. 2).

Additional untreated libraries (Supplementary Table 1) were combined to generate an even-higher-resolution map (∼1.05 billion contacts), which was used to examine fine-scale features of topological organization. High-resolution (5 kb) maps revealed well-defined (comparably so with those observed in mammals) topological domains, ≤200 kb to ≥2 Mb in size (Fig. 1b,e and Supplementary Figs. 3–12). In mammals, most TAD boundaries are demarcated by CTCF sites blocking loop extrusion, reflected in Hi-C maps by chromatin loops and ‘stripes’. We observed no loop or stripe features in *B. minutum* (Fig. 1c), suggesting a different mechanism for the formation of dinoflagellate TADs, which we term ‘dinoTADs’. Omitting the denaturation step in the Hi-C protocol, which should better preserve protein–protein contacts, strongly accentuated dinoTADs, but still did not reveal signs of loops or loop extrusion domains (Supplementary Fig. 14). Detected dinoTAD number correlated with chromosome size (Fig. 1d), and observed dinoTADs were considerably larger than mammalian TADs (Fig. 1e).

We next compared Hi-C maps with available annotation features. Remarkably, we found that each dinoTAD corresponded to a pair of divergent gene arrays (Fig. 1f), and dinoTAD boundaries coincided with convergence between gene arrays (Fig. 1g,h).

Numerous models for dinoflagellate chromosome organization have been suggested since the 1960s, primarily based on electron microscopy. These include proposals that chromosomes are organized as ‘toroidal chromonemas’^9^, as ‘stacks of disks’^10^, as ‘cored pineapples’^11^ or around ‘central core fibers’^12^. Most of these models imply specific topological constraints maintaining the proposed shapes and are not directly reconcilable with our Hi-C observations.

Instead, the correspondence between dinoTADs and gene arrays suggested a role for transcription in their formation. Although TADs form independently of transcription in animal cells, transcription-induced self-interacting domains have been previously demonstrated in bacteria^13^, and similar mechanisms have been proposed to explain some topological features in fission yeast^14^. We also note that a handful of models of dinoflagellate chromosome structure have suggested the presence of coil/plectoneme-like features^15,16^, but without relating them to gene arrays and transcription. This model is also supported by the observation that frequently each dinoTAD can be divided into more diffuse ‘sub-dinoTADs’ corresponding to the two individual gene arrays in a pair (Fig. 1c and Supplementary Figs. 3–12), which could be the result of torsion generated in each direction of transcription.

The model makes a clear prediction—inhibition of transcription should result in dinoTADs decompaction. To test this relationship, we first compared Hi-C maps for cells grown at 34 °C versus 27 °C, as heat stress could result in general transcription reduction^17^. We observed mild decompaction of dinoTADs at 34 °C, although domains remained intact (Supplementary Figs. 19–21).

**Fig. 2:**
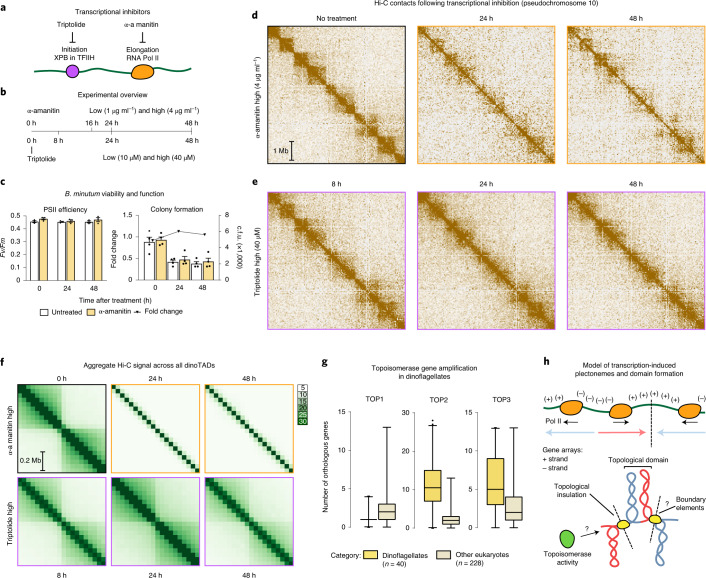
Decompaction of dinoTADs upon application of transcriptional inhibitors and the transcription-induced supercoiling model for their formation. Shown is pseudochromosome 10 as in Fig. 1. **a**, Outline of transcription inhibition experiments. **b**, Outline of transcription inhibition time course. **c**, Comparison of cell function, measured by PSII photosynthetic efficiency (left, *n* = 3 biological replicates for each condition), and cell viability, measured by colony formation (right; *n* = 4 biological replicates for each condition), between α-amanitin-treated and untreated cells. Treatment with α-amanitin does not affect PSII activity (Student *t*-test, *P* = 0.75304979 for 0 h, *P* = 0.442327976 for 24 h, *P* = 0.23349803 for 48 h). Error bars show mean ± s.d. **d**, KR-normalized Hi-C maps (50-kb resolution) show marked loss of dinoTADs after α-amanitin treatment. **e**, Hi-C maps show reduction of insulation at dinoTAD boundaries after triptolide treatment. **f**, Metaplots of Hi-C signal around domain boundaries (50-kb resolution). **g**, Amplification of *TOP2* and *TOP3* topoisomerases in dinoflagellates (based on MMETSP^23^ transcriptome assemblies). Box plots show the 25th, 50th and 75th percentiles; whiskers show the 5–95% intervals. The dinoflagellate (*n* = 41) and nondinoflagellate (*n* = 243) species shown are the ones from Supplementary Table 2. **h**, Transcription-induced supercoiling as driver of dinoflagellate chromatin folding. Transcribing polymerases introduce negative/positive DNA supercoiling behind/ahead of the transcription machinery. Interactions within supercoiled domains could explain the physical association of divergently oriented arrays. Topological insulation could be driven by supercoiling-related effects, or by specific boundary elements. c.f.u., colony-forming units.

We next carried out chemical transcription inhibition experiments. Since transcription inhibition conditions for *B. minutum* are not well established, we chose two inhibitors—triptolide and α-amanitin—with distinct mechanisms of action, and assayed multiple time points and doses (Fig. 2a,b). Amanitin directly inhibits RNA polymerase II and is slow acting, while triptolide quickly blocks initiation by targeting the TFIIH XPB subunit^18^. While dinoflagellate RNA polymerase II has been reported to be sensitive to α-amanitin, it is possible that the sensitivity is somewhat partial^19^; in addition, the *B. minutum* XPB homolog is highly divergent^8^, and thus a moderate inhibition effect is not unexpected. We therefore carried out several experiments to directly estimate the extent of transcription inhibition. Direct metabolic labeling approaches^20^ were unsuccessful, as it appears that Symbiodiniaceae cells are impermeable to nucleotide and nucleoside analogs such as 4-thiouridine and 4-thiouracil. We were, however, able to qualitatively assess inhibition using the proxy of nascent RNA, as measured by the proportion of unspliced reads in poly(A)^+^ RNA-sequencing (RNA-seq) datasets (Supplementary Fig. 30). We observed more than 50% reduction in unspliced reads in both α-amanitin and triptolide cells after 48 h, suggesting that transcription was indeed inhibited. We also did not observe large-scale changes in the levels of individual transcripts (Supplementary Fig. 31). Finally, even at high doses, α-amanitin treatment did not detectably affect photosynthetic efficiency or cell viability relative to controls (Fig. 2c), excluding cell death as a confounding factor.

Strikingly, α-amanitin treatment resulted in a dose-dependent, progressive dinoTAD decompaction (Fig. 2d,f and Supplementary Figs. 22–25). These effects were observed in both technical and biological replicates (Supplementary Figs. 22–25). We also observed clear dose-dependent blurring of dinoTAD boundaries after triptolide treatment, although broad dinoTAD-like structures remained visible to a greater extent than in α-amanitin-treated cells (Fig. 2e,f and Supplementary Figs. 26–29).

These experiments support a transcription-induced supercoiling model for dinoTAD formation. Torque generated by active polymerases produces positive/negative supercoiling ahead of/behind the transcription bubble. This can alter the twist of the double helix or induce superhelical writhe, which in turn can be accommodated through nucleosome remodeling, local alterations in DNA secondary structure, or formation of structures such as plectonemes^21^, from which we would expect strong Hi-C signals comprising our observed domains.

Although other topological constraints might also be involved, supercoiling-induced plectoneme formation over gene arrays is an intuitive mechanistic explanation for the presence of dinoTADs. An examination of dinoflagellate gene repertoires also corroborates this model, revealing a striking, dinoflagellate-specific expansion of topoisomerase II- and topoisomerase III-like genes (Fig. 1d, Supplementary Fig. 18 and Supplementary Table 2), further suggestive of contending with increased levels of writhed forms of helical twist.

Comparison with self-interacting domains in bacteria or *Schizosaccharomyces pombe* shows much stronger topological insulation for dinoTADs (Supplementary Figs. 15 and 16). Remarkably, no TAD domains are observed in kinetoplastids, the other lineage with long gene arrays and no transcriptional regulation (Supplementary Fig. 17).

These differences can be rationalized by the unusual dinoflagellate properties. First, neither bacteria nor yeast possess comparably long gene arrays and transcription in those species is highly nonuniform; less transcription-induced torsional stress is therefore expected. Nucleosome loss is the second, and most salient, difference. Single mammalian genes as long as dinoTADs are quite common, yet contact domains aligning with gene boundaries is not apparent in mammalian Hi-C maps, nor is it seen in kinetoplastids, which have gene arrays but also conventional chromatin. We therefore hypothesize that plectonemic structures form due to transcription-induced supercoiling in the nucleosome-depleted genomes of dinoflagellates, while, in other eukaryotes, a combination of the wrapping of DNA around nucleosomes, interactions between nucleosomes and accumulation of DNA twist prevents their formation (Fig. 2h).

These results generate a number of open questions. How exactly are boundaries between dinoTADs formed mechanistically? Specific boundary elements of markedly different chromatin states could exist; alternatively, these boundaries may self-organize purely through torsion-related mechanisms. The roles that dinoflagellates’ divergent histone genes play are also not clear. Finally, the relationship between Hi-C features and the ‘toroidal chromonemas’^9^ observed by electron microscopy remains unknown. Answers to these questions, together with the dissection of specific roles of different topoisomerase classes, will help to fully elucidate the interplay between packaging proteins, transcription-induced torsional stress and genome folding in dinoflagellates.

These observations also identify transcription-induced torsional stress as a key direction of future studies in eukaryotes generally. The strength of dinoTADs underlines the potency of this fundamental biological process for generating topological structure. The precise manner by which torsion is accommodated as twist and writhe, as well as its consequences for regulatory protein occupancy, transcriptional activity and other chromatin processes, such as the behavior of ATP-dependent chromatin remodelers, are exciting questions remaining to be unraveled.

## Methods

Except where otherwise stated, computational analyses were carried out using custom-written Python scripts.

### *B. minutum* cell culture

The clonal axenic *B. minutum* strain SSB01 was used in all experiments. Stock cultures were grown as previously described^24,25^ in Daigo’s IMK medium for marine microalgae (Wako Pure Chemicals) supplemented with casein hydrolysate (IMK + Cas) at 27 °C at a light intensity of 10 µmol photons m^−2^ s^−1^ from Philips ALTO II 25-W bulbs on a 12-h-light/12-h-dark cycle. The medium was prepared in artificial seawater.

### Transcription inhibition experiments

For α-amanitin treatment, *B. minutum* cells at a density of ∼1 × 10^6^ cells per ml were treated with α-amanitin (Sigma-Aldrich, catalog no. A2263) at concentrations of 1 µg ml^−1^ (‘normal’ dose) and 4 µg ml^−1^ (‘high’ dose).

Samples were collected at 0, 24 and 48 h after treatment.

For triptolide treatment, *B. minutum* cells at a density of ∼1 × 10^6^ cells per ml were treated with triptolide (Sigma-Aldrich, catalog no. T3652) at concentrations of 10 µM (‘normal’ dose) and 40 µM (‘high’) dose. Samples were collected at 0, 8, 24 and 48 h after treatment.

### Cell viability measurements

#### Photosynthetic activity

Maximum quantum yield of photosystem II, *Fv/Fm* = (*Fm* − *F*0)*/Fm*, where *Fm* is maximum fluorescence, *Fv* is variable fluorescence, and *F*0 is minimum fluorescence, was used to indicate photosynthetic function. *B. minutum* cultures (approximately 10^6^ cells per ml) were collected and dark adapted for 5 min, and *Fv/Fm* was determined using a Dual Pam-100 fluorometer (Heinz Walz).

#### Colony formation assay

Fresh SSB01 cells were sampled at 0, 24 and 48 h after the treatment with transcription inhibitor α-amanitin. For each condition, cell suspensions were diluted 1:5 and 1:10 before plating 1 µl of each dilution on marine broth (BD) agar plates. Plates were incubated at 27 °C at a light intensity of 10 µmol photons m^2^ s^1^. Cell numbers on each plate were counted after 3 weeks.

### Hi-C experiments

The in situ Hi-C procedure used to map 3D genomic interactions in *B. minutum* was adapted from previous studies^2^ as follows:

*B. minutum* SSB01 cells were first crosslinked using 37% formaldehyde (Sigma) at a final concentration of 1% for 15 min at room temperature. Formaldehyde was then quenched using 2.5 M glycine at a final concentration of 0.25 M. Cells were subsequently centrifuged at 2,000*g* for 5 min, washed once in 1× PBS and stored at −80 °C.

Cell lysis was initiated by incubation with 250 µl of cold Hi-C lysis buffer (10 mM Tris-HCl pH 8.0, 10 mM NaCl, 0.2% Igepal CA630) on ice for 15 min, followed by centrifugation at 2,500*g* for 5 min, a wash with 500 µl of cold Hi-C lysis buffer and centrifugation at 2,500*g* for 5 min. The pellet was then resuspended in 50 µl of 0.5% SDS and incubated at 62 °C for 10 min (except for the ‘no-denaturation’ sample, for which the pellet was resuspended in 50 µl of H_2_O). SDS was quenched by adding 145 µl of H_2_O and 25 µl of 10% Triton X-100 and incubating at 37 °C for 15 min.

Restriction digestion was carried out by adding 25 µl of 10× NEBuffer 2 and 100 U of the MboI restriction enzyme (NEB, R0147) and incubating for ≥2 h at 37 °C in a Thermomixer at 900 r.p.m. The reaction was then incubated at 62 °C for 20 min to inactivate the restriction enzyme.

Fragment ends were filled in by adding 37.5 µl of 0.4 mM biotin-14-dATP (ThermoFisher Scientific, no. 19524-016); 1.5 µl each of 10 mM dCTP, dGTP and dTTP; and 8 µl of 5 U µl^−1^ DNA Polymerase I Large (Klenow) Fragment (NEB M0210). The reaction was then incubated at 37 °C in a Thermomixer at 900 r.p.m. for 45 min.

Fragment end ligation was carried out by adding 663 µl of H_2_O, 120 µl of 10× NEB T4 DNA ligase buffer (NEB B0202), 100 µl of 10% Triton X-100, 12 µl of 10 mg ml^−1^ BSA (100× BSA, NEB) and 5 µl of 400 U µl^−1^ T4 DNA ligase (NEB M0202), and incubating at room temperature for ≥4 h with rotation.

Nuclei were then pelleted by centrifugation at 3,500*g* for 5 min; the pellet was resuspended in 200 µl of chromatin immunoprecipitation elution buffer (1% SDS, 0.1 M NaHCO_3_), Proteinase K was added and the mixture was incubated at 65 °C overnight to reverse crosslinks.

After addition of 600 µl of 1× TE buffer, DNA was sonicated using a Qsonica S-4000 with a 1/16" tip for 3 min, with 10-s pulses at intensity 3.5, and 20 s of rest between pulses. DNA was then purified using the MinElute PCR Purification Kit (Qiagen no. 28006), with elution in a total volume of 300 µl of 1× EB buffer.

For streptavidin pulldown of biotin-labeled DNA, 150 µl of 10 mg ml^−1^ Dynabeads MyOne Streptavidin T1 beads (Life Technologies, 65602) were separated on a magnetic stand, then washed with 400 µl of 1× Tween washing buffer (TWB; 5 mM Tris-HCl pH 7.5, 0.5 mM EDTA, 1 M NaCl, 0.05% Tween 20). The beads were resuspended in 300 µl of 2× binding buffer (10 mM Tris-HCl pH 7.5, 1 mM EDTA, 2 M NaCl), the sonicated DNA was added and the beads were incubated for ≥15 min at room temperature on a rotator. After separation on a magnetic stand, the beads were washed with 600 µl of 1× TWB, and heated at 55 °C in a Thermomixer with shaking for 2 min. After removal of the supernatant on a magnetic stand, the TWB wash and 55 °C incubation were repeated.

Final libraries were prepared on beads using the NEBNext Ultra II DNA Library Prep Kit (NEB, no. E7645) as follows. End repair was carried out by resuspending beads in 50 µl of 1× EB buffer, and adding 3 µl of NEB Ultra End Repair Enzyme and 7 µl of NEB Ultra End Repair Buffer, followed by incubation at 20 °C for 30 min and then at 65 °C for 30 min.

Adapters were ligated to DNA fragments by adding 30 µl of Blunt Ligation mix, 1 µl of Ligation Enhancer and 2.5 µl of NEB Adapter; incubating at 20 °C for 20 min; adding 3 µl of USER enzyme; and incubating at 37 °C for 15 min.

Beads were then separated on a magnetic stand, and washed with 600 µl of TWB for 2 min at 55 °C, 1,000 r.p.m. in a Thermomixer. After separation on a magnetic stand, beads were washed in 100 µl of 0.1× TE buffer, then resuspended in 16 µl of 0.1× TE buffer and heated at 98 °C for 10 min.

For PCR, 5-µl samples of each of the i5 and i7 NEB Next sequencing adapters were added together with 25 µl of 2× NEB Ultra PCR Mater Mix. PCR was carried out with a 98 °C incubation for 30 s and 12 cycles of 98 °C for 10 s, 65 °C for 30 s and 72 °C for 1 min, followed by incubation at 72 °C for 5 min.

Beads were separated on a magnetic stand, and the supernatant was cleaned up using 1× AMPure XP beads.

Libraries were sequenced in a paired-end format on an Illumina NextSeq instrument using NextSeq 500/550 high-output kits (either 2 × 75 or 2 × 36 cycles).

### Hi-C data processing and assembly scaffolding

As an initial step, Hi-C sequencing reads from all libraries were trimmed of adapter sequences, pooled together and processed against the previously published *B. minutum* assembly^8^ using the Juicer pipeline^26^ for analyzing Hi-C datasets (v.1.8.9 of Juicer Tools).

The resulting Hi-C matrices were then used as input to the 3D DNA pipeline^27^ for automated scaffolding with the following parameters: --editor-coarse-resolution 5000 --editor-coarse-region 5000 --polisher-input-size 100000 --polisher-coarse-resolution 1000 --polisher-coarse-region 300000 --splitter-input-size 100000 --splitter-coarse-resolution 5000 --splitter-coarse-region 300000 --sort-output --build-gapped-map -r 10 -i 5000.

Manual correction of obvious assembly and scaffolding errors was then carried out using Juicebox^26^.

After finalizing the scaffolding, Hi-C reads were reprocessed against the new assembly using the Juicer pipeline. This was done individually for each library as well as together for the pooled set of reads.

Data were extracted from the final read matrices using the Juicer suite of tools for Hi-C data analysis.

### Identification of Hi-C domains

Hi-C matrices were first converted to *cool* format using HiCExplorer^22^ ‘hicConvertFormat’ with parameters --inputFormat hic --outputFormath5 and default resolutions. Subsequent HiCExplorer commands were carried out at 10-kb, 25-kb and 50-kb resolutions; the 50-kb domains were used for subsequent analysis as they matched visually apparent domain boundaries best. Matrices were normalized using ‘hicNormalize’ with parameter --normalizesmallest, and corrected using ‘hicCorrectMatrixcorrect’ with parameters --correctionMethod KR. Hi-C domains were computationally identified using the ‘hicFindTADs’ from HiCExplorer with parameter --correctForMultipleTestingfdr. The domains derived from the 50-kb resolution analysis were used for subsequent analyses.

### RNA-seq experiments

Total RNA was isolated following previously described protocols^25^.

RNA-seq libraries were generated after selection of polyadenylated RNA using the Nebnext Poly(A) mRNA Magnetic Isolation Module (NEB E7490) and using the NEBNext Ultra II Directional RNA Library Prep (NEB E7765), following manufacturer’s instructions.

### RNA-seq data analysis

For the analysis of unspliced transcripts, RNA-seq reads were aligned against the original *B. minitum* assembly and annotation using the STAR aligner^28^ (v.2.5.3a) with the following settings: --limitSjdbInsertNsj 10000000 --outFilterMultimapNmax 50 --outFilterMismatchNmax 999 --outFilterMismatchNoverReadLmax 0.04 --alignIntronMin 10 --alignIntronMax 1000000 --alignMatesGapMax 1000000 --alignSJoverhangMin 8 --alignSJDBoverhang 1 --sjdbScore 1 --twopassMode Basic --twopass1readsN -1. The fraction of intronic reads was estimated from the resulting BAM files.

For the purpose of differential expression analysis, reads were aligned against the transcriptome space using Bowtie^29^ (v.1.0.1) with the following settings: -e200-a, and quantified using eXpress^30^ (v.1.5.1). The resulting effective counts were used as input to DESeq2 (ref. ^31^) for differential expression analysis. An adjusted *P* value threshold of 0.05 was used to derive lists of significantly differential genes.

### External RNA-seq datasets

Approximately 5 × 10^7^ cells were collected by centrifugation at 100*g* for 5 min at room temperature. Total RNA was extracted and libraries were constructed for RNA-seq using the TruSeq RNA Library Prep Kit V2 (Illumina) according to the manufacturer protocol. All of the raw sequencing reads are available at the Sequence Read Archive with accession number SRX7258938.

### External RNA-seq data analysis

RNA-seq reads were aligned against the corresponding assemblies using the STAR aligner^28^ (v.2.5.3a) with the following settings: --limitSjdbInsertNsj 10000000 --outFilterMultimapNmax 50 --outFilterMismatchNmax 999 --outFilterMismatchNoverReadLmax 0.04 --alignIntronMin 10 --alignIntronMax 1000000 --alignMatesGapMax 1000000 --alignSJoverhangMin 8 --alignSJDBoverhang 1 --sjdbScore 1 --twopassModeBasic --twopass1readsN -1. As available RNA-seq datasets for *B. minutum* are not strand-specific, the strand orientation of the transcriptome was visualized as follows. Aligned reads were first de novo assembled into transcripts and quantified at the transcript level using Stringtie^32^ (v.1.3.3.b); the orientation of splice junctions serves as a reliable guide for the directionality of these transcripts. Open reading frames were identified for each transcript, and transcripts with open reading frames shorter than 60 amino acids were filtered out of the transcript set. Strand-specific genomic tracks were then generated by assigning to each base pair covered by at least one exon in that set the sum of the TPM (transcript per million transcripts) values of all transcripts it is included in.

### External Hi-C datasets

Hi-C datasets for *Trypanosoma brucei* were obtained from GEO accession GSE118764. Hi-C datasets for *S. pombe* were obtained from GEO accession GSE57316. Hi-C datasets for *Caulobacter vibrioides* CB15 were obtained from GEO accession GSE45966.

### Sequence analysis

Topoisomerase and other replication-related proteins were identified in annotated Marine Microbial Eukaryotic Transcriptome Sequencing Project (MMETSP) transcriptome assemblies using HMMER3.0 (ref. ^33^) and the Pfam 27.0 protein domain database^34^ as previously described^6^.

### Reporting Summary

Further information on research design is available in the Nature Research Reporting Summary linked to this article.

## Online content

Any methods, additional references, Nature Research reporting summaries, source data, extended data, supplementary information, acknowledgements, peer review information; details of author contributions and competing interests; and statements of data and code availability are available at 10.1038/s41588-021-00848-5.

## Supplementary information

Supplementary Information Supplementary Tables 1 and 2, and Figs. 1–33

Reporting Summary

Peer Review Information

## Data Availability

Data associated with this manuscript have been submitted to GEO under accession number GSE153950.

## Code availability

Custom code used to process the data is available at https://github.com/georgimarinov/GeorgiScripts
