# Supplementary material for: Transcription-dependent domain-scale three-dimensional genome organization in the dinoflagellate Breviolum minutum
Source: Nat Genet. 2021 Apr 29;53(5):613–7. doi: 10.1038/s41588-021-00848-5 (PMC8110477; doi:10.1038/s41588-021-00848-5)
Supplement: Supplementary file 1 — Supplementary Tables 1 and 2, and Figs. 1–33 [file 41588_2021_848_MOESM1_ESM.pdf]

nature portfolio

https://doi.org/10.1038/s41588-021-00848-5

Supplementary information

Transcription-dependent domain-scale three-dimensional genome organization in the dinoflagellate *Breviolum minutum*

In the format provided by the authors and unedited

Supplementary Materials

Supplementary Tables

Supplementary Table 1: Summary of Hi-C datasets used in this studyNote that the L142 was not aligned independently against the scaffolded assembly.

Hi-C library	Number raw read pairs	Number Hi-C contacts	Inter-chromosomal	Intra-chromosomal	Short Range (<20Kb)	Long Range (>20Kb)
L142-SSBO1-HIC	534,609,924	220,908,462	n/a	n/a	n/a	n/a
L533-SSBO1_27C_Hi-C	556,089,015	151,618,419	86,874,088	64,714,851	15,980,982	48,733,566
L534-SSBO1_34C_Hi-C	531,461,453	165,231,965	105,854,838	59,340,858	15,152,077	44,188,629
L1240-SSBO1-α_amanitin-0h-Hi-C	111,333,226	34,384,671	17,570,560	16,814,111	11,264,242	5,549,754
L1241-SSB01-α_amanitin-16h-Hi-C-rep1	60,696,609	24,238,281	16,530,785	7,707,496	1,948,364	5,759,040
L1242-SSB01-α_amanitin-16h-Hi-C-rep2	67,376,168	25,551,603	16,785,702	8,765,901	2,733,314	6,032,518
L1243-SSB01-α_amanitin-24h-Hi-C-rep1	81,532,584	29,748,439	17,594,153	12,154,286	5,518,236	6,635,937
L1244-SSB01-α_amanitin-24h-Hi-C-rep2	106,381,220	28,845,306	13,249,756	15,595,550	10,678,732	4,916,732
L1245-SSB01-α_amanitin-48h-Hi-C-rep1	90,180,763	27,045,343	14,627,900	12,417,443	7,996,822	4,420,494
L1246-SSB01-α_amanitin-48h-Hi-C-rep2	78,982,528	22,153,117	10,227,616	11,925,501	9,688,693	2,236,727
L1247-SSB01-α_amanitin_high-48h-Hi-C	110,015,013	28,138,017	12,521,207	15,616,810	13,323,212	2,293,491
L1332-SSB01-α_amanitin-0h-Hi-C-technical_rep	117,543,007	34,089,285	8,856,415	9,967,696	6,932,955	3,034,688
L1332-SSB01-α_amanitin-0h-Hi-C-technical_rep (deeply sequenced)	549,347,830	117,521,684	43,073,422	74,448,262	59,815,340	14,617,472
L1333-SSB01-α_amanitin-48h-Hi-C-rep1-technical_rep	117,821,773	23,654,760	9,625,400	10,156,023	8,219,059	1,936,888
L1334-SSB01-α_amanitin_high-48h-Hi-C-technical_rep	95,662,202	23,944,231	7,954,448	7,390,838	6,704,651	686,125
L1334-SSB01-α_amanitin_high-48h-Hi-C-technical_rep (deeply sequenced)	473,889,681	121,126,963	42,613,255	78,513,708	75,375,032	3,118,515
L1336-SSB01-α_amanitin_high-24h-Hi-C-second_time_course	58,747,402	15,663,160	7,063,048	8,600,112	7,507,098	1,092,920
L1337-SSB01-α_amanitin_high-48h-Hi-C-second_time_course	83,691,617	14,523,464	4,509,911	7,483,408	6,557,471	925,885
L1344-SSB01-α_amanitin/triptolide_0h_NT-Hi-C	79,383,186	23,592,335	8,705,306	5,552,923	3,332,771	2,220,106
L1346-SSB01-triptolide_8h_normal_dose-Hi-C	81,731,190	22,700,096	9,664,851	6,591,405	4,367,366	2,223,978
L1347-SSB01-triptolide_8h_high_dose-Hi-C	112,753,865	28,552,855	12,538,332	7,793,297	5,328,647	2,464,576
L1348-SSB01-Triptolide_24h NT-Hi-C	52,148,987	15,674,551	9,064,518	6,610,033	4,572,399	2,037,564
L1349-SSB01-triptolide_24h_normal_dose-Hi-C	132,715,807	36,745,591	14,682,269	9,235,388	5,978,780	3,256,547
L1350-SSB01-triptolide_24h_high_dose-Hi-C	98,429,444	32,121,298	17,352,686	7,050,890	3,608,073	3,442,753
L1351-SSB01-Triptolide_48h NT-Hi-C	96,846,551	28,296,251	15,850,741	12,445,510	8,950,541	3,494,849
L1352-SSB01-triptolide_48h_normal_dose-Hi-C	85,347,611	25,051,605	10,201,062	8,150,528	5,678,662	2,471,791
L1353-SSB01-triptolide_48h_high_dose-Hi-C	99,978,207	26,572,806	10,201,062	8,150,528	5,678,662	2,471,791
L1859-SSB01-no_denaturation_Hi-C	66,901,271	20,405,394	11,777,718	8,627,676	3,085,753	5,541,871
L1860-SSB01-NT_third_time_course_0h_Hi-C-rep1	63,376,846	23,998,854	14,148,277	9,850,577	4,476,912	5,373,576
L1861-SSB01-NT_third_time_course_48h_Hi-C-rep1	50,110,006	20,240,831	13,072,004	7,168,827	2,969,228	4,199,554
L1862-SSB01-α-amanitin_third_time_course_48h_Hi-C-rep1	34,285,113	13,933,089	9,590,066	4,343,023	1,840,009	2,502,993
L1863-SSB01-Triptolide_third_time_course_48h_Hi-C-rep1	51,692,203	20,258,253	14,483,933	5,774,320	2,425,431	3,348,867
L1864-SSB01-NT_third_time_course_96h_washout_Hi-C-rep1	69,331,722	26,471,641	12,353,321	14,118,320	10,798,300	3,319,746
L1865-SSB01-α-amanitin_third_time_course_96h_washout_Hi-C-rep1	45,055,806	18,126,550	11,283,311	6,843,239	3,809,588	3,033,600
L1866-SSB01-Triptolide_third_time_course_96h_washout_Hi-C-rep1	54,731,637	22,146,724	14,960,287	7,186,437	3,349,384	3,837,015

Supplementary Table 2: Inventory of topoisomerases and some other proteins involved in DNA replication in dinoflagellates and other eukaryotes as annotated by transcriptome assemblies in the MMETSP databses

clade	species	TOP1	TOP2	TOP3	MCM	PCNA	RPA1	RPA2	RPA3	RFC1
Amoebozoa	*Stereomyxa ramosa* Chinc5	1	2	2	6	2	3	0	2	1
Amoebozoa	*Vexillifera* sp. DIVA3 564 2	1	2	2	7	1	2	0	0	1
Apicomplexa	*Lankesteria abbottii* Grappler Inlet BC	1	1	0	12	5	1	0	0	1
Bicosoecid	Bicosoecid sp ms1	1	0	0	3	1	1	1	1	0
Bicosoecid	*Cafeteria roenbergensis* E4 10	1	0	2	6	1	0	0	1	0
Bicosoecid	*Cafeteria* sp. Caron Lab Isolate	1	1	4	15	1	1	0	1	1
Bolidophyte	*Bolidomonas pacifica* CCMP 1866	2	5	7	8	1	1	0	0	1
Chlorarachniophyte	*Bigelowiella natans* CCMP1258.1	1	1	9	3	1	4	1	0	0
Chlorarachniophyte	*Bigelowiella natans* CCMP1259	1	1	6	7	1	4	1	0	1
Chlorarachniophyte	*Bigelowiella natans* CCMP 2755	0	0	4	5	1	4	1	0	1
Chlorarachniophyte	*Bigelowiella natans* CCMP623	1	3	7	9	1	2	1	0	1
Chlorarachniophyte	*Chlorarachnion reptans* CCCM449	2	4	8	11	2	3	1	0	1
Chlorarachniophyte	*Lotharella amoebiformis* CCMP2058	2	6	5	10	1	4	1	0	1
Chlorarachniophyte	*Lotharella globosa* CCCM811	1	2	1	0	1	1	1	1	1
Chlorarachniophyte	*Lotharella oceanica* CCMP622	1	0	0	1	1	2	1	1	1
Chlorarachniophyte	*Norrisiella sphaerica* BC52	1	0	3	0	1	2	1	1	0
Chlorarachniophyte	*Partenskyella glossopodia* RCC365	1	2	1	7	1	3	1	2	1
Chlorophyte	*Bathycoccus prasinos* CCMP1898	1	2	3	9	1	2	0	0	0
Chlorophyte	*Bathycoccus prasinos* RCC716	1	2	3	7	1	3	0	0	1
Chlorophyte	*Chlamydomonas* cf sp CCMP681	1	0	0	5	2	1	0	0	1
Chlorophyte	*Crustomastix stigmata* CCMP3273	1	2	4	10	1	1	1	0	1
Chlorophyte	*Cyanoptyche gloeocystis* SAG4.97	1	0	0	4	1	1	1	0	0
Chlorophyte	*Dolichomastix tenuilepis* CCMP3274	1	1	3	1	2	1	0	1	1
Chlorophyte	*Dunaliella tertiolecta* CCMP1320	1	2	3	10	1	2	0	1	1
Chlorophyte	*Mantoniella antarctica* SL 175	1	8	4	13	1	2	2	1	1
Chlorophyte	*Mantoniella* sp CCMP1436	1	2	1	2	1	1	1	1	1
Chlorophyte	*Micromonas* sp CCMP2099	1	2	2	9	1	2	0	1	1
Chlorophyte	*Micromonas* sp NEPCC29	1	2	3	7	1	2	0	1	1
Chlorophyte	*Micromonas* sp RCC472	1	2	2	7	1	2	1	0	1
Chlorophyte	*Nephroselmis pyriformis* CCMP717	1	4	8	10	1	2	0	1	1
Chlorophyte	*Picochlorum oklahomensis* CCMP2329	1	2	2	6	2	2	1	0	1
Chlorophyte	*Picochlorum* sp. RCC944	1	1	2	6	1	2	0	2	1
Chlorophyte	*Picocystis salinarum* CCMP1897	1	2	1	8	2	2	1	2	1
Chlorophyte	*Polytomella parva* SAG 63 3	1	5	3	18	2	3	0	0	1
Chlorophyte	*Prasinoderma coloniale* CCMP1413	1	2	0	2	1	1	0	0	0
Chlorophyte	*Prasinoderma singularis* RCC927	1	1	1	7	1	1	0	1	1
Chlorophyte	*Pterosperma* sp. CCMP1384	1	0	0	3	1	1	1	1	1
Chlorophyte	*Pycnococcus provasolii* RCC2336	1	1	0	9	1	1	0	0	1
Chlorophyte	*Pycnococcus provasolii* RCC931	1	0	0	7	1	1	0	0	1
Chlorophyte	*Pyramimonas parkeae* CCMP726	1	0	4	7	1	2	1	1	1
Chlorophyte	*Stichococcus* sp RCC1054	1	1	1	8	1	1	0	0	1
Chlorophyte	*Tetraselmis chuii* PLY429	2	0	0	0	0	2	0	1	2
Chlorophyte	*Tetraselmis striata* LANL1001	1	4	4	11	1	2	0	1	1
Choanoflagellata	*Acanthoeca* like sp 10tr	1	3	4	10	1	1	0	1	1
Chromerida	*Chromera velia* CCMP2878	1	1	3	10	2	2	0	0	1
Chromerida	*Vitrella brassicaformis* CCMP3346	1	1	2	9	2	1	0	0	1
Chrysophyte	*Chromulina nebulosa* UTEXLB2642	1	1	1	2	1	1	0	0	1
Chrysophyte	*Dinobryon* sp UTEXLB2267	1	3	0	8	1	1	0	0	1
Chrysophyte	*Mallomonas* Sp CCMP3275	1	2	1	9	1	1	0	1	1
Chrysophyte	*Ochromonas* sp CCMP1393	1	2	2	7	1	1	0	0	1
Chrysophyte	*Paraphysomonas bandaiensis* Caron Lab Isolate	1	2	3	9	2	1	1	1	1
Chrysophyte	*Paraphysomonas imperforata* PA2	0	1	3	6	1	1	1	1	1
Chrysophyte	*Pelagococcus subviridis* CCMP1429	1	1	2	11	1	0	0	0	1
Chrysophyte	*Spumella elongata* CCAP 955 1	1	1	3	10	4	3	0	1	1
Ciliate	*Aristerostoma* sp. ATCC 50986	2	1	1	0	2	1	0	0	2
Ciliate	*Blepharisma japonicum* Stock R1072	0	0	0	7	4	1	0	0	0

Continued on next page

clade	species	TOP1	TOP2	TOP3	MCM	PCNA	RPA1	RPA2	RPA3	RFC1
Ciliate	*Climacostomum virens* Stock W 24	1	2	2	9	3	1	0	0	3
Ciliate	*Condylostoma magnum* COL2	0	0	0	2	0	0	0	0	0
Ciliate	*Euplotes focardii* TN1	1	0	0	5	2	1	0	2	0
Ciliate	*Euplotes harpa* FSP1.4	2	0	5	3	1	0	0	1	0
Ciliate	*Fabrea salina* Unknown	1	1	3	7	2	3	0	0	2
Ciliate	*Favella taraikaensis* FeNarragansettBay	0	1	2	7	3	0	0	0	0
Ciliate	*Litonotus pictus* P1	1	1	2	0	0	0	0	0	0
Ciliate	*Mesodinium pulex* SPMC105	2	13	2	16	9	4	0	0	6
Ciliate	*Myrionecta rubra* CCMP2563	0	1	4	11	1	1	0	1	0
Ciliate	*Platyophrya macrostoma* WH	4	4	4	23	4	6	0	0	3
Ciliate	*Protocruzia adherens* Boccale	3	1	0	9	3	3	0	0	1
Ciliate	*Pseudokeronopsis* sp. OXSARD2	1	1	1	6	1	0	0	1	1
Ciliate	*Strombidinopsis acuminatum* SPMC142	2	6	0	32	10	5	0	0	0
Ciliate	*Strombidinopsis* sp. SopsisLIS2011	1	0	0	8	3	2	0	0	0
Ciliate	*Strombidium* inclinatum S3	1	1	2	8	1	1	0	0	1
Ciliate	*Strombidium rassoulzadegani* ras09	1	0	1	6	1	1	0	1	0
Ciliate	*Tiarina fusus* LIS	1	7	3	16	3	4	2	1	1
Cryptophyte	*Chroomonas mesostigmatica* cf CCMP1168	1	5	4	8	1	2	2	0	1
Cryptophyte	*Cryptomonas curvata* CCAP979 52	2	0	2	0	1	1	0	1	0
Cryptophyte	*Cryptomonas paramecium* CCAP977 2a	3	2	2	5	1	1	0	0	1
Cryptophyte	*Geminigera cryophila* CCMP2564	2	1	5	11	1	2	0	1	2
Cryptophyte	*Geminigera* sp. Caron Lab Isolate	1	3	5	18	1	5	0	1	1
Cryptophyte	*Goniomonas pacifica* CCMP1869	8	4	4	12	1	5	1	3	7
Cryptophyte	*Guillardia theta* CCMP 2712	1	0	2	3	1	1	0	1	0
Cryptophyte	*Hemiselmis andersenii* CCMP644	1	2	5	12	1	2	0	1	1
Cryptophyte	*Hemiselmis rufescens* PCC563	1	0	3	7	1	1	1	1	1
Cryptophyte	*Hemiselmis tepida* CCMP443	3	2	0	3	1	1	1	1	1
Cryptophyte	*Hemiselmis viresens* PCC157	1	0	0	7	1	1	0	1	0
Cryptophyte	*Palpitomonas bilix* NIES 2562	0	1	2	13	4	3	0	1	3
Cryptophyte	*Proteomonas sulcata* CCMP704	0	1	0	3	1	1	0	0	1
Cryptophyte	*Rhodomonas lens* RHODO	2	3	2	2	2	2	0	1	0
Cryptophyte	*Rhodomonas* sp. CCMP768	1	0	1	0	1	1	0	0	0
Diatome	*Amphiprora* sp.	1	4	3	9	1	1	0	0	1
Diatome	*Amphora coffeaeformis* CCMP127	1	1	0	4	1	1	0	0	0
Diatome	*Asterionellopsis glacialis* CCMP134	1	7	1	10	1	1	0	0	1
Diatome	*Astrosyne radiata* 13vi08 1A	1	8	3	6	3	2	0	0	1
Diatome	*Attheya septentrionalis* CCMP2084	1	2	0	9	1	1	0	0	1
Diatome	*Aulacoseira subarctica* CCAP 1002 5	1	2	3	8	2	1	0	0	1
Diatome	*Chaetoceros affinis* CCMP159	1	3	1	8	1	1	0	0	1
Diatome	*Chaetoceros curvisetus*	1	4	4	6	1	3	0	0	1
Diatome	*Chaetoceros debilis* MM31A_1	1	3	1	12	1	1	0	0	1
Diatome	*Chaetoceros neogracile* CCMP1317	1	9	3	10	1	1	0	1	1
Diatome	*Coscinodiscus wailesii* CCMP2513	1	3	6	10	1	1	0	1	1
Diatome	*Craspedostauros australis* CCMP3328	1	0	0	4	0	1	0	0	0
Diatome	*Cyclophora tenuis* ECT3854	1	1	0	3	1	1	0	0	0
Diatome	*Cyclotella meneghiniana* CCMP 338	1	4	3	8	1	1	0	0	1
Diatome	*Cylindrotheca closterium* KMMCC:B 181	3	7	3	14	1	2	0	0	1
Diatome	*Dactyliosolen fragilissimus* Unknown	1	3	3	8	1	1	0	1	1
Diatome	*Ditylum brightwellii* GSO103	1	4	3	11	1	1	0	1	1
Diatome	*Ditylum brightwellii* GSO104	1	4	5	10	1	1	0	1	1
Diatome	*Ditylum brightwellii* GSO105	1	2	3	11	2	1	0	1	1
Diatome	*Entomoneis* sp. CCMP2396	0	1	0	0	1	1	0	0	0
Diatome	*Eucampia antarctica* CCMP1452	1	3	0	5	1	1	1	1	1
Diatome	*Extubocellulus spinifer* CCMP396	1	4	10	13	2	5	3	1	2
Diatome	*Fragilariopsis kerguelensis* L2_C3	1	3	3	11	1	1	2	0	1
Diatome	*Fragilariopsis kerguelensis* L26_C5	1	3	5	22	1	1	3	0	1
Diatome	*Grammatophora oceanica* CCMP 410	1	1	3	5	1	1	0	0	1
Diatome	*Helicotheca tamensis* CCMP826	0	1	0	1	1	1	0	1	0
Diatome	*Leptocylindrus danicus* var. apora B651	3	5	3	0	3	2	0	1	1

Continued on next page

clade	species	TOP1	TOP2	TOP3	MCM	PCNA	RPA1	RPA2	RPA3	RFC1
Diatome	*Leptocylindrus danicus* var. danicus B650	3	11	3	19	1	1	0	1	2
Diatome	*Licmophora paradoxa* CCMP2313	1	1	3	7	1	2	0	0	1
Diatome	*Minutocellus polymorphus* CCMP3303	0	0	0	3	1	1	1	1	0
Diatome	*Minutocellus polymorphus* NH13	2	8	7	21	1	0	1	0	3
Diatome	*Minutocellus polymorphus* RCC2270	1	2	1	7	1	1	1	1	1
Diatome	*Nitzschia punctata* CCMP561	1	2	2	9	1	1	1	1	1
Diatome	*Odontella aurita* isolate 1302 5	1	3	7	11	2	2	1	1	1
Diatome	*Odontella sinensis* Grunow 1884	1	3	0	2	1	1	1	1	1
Diatome	*Proboscia alata* PI_D3	1	7	2	21	1	1	2	0	1
Diatome	*Pseudo-nitzschia australis* 10249_10_AB	1	3	4	8	1	1	1	0	1
Diatome	*Pseudo-nitzschia fradulenta* WWA7	2	11	6	24	4	5	0	0	3
Diatome	*Rhizosolenia setigera* CCMP 1694	1	7	4	18	1	2	0	0	2
Diatome	*Skeletonema dohrnii* SkelB	1	2	0	14	1	1	2	1	1
Diatome	*Skeletonema marinoi* SkelA	1	1	2	7	1	1	2	0	1
Diatome	*Skeletonema menzelii* CCMP793	1	4	4	8	1	1	2	0	1
Diatome	*Stauroneis constricta* CCMP1120	1	0	1	1	1	1	1	0	0
Diatome	*Staurosira complex* sp. CCMP2646	1	3	4	8	1	1	0	1	1
Diatome	*Stephanopyxis turris* CCMP 815	2	0	1	7	3	2	0	1	1
Diatome	*Striatella unipunctata* CCMP2910	4	2	1	6	3	0	1	0	2
Diatome	*Synedropsis recta* cf CCMP1620	1	2	0	1	1	1	1	1	0
Diatome	*Thalassionema frauenfeldii* CCMP 1798	1	5	7	15	1	3	1	1	2
Diatome	*Thalassionema nitzschioides* L26_B	1	3	4	8	1	1	1	1	1
Diatome	*Thalassiosira antarctica* CCMP982	1	4	2	12	1	1	3	1	1
Diatome	*Thalassiosira gravida* GMp14c1	1	1	3	13	1	1	2	1	1
Diatome	*Thalassiosira miniscula* CCMP1093	1	13	6	10	1	1	2	1	1
Diatome	*Thalassiosira oceanica* CCMP1005	1	10	1	10	1	1	0	0	1
Diatome	*Thalassiosira rotula* CCMP3096	1	5	3	11	1	1	2	1	1
Diatome	*Thalassiosira rotula* GSO102	1	3	2	11	1	1	1	1	1
Diatome	*Thalassiosira weissflogii* CCMP1010	1	4	1	9	1	0	1	0	1
Diatome	*Thalassiosira weissflogii* CCMP1336	1	4	1	8	1	0	1	0	1
Diatome	*Thalassiothrix antarctica* L6_D1	1	2	4	6	1	1	0	1	1
Diatome	*Triceratium dubium* CCMP147	0	1	1	1	1	0	1	1	0
Dinoflagellata	*Alexandrium temarense* CCMP1771	3	18	12	45	18	10	3	4	2
Dinoflagellata	*Amphidinium carterae* CCMP1314	2	5	5	8	2	4	0	0	3
Dinoflagellata	*Azadinium spinosum* 3D9	1	12	13	35	11	6	0	0	3
Dinoflagellata	*Brandtodinium nutriculum* RCC3387	1	13	9	30	21	4	0	0	3
Dinoflagellata	*Ceratium fusus* PA161109	1	15	10	18	12	9	1	1	3
Dinoflagellata	*Crypthecodinium cohnii* Seligo	1	6	5	15	2	4	0	0	3
Dinoflagellata	*Dinophysis acuminata* DAEP01	4	15	9	29	13	8	0	0	2
Dinoflagellata	*Durinskia baltica* CSIRO_CS 38	2	12	9	18	9	8	0	0	4
Dinoflagellata	*Gambierdiscus australes* CAWD 149	1	5	0	9	14	6	0	0	2
Dinoflagellata	*Glenodinium foliaceum* CCAP1116_3	2	9	3	23	7	6	0	1	4
Dinoflagellata	*Gonyaulax spinifera* CCMP409	1	2	0	10	10	8	1	1	1
Dinoflagellata	*Heterocapsa rotundata* SCCAP K 0483	2	19	4	12	6	4	0	0	6
Dinoflagellata	*Heterocapsa triquestra* CCMP 448	1	8	5	13	5	4	0	0	3
Dinoflagellata	*Karenia brevis* CCMP2229	1	14	8	10	8	7	0	1	4
Dinoflagellata	*Karenia brevis* SP1	1	14	13	16	6	8	0	1	4
Dinoflagellata	*Karenia brevis* SP3	1	12	9	13	8	10	0	1	4
Dinoflagellata	*Karenia brevis* Wilson	1	14	7	14	9	8	0	2	5
Dinoflagellata	*Karlodinium micrum* CCMP2283	2	9	7	46	13	31	2	0	5
Dinoflagellata	*Kryptoperidinium foliaceum* CCMP1326	4	14	11	64	16	10	1	0	7
Dinoflagellata	*Lingulodinium polyedra* CCMP1738	1	17	8	19	11	11	1	0	3
Dinoflagellata	*Noctiluca scintillans* Unknown	1	7	3	9	1	6	0	1	1
Dinoflagellata	*Oxyrrhis marina*	1	2	5	9	7	3	0	1	2
Dinoflagellata	*Oxyrrhis marina* CCMP1795	0	0	0	0	3	0	0	0	0
Dinoflagellata	*Oxyrrhis marina* LB1974	1	2	4	10	4	2	0	0	2
Dinoflagellata	*Pelagodinium beii* RCC1491	1	8	2	12	11	4	0	0	4
Dinoflagellata	*Peridinium aciculiferum* PAER_2	1	7	5	11	6	5	0	0	3
Dinoflagellata	*Polarella glacialis* CCMP 1383	1	28	5	23	5	5	0	0	8

Continued on next page

clade	species	TOP1	TOP2	TOP3	MCM	PCNA	RPA1	RPA2	RPA3	RFC1
Dinoflagellata	*Prorocentrum minimum* CCMP1329	1	15	6	29	13	6	0	0	3
Dinoflagellata	*Prorocentrum minimum* CCMP2233	1	14	4	29	12	5	0	0	3
Dinoflagellata	*Protoceratium reticulatum* CCCM 535 CCMP 1889	2	20	9	18	11	10	0	0	2
Dinoflagellata	*Pyrodinium bahamense* pbaha01	1	21	8	29	19	11	0	0	3
Dinoflagellata	*Scrippsiella hangoei* like SHHI_4	1	8	6	22	6	16	0	2	2
Dinoflagellata	*Scrippsiella hangoei* SHTV5	1	8	11	14	3	5	0	0	2
Dinoflagellata	*Scrippsiella trochoidea* CCMP3099	1	27	10	38	12	8	1	1	3
Dinoflagellata	*Symbiodinium kawagutii* CCMP2468	0	0	0	0	2	0	0	0	0
Dinoflagellata	*Symbiodinium* sp. C1	1	9	4	9	6	4	0	0	3
Dinoflagellata	*Symbiodinium* sp. C15	1	7	2	12	3	4	1	0	3
Dinoflagellata	*Symbiodinium* sp. CCMP2430	1	7	2	10	7	4	0	0	3
Dinoflagellata	*Symbiodinium* sp. Mp	1	7	3	13	6	3	0	0	3
Dinoflagellata	*Togula jolla* CCCM 725	1	17	3	21	3	6	0	0	4
Discosea	*Mayorella* sp. BSH 02190019	1	3	2	5	1	1	0	1	1
Discosea	*Neoparamoeba aestuarina* SoJaBio B1 5 56 2	3	3	3	12	3	3	0	1	1
Discosea	*Paramoeba atlantica* 621 1 CCAP 1560 9	1	3	2	8	3	2	0	1	1
Discosea	*Pessonella* sp. PRA 29	1	1	3	0	1	5	0	2	3
Discosea	*Stygamoeba regulata* BSH 02190019	3	8	2	7	2	4	0	0	2
Discosea	*Trichosphaerium* sp. Am I 7 wt	2	0	0	1	2	3	0	0	2
Eugelnophyta	*Eutreptiella gymnastica* like CCMP1594	1	1	1	5	2	1	0	1	1
Foraminifera	*Ammonia* sp. Unknown	1	1	3	9	5	2	0	1	1
Foraminifera	*Elphidium margaritaceum* Unknown	1	1	2	8	3	1	1	0	1
Foraminifera	*Rosalina* sp. Unknown	1	0	0	9	5	0	2	0	1
Foraminifera	*Sorites* sp. Unknown	3	3	0	27	12	3	0	0	2
Fungi	*Debaryomyces hansenii* J26	1	0	0	4	0	0	0	0	1
Glaucophyte	*Gloeochaete witrockiana* SAG46_84	2	2	3	9	2	2	1	1	1
Haptophyte	*Calcidiscus leptoporus* RCC1130	1	3	0	7	1	1	1	0	1
Haptophyte	*Chrysochromulina brevifilum* UTEX LB 985	1	2	1	4	1	3	0	1	0
Haptophyte	*Chrysochromulina ericina* CCMP281	2	1	0	10	1	3	1	1	2
Haptophyte	*Chrysochromulina polylepis* CCMP1757	1	3	5	9	1	2	1	1	1
Haptophyte	*Chrysoculter rhomboideus* RCC1486	1	0	0	9	1	0	0	1	0
Haptophyte	*Coccolithus pelagicus* ssp *braarudi* PLY182g	1	3	0	7	1	2	1	1	0
Haptophyte	*Emiliania huxleyi* 374	1	2	1	9	1	1	0	0	0
Haptophyte	*Emiliania huxleyi* 379	1	1	1	0	0	2	0	0	0
Haptophyte	*Emiliania huxleyi* CCMP370	1	3	5	9	0	2	1	1	1
Haptophyte	*Emiliania huxleyi* PLYM219	1	3	4	10	0	2	1	1	1
Haptophyte	*Exanthemachrysis gayraliae* RCC1523	1	2	0	1	1	1	0	1	1
Haptophyte	*Gephyrocapsa oceanica* RCC1303	1	3	5	11	1	1	0	0	1
Haptophyte	*Imantonia* sp. RCC918	3	1	1	4	2	1	1	1	0
Haptophyte	*Isochrysis galbana* CCMP1323	2	5	6	13	2	3	1	0	2
Haptophyte	*Isochrysis* sp. CCMP1244	1	2	5	11	1	1	0	1	1
Haptophyte	*Isochrysis* sp. CCMP1324	1	2	0	12	1	2	1	0	1
Haptophyte	*Pavlova* sp. CCMP459	1	2	1	6	2	1	2	1	1
Haptophyte	*Phaeocystis antarctica* Caron Lab Isolate	3	7	2	12	1	3	2	0	2
Haptophyte	*Phaeocystis* sp. CCMP2710	1	0	1	2	1	1	1	1	1
Haptophyte	*Pleurochrysis carterae* CCMP645	3	2	1	7	1	2	1	1	1
Haptophyte	*Prymnesium parvum* Texoma1	1	6	4	1	1	2	1	1	1
Haptophyte	*Scyphosphaera apsteinii* RCC1455	1	3	1	7	1	2	1	0	1
Heterolobosea	*Percolomonas cosmopolitus* AE 1 ATCC 50343	1	4	2	9	2	2	0	0	1
Heterolobosea	*Percolomonas cosmopolitus* WS	1	3	1	12	1	2	0	0	3
Khakista	*Corethron pennatum* L29A3	2	5	5	16	1	1	1	0	1
Khakista	*Detonula confervacea* CCMP 353	1	3	2	9	1	1	2	1	1
Kinetoplastida	*Neobodo designis* CCAP 1951 1	1	1	4	8	1	1	0	0	1
Labyrinthulida	*Aplanochytrium* sp. PBS07	1	2	1	3	1	1	1	2	1
Labyrinthulida	*Aplanochytrium stocchinoi* GSBS06	1	2	0	7	1	1	1	1	1
Pelagophyte	*Aureococcus anophagefferens* CCMP1850	6	2	3	45	1	2	0	0	1
Pelagophyte	*Aureoumbra lagunensis* CCMP1510	1	2	2	9	1	2	1	0	1
Pelagophyte	*Chrysocystis fragilis* CCMP3189	2	0	2	6	1	1	1	0	1

Continued on next page

Supplementary Table 2 – *Continued from previous page*

clade	species	TOP1	TOP2	TOP3	MCM	PCNA	RPA1	RPA2	RPA3	RFC1
Pelagophyte	*Chrysoreinhardia* sp. CCMP2950	1	2	1	5	0	1	0	0	1
Pelagophyte	*Chrysoreinhardia* sp. CCMP3193	1	3	2	10	1	2	1	0	1
Pelagophyte	*Pelagomonas calceolata* CCMP1756	1	2	1	9	1	1	2	0	1
Pelagophyte	*Sarcinochrysis* sp. CCMP770	0	0	0	2	1	1	1	1	0
Perkinsid	*Perkinsus chesapeaki* ATCC_PRA_65	2	0	0	0	0	0	0	0	0
Perkinsid	*Perkinsus marinus* ATCC50439	1	0	0	1	2	0	0	0	0
Pinguiophyte	*Phaeomonas parva* CCMP2877	1	3	1	5	3	1	0	1	0
Pinguiophyte	*Pinguiococcus pyrenoidosus* CCMP2078	1	2	3	0	1	1	0	1	0
Raphidophyte	*Chattonella subsalsa* CCMP2191	1	3	0	5	1	1	1	0	1
Raphidophyte	*Fibrocapsa japonica* CCMP1661	0	1	1	5	1	1	0	1	0
Raphidophyte	*Heterosigma akashiwo* CCMP2393	1	4	2	11	2	1	0	1	1
Raphidophyte	*Heterosigma akashiwo* CCMP3107	1	7	2	0	1	1	0	0	0
Raphidophyte	*Heterosigma akashiwo* CCMP452	0	1	0	4	1	1	0	0	0
Raphidophyte	*Heterosigma akashiwo* NB	1	6	1	8	1	1	0	1	1
Rhodophyte	*Compsopogon coeruleus* SAG 36.94	1	3	2	11	1	1	0	0	1
Rhodophyte	*Erythrolobus australicus* CCMP3124	1	2	3	0	1	1	0	1	1
Rhodophyte	*Erythrolobus madagascarensis* CCMP3276	1	1	1	3	1	2	0	1	0
Rhodophyte	*Madagascaria erythrocladiodes* CCMP3234	3	4	5	12	1	2	0	1	2
Rhodophyte	*Porphyridium aerugineum* SAG 1380 2	2	1	2	5	1	2	1	0	1
Rhodophyte	*Rhodella maculata* CCMP736	1	3	3	12	1	1	0	0	1
Rhodophyte	*Rhodosorus marinus* CCMP 769	1	8	6	17	0	3	0	0	2
Rhodophyte	*Timspurckia oligopyrenoides* CCMP3278	1	2	4	6	1	2	1	1	1
Silicoflagellates	*Dictyocha speculum* CCMP1381	1	4	2	9	1	2	1	1	1
Silicoflagellates	*Pseudopedinella elastica* CCMP716	1	5	6	9	1	1	1	1	1
Silicoflagellates	*Pteridomonas danica* PT	1	1	1	2	1	1	1	1	0
Silicoflagellates	*Rhizochromulina marina* cf CCMP1243	1	5	2	8	2	2	1	1	1
Synchromophyceae	*Synchroma pusillum* CCMP3072	1	0	1	3	3	1	0	1	1
Syndinian	*Amoebophrya* sp. Ameob2	2	8	1	13	0	1	0	0	0
Thraustochytrid	*Aurantiochytrium limacinum* ATCCMYA1381	1	3	2	9	1	1	0	1	1
Thraustochytrid	*Schizochytrium aggregatum* ATCC28209	1	1	1	4	1	1	0	0	1
Thraustochytrid	*Thraustochytrium* sp. LLF1b	1	2	1	9	1	1	0	1	1
Tubulinid	*Filamoeba nolandi* NC AS 23 1	2	4	1	13	0	3	1	0	1
Tubulinid	*Sexangularia* sp. ATCC50979	0	6	7	14	2	2	1	0	3
Vanellinid	*Vannella robusta* DIVA3 518 3 11 1 6	1	2	3	6	1	2	1	1	1
Vanellinid	*Vannella* sp. DIVA3 517 6 12	6	6	9	13	1	1	0	1	1
Xantophyte	*Vaucheria litorea* CCMP2940	1	2	0	6	1	1	0	1	1

Supplementary Figures

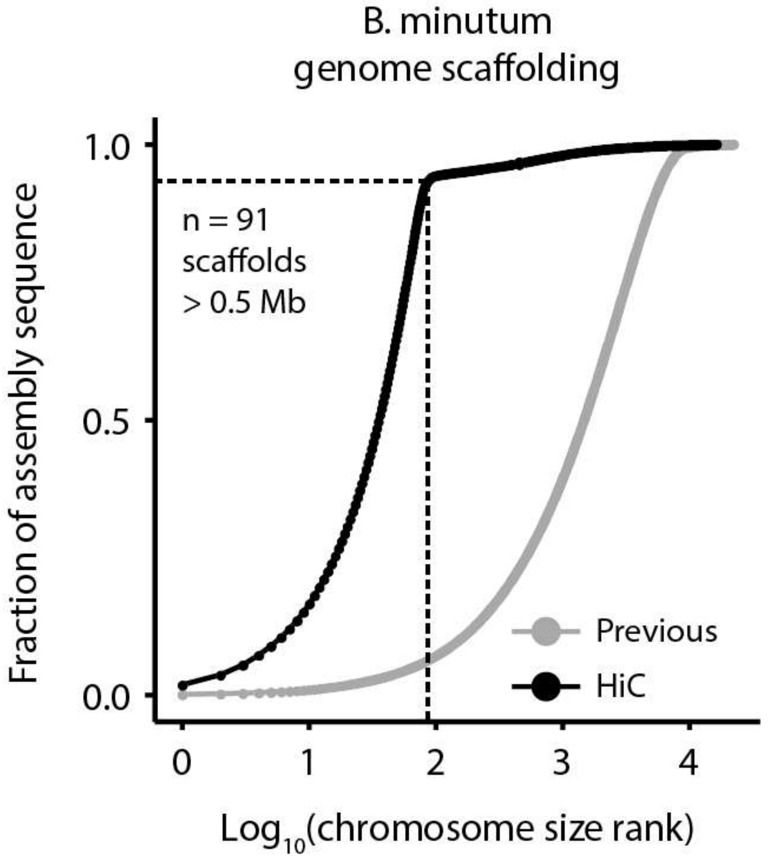

Supplementary Figure 1: Cumulative distribution of scaffolds and pseudochromosome sizes before and after Hi-C scaffolding of the draft *Breviolum minutum* assembly[9]. 3D DNA[27] scaffolding of the assembly results in 91 major pseudochromosomes ≥500kb encompassing ∼94% of the assembled sequence.

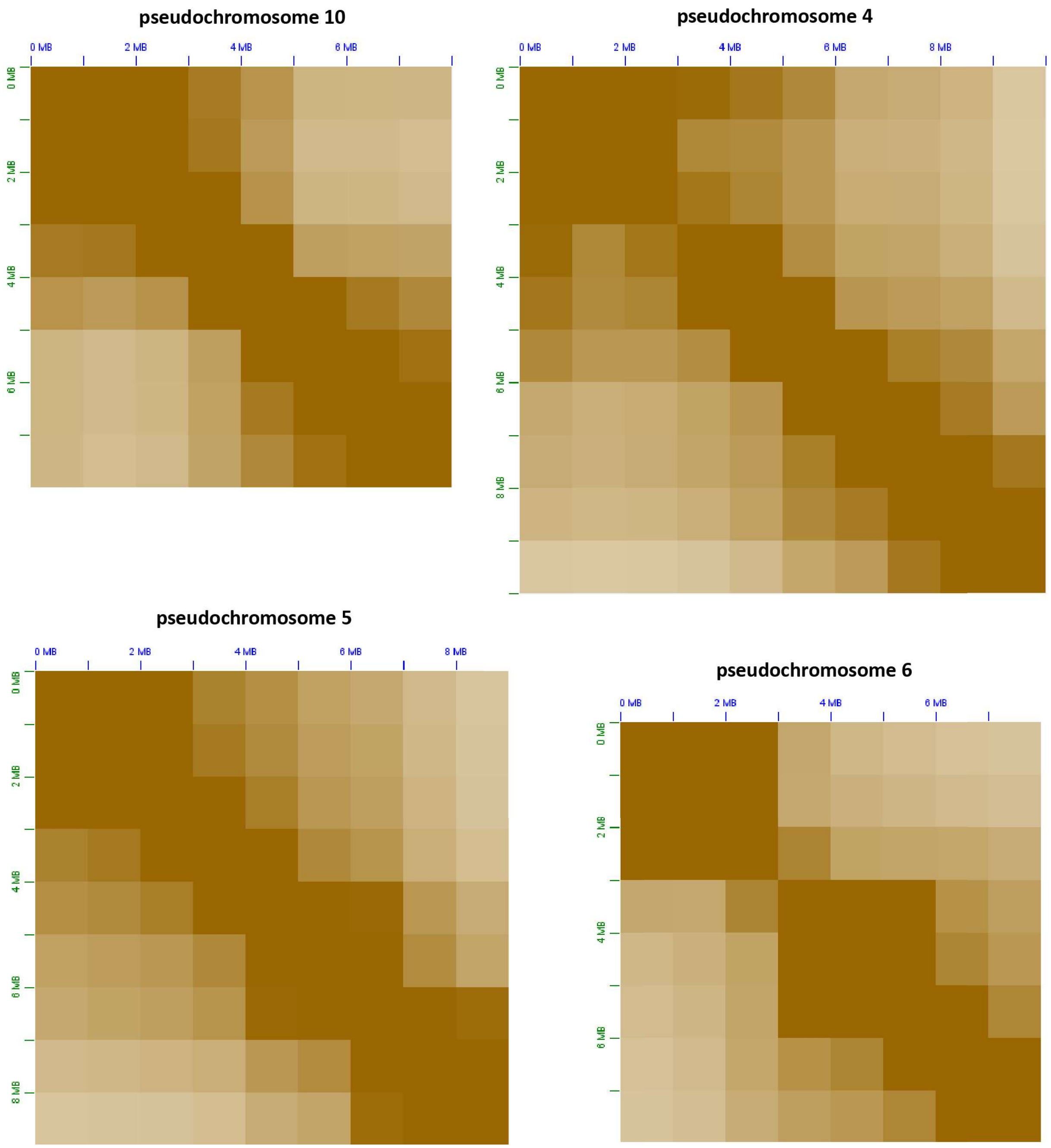

Supplementary Figure 2: Broad-level bipartite to tripartite topological structure of dinoflagellate chromosomes. Shown are 1Mbp-resolution KR-normalized[35] Hi-C matrices for four of the *B. minutum* pseudochromosomes.

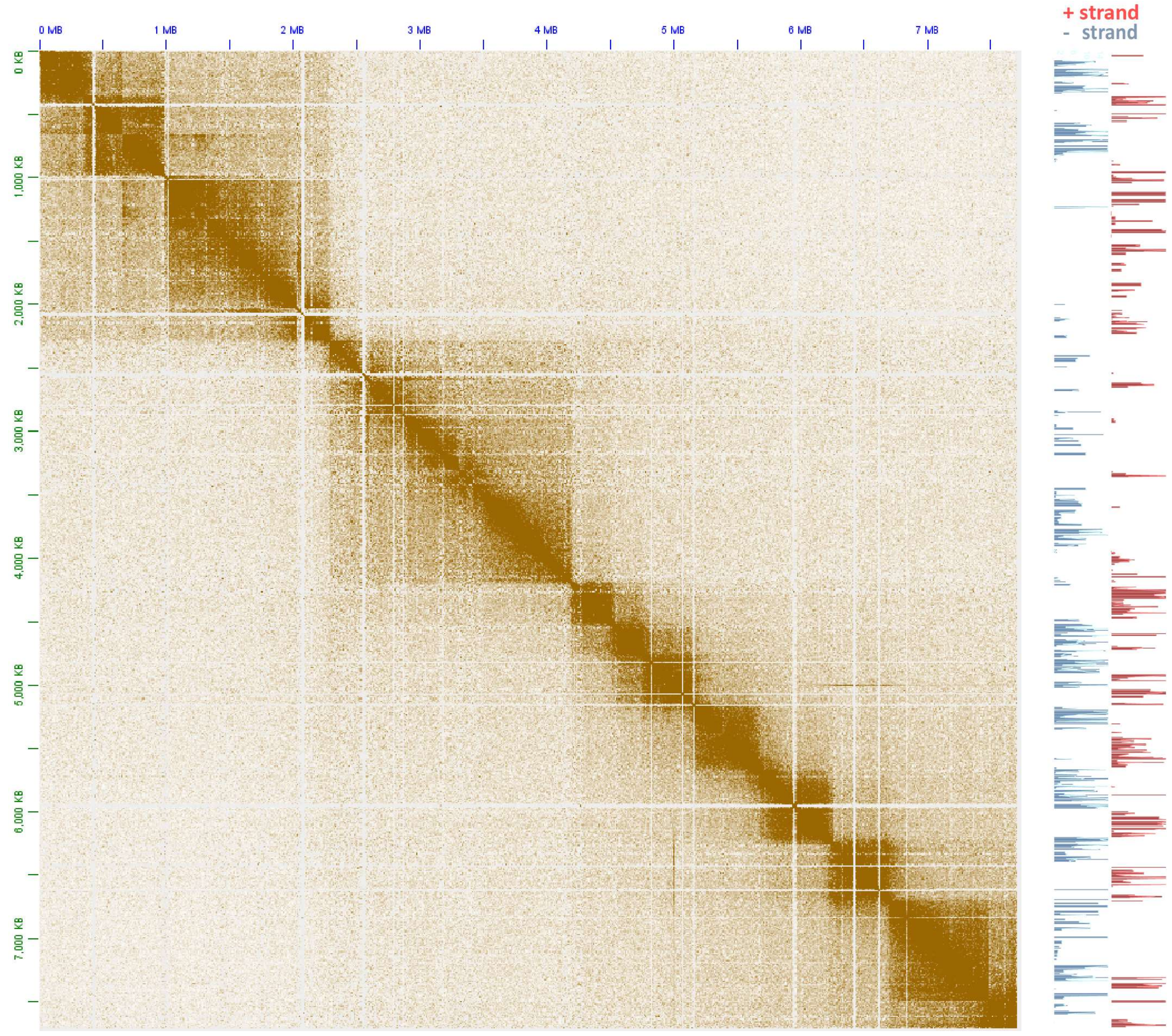

Supplementary Figure 3: The topological domain organization of dinoflagellate chromosomes is related to tandem gene array orientation. Shown is the 5kb-resolution KR-normalized Hi-C map together with strand-specific RNA expression levels for pseudochromosome 17.

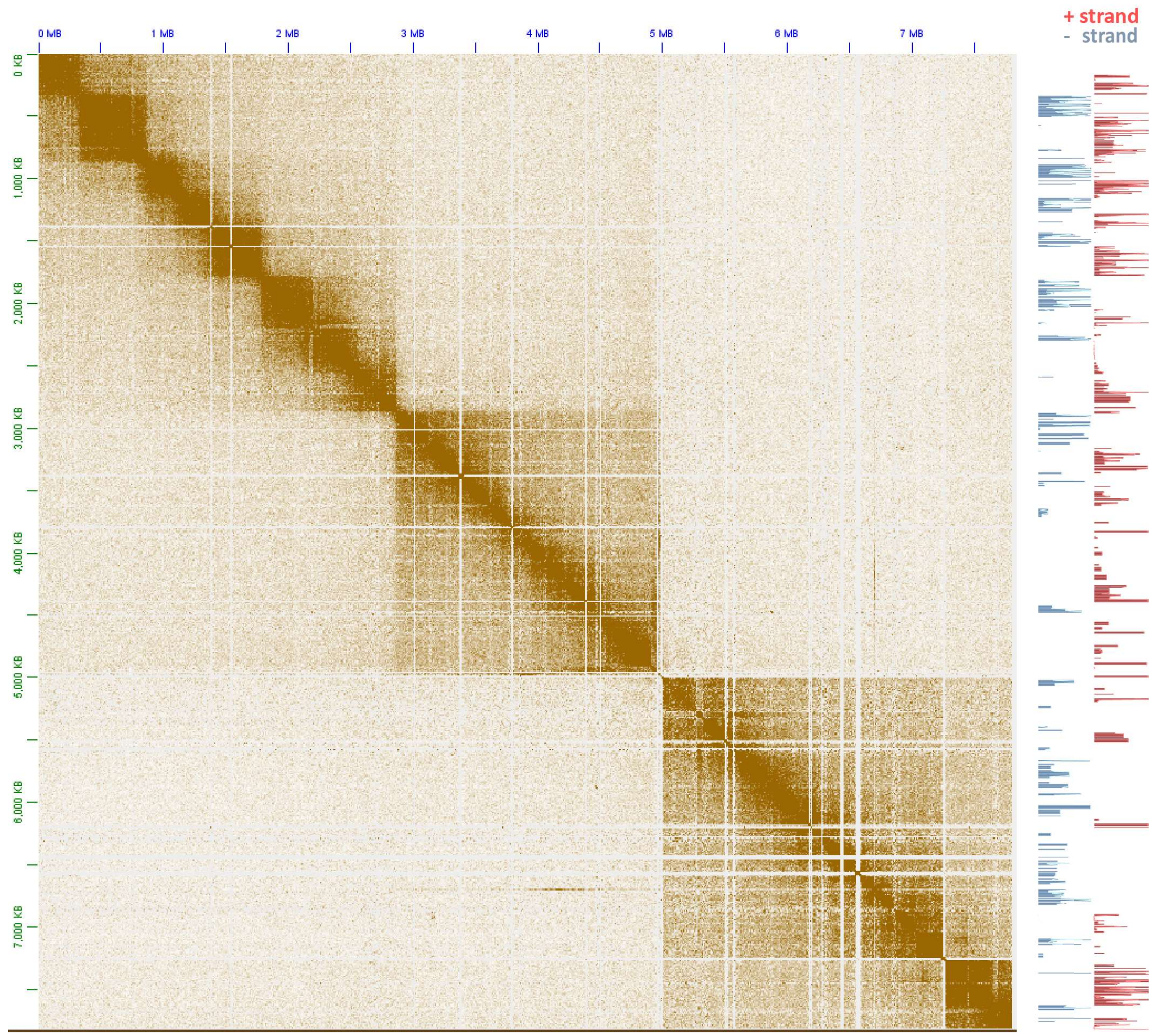

Supplementary Figure 4: The topological domain organization of dinoflagellate chromosomes is related to tandem gene array orientation. Shown is the 5kb-resolution KR-normalized Hi-C map together with strand-specific RNA expression levels for pseudochromosome 18.

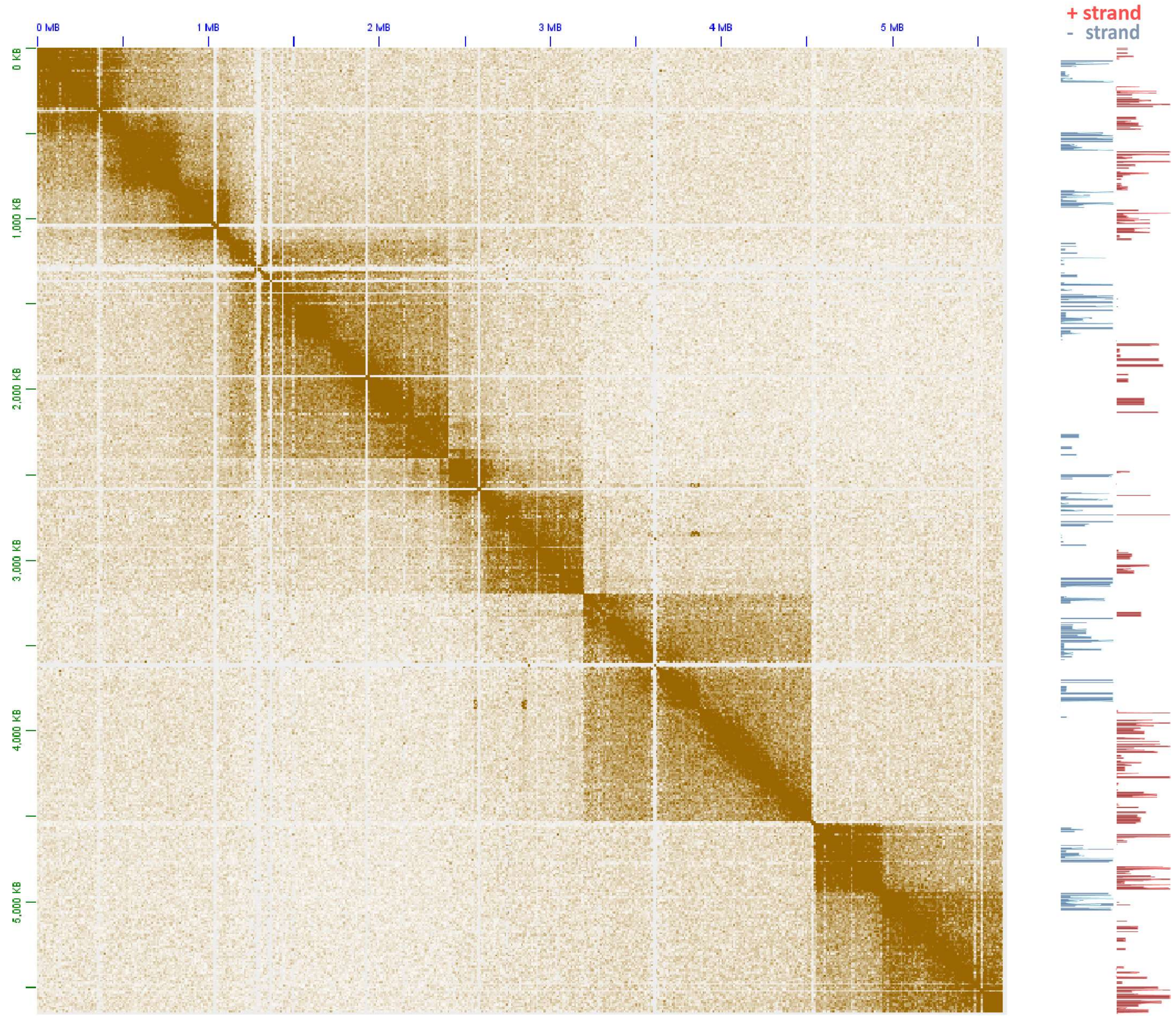

Supplementary Figure 5: The topological domain organization of dinoflagellate chromosomes is related to tandem gene array orientation. Shown is the 5kb-resolution KR-normalized Hi-C map together with strand-specific RNA expression levels for pseudochromosome 21.

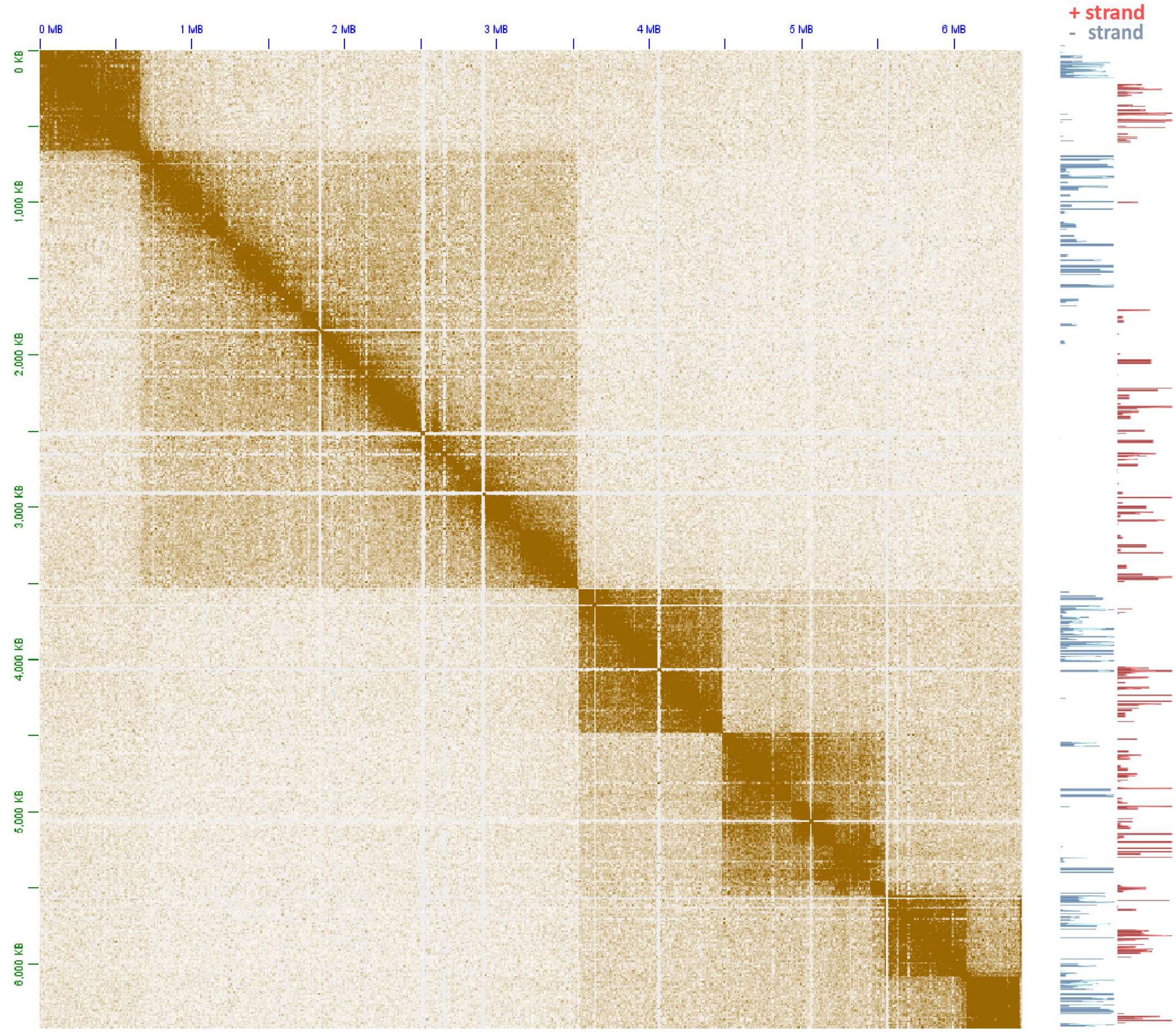

Supplementary Figure 6: The topological domain organization of dinoflagellate chromosomes is related to tandem gene array orientation. Shown is the 5kb-resolution KR-normalized Hi-C map together with strand-specific RNA expression levels for pseudochromosome 26.

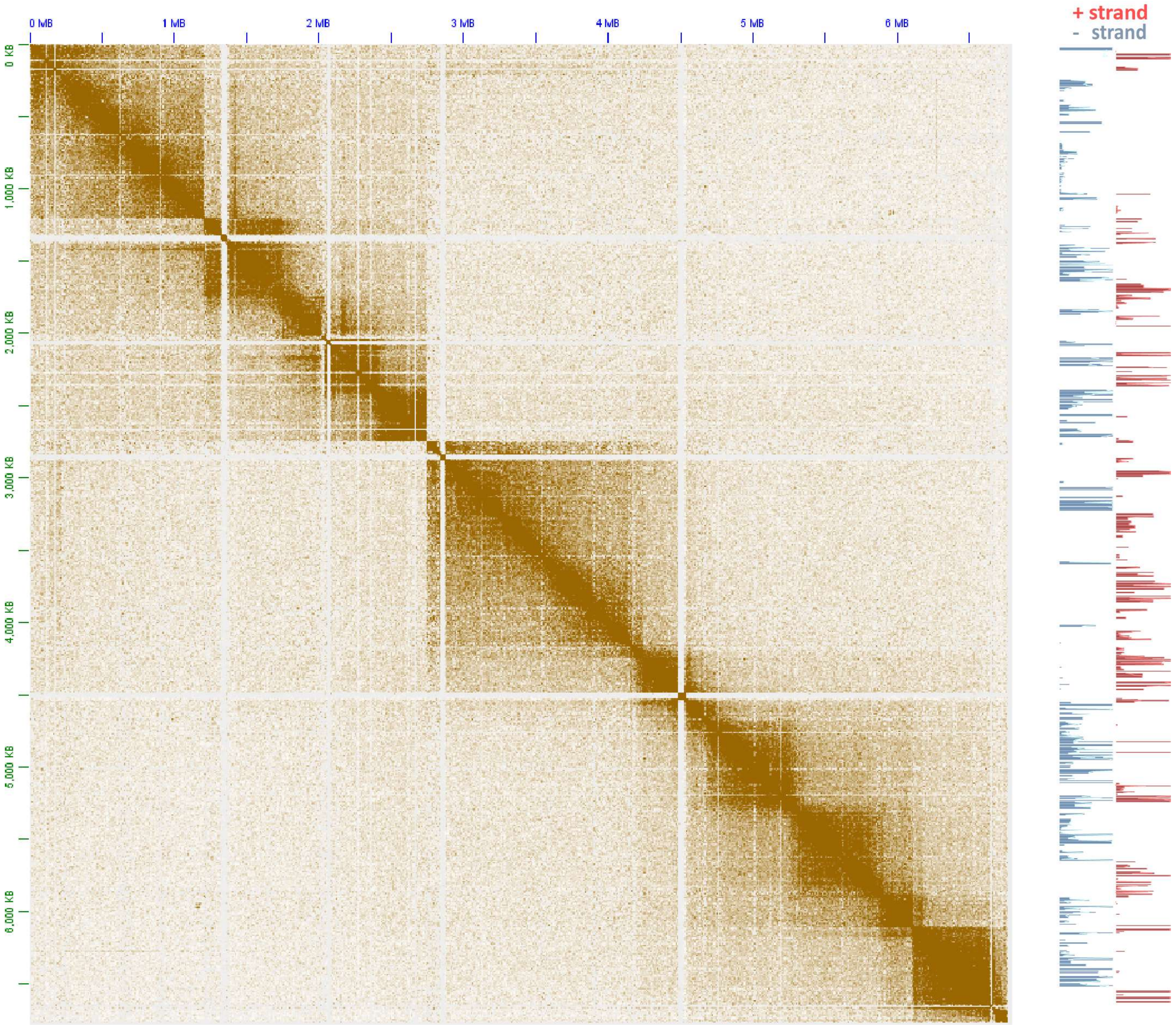

Supplementary Figure 7: The topological domain organization of dinoflagellate chromosomes is related to tandem gene array orientation. Shown is the 5kb-resolution KR-normalized Hi-C map together with strand-specific RNA expression levels for pseudochromosome 32.

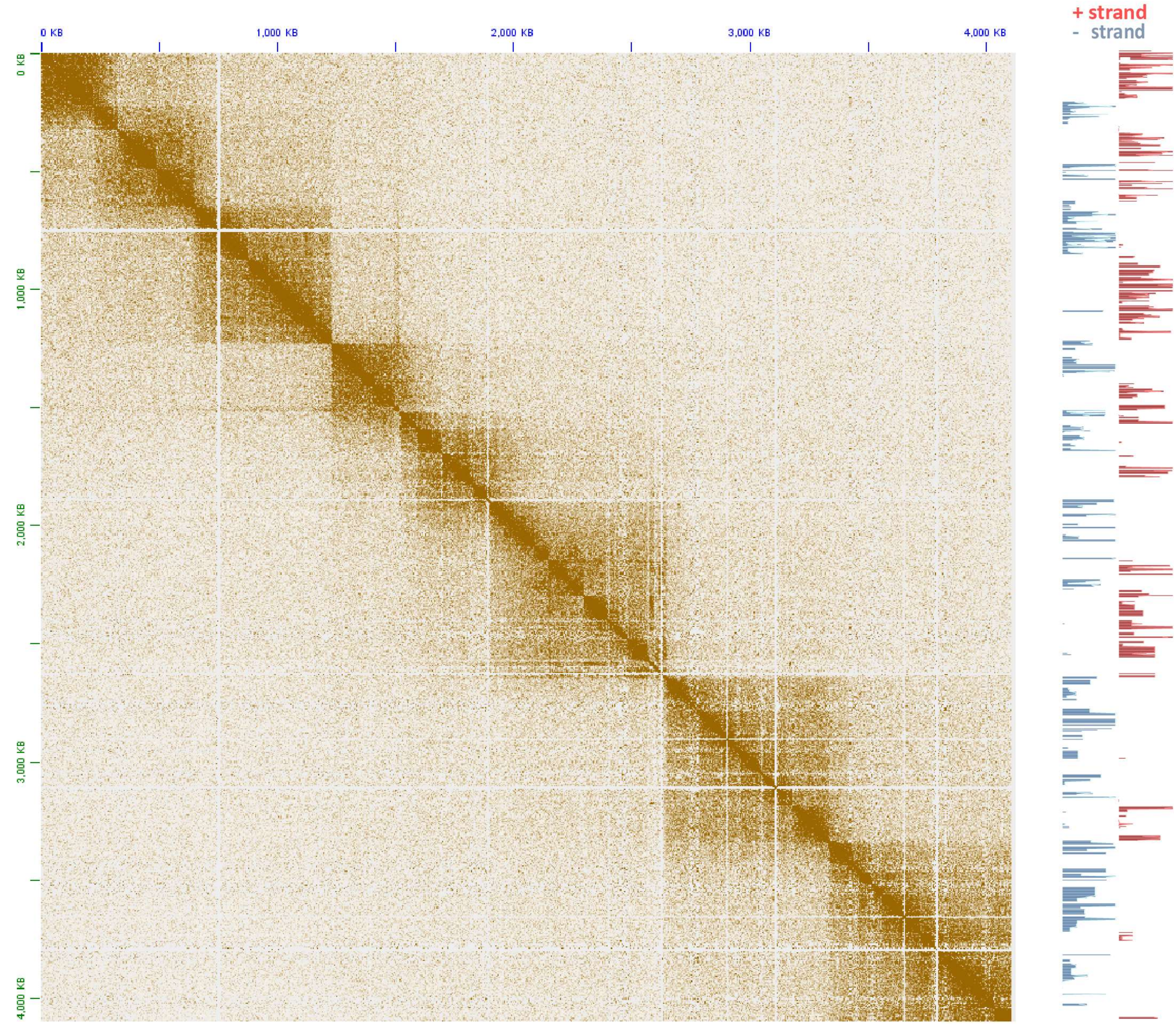

Supplementary Figure 8: The topological domain organization of dinoflagellate chromosomes is related to tandem gene array orientation. Shown is the 5kb-resolution KR-normalized Hi-C map together with strand-specific RNA expression levels for pseudochromosome 36.

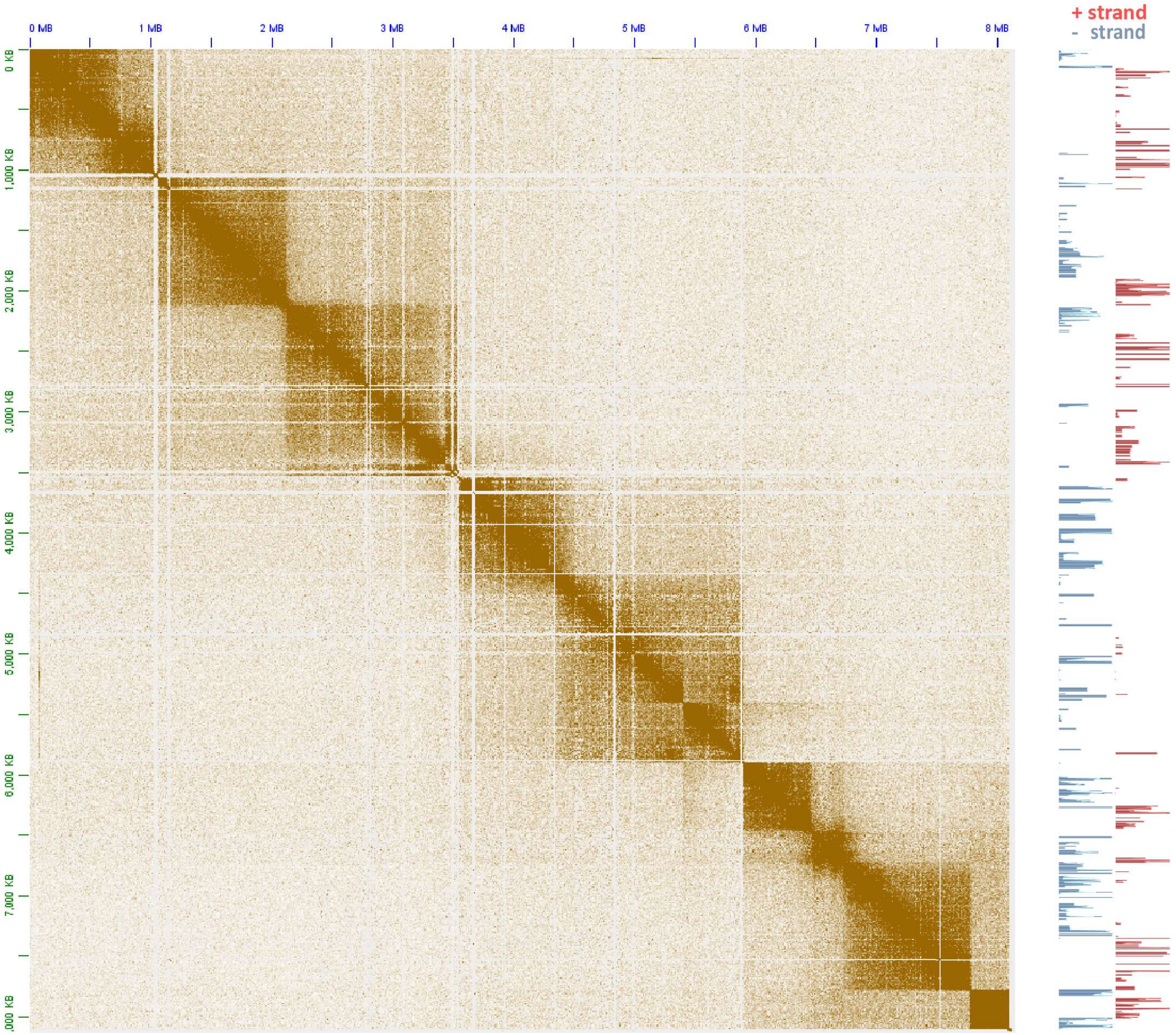

Supplementary Figure 9: The topological domain organization of dinoflagellate chromosomes is related to tandem gene array orientation. Shown is the 5kb-resolution KR-normalized Hi-C map together with strand-specific RNA expression levels for pseudochromosome 71.

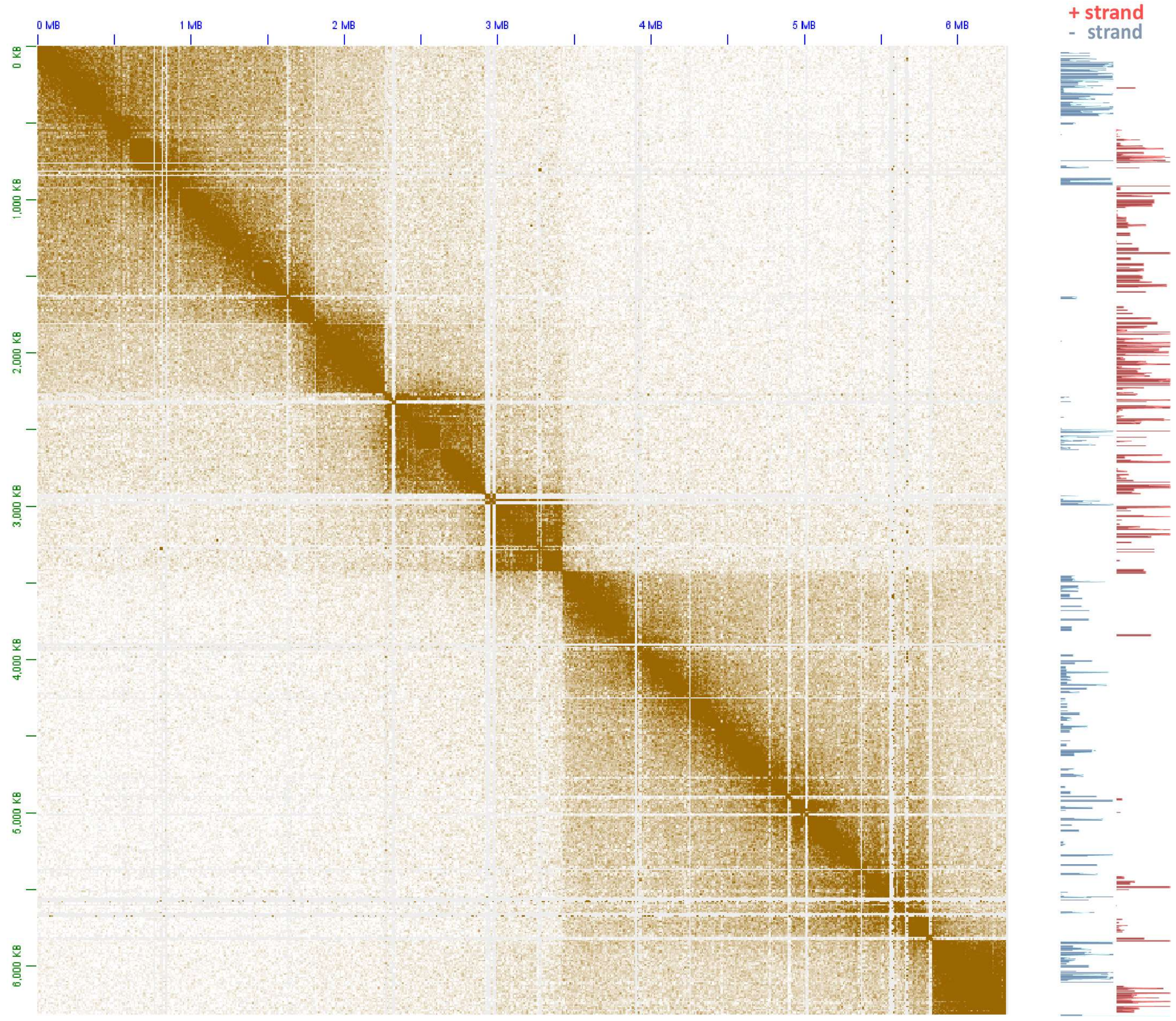

Supplementary Figure 10: The topological domain organization of dinoflagellate chromosomes is related to tandem gene array orientation. Shown is the 5kb-resolution KR-normalized Hi-C map together with strand-specific RNA expression levels for pseudochromosome 77.

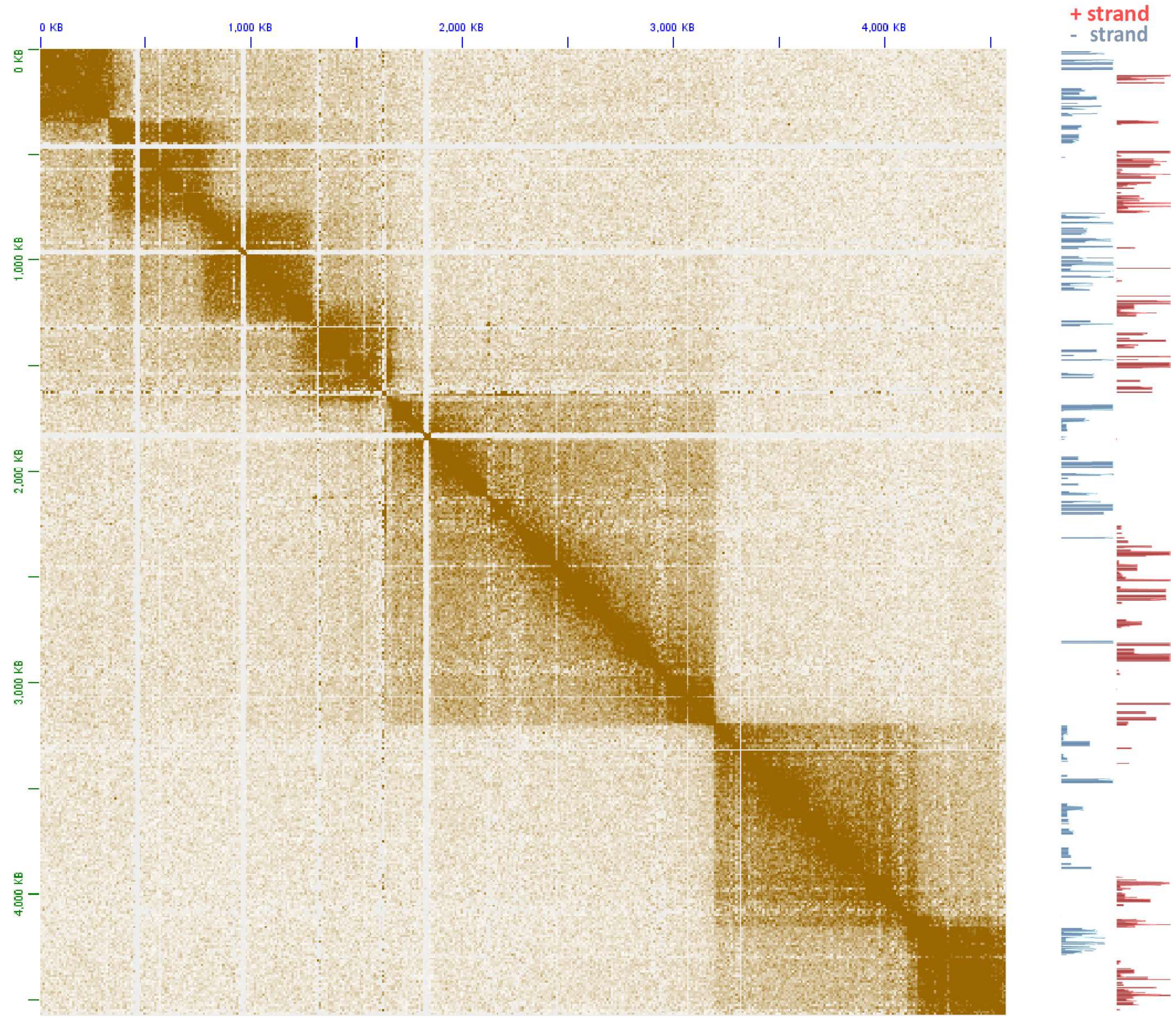

Supplementary Figure 11: The topological domain organization of dinoflagellate chromosomes is related to tandem gene array orientation. Shown is the 5kb-resolution KR-normalized Hi-C map together with strand-specific RNA expression levels for pseudochromosome 78.

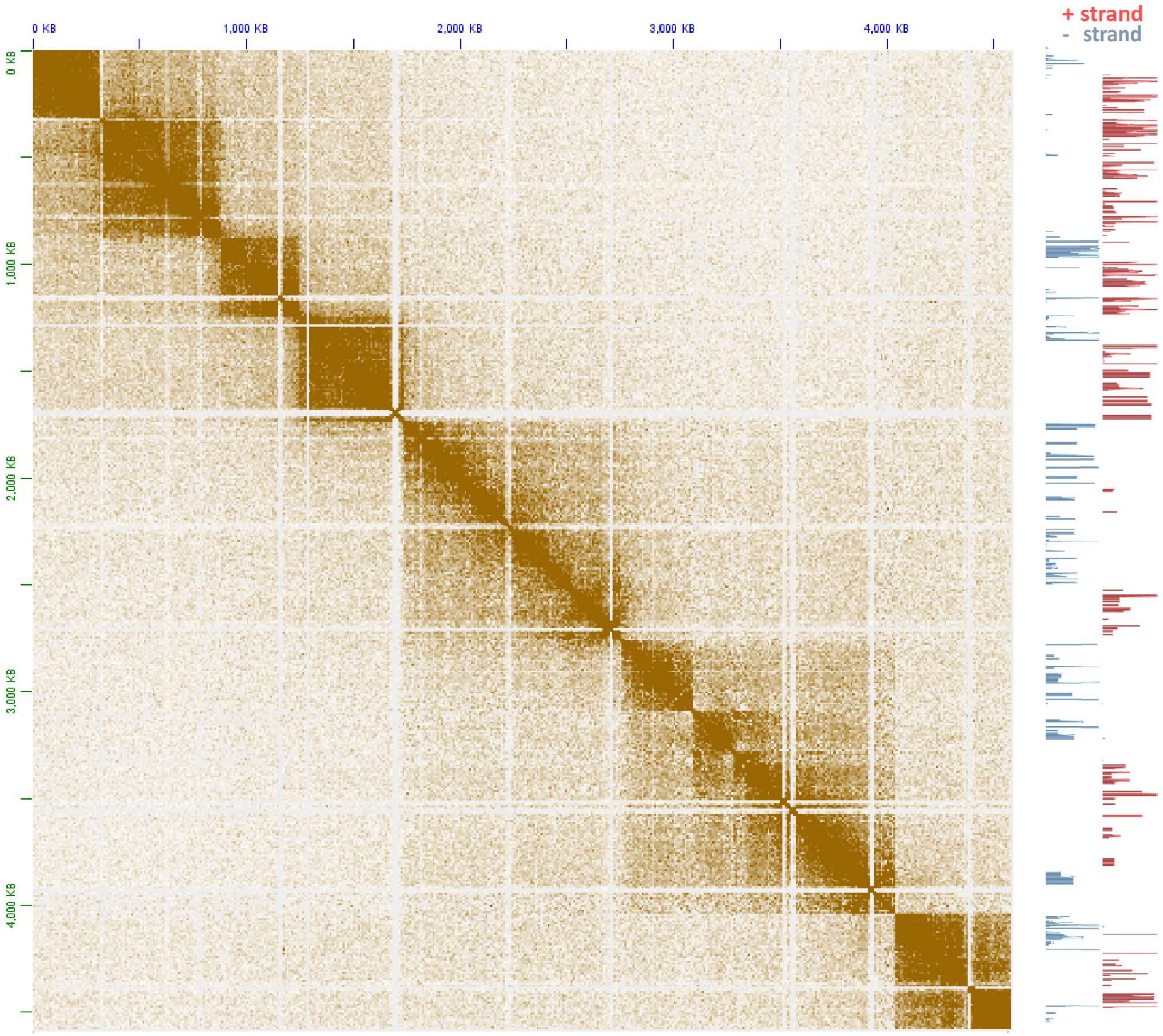

Supplementary Figure 12: The topological domain organization of dinoflagellate chromosomes is related to tandem gene array orientation. Shown is the 5kb-resolution KR-normalized Hi-C map together with strand-specific RNA expression levels for pseudochromosome 88.

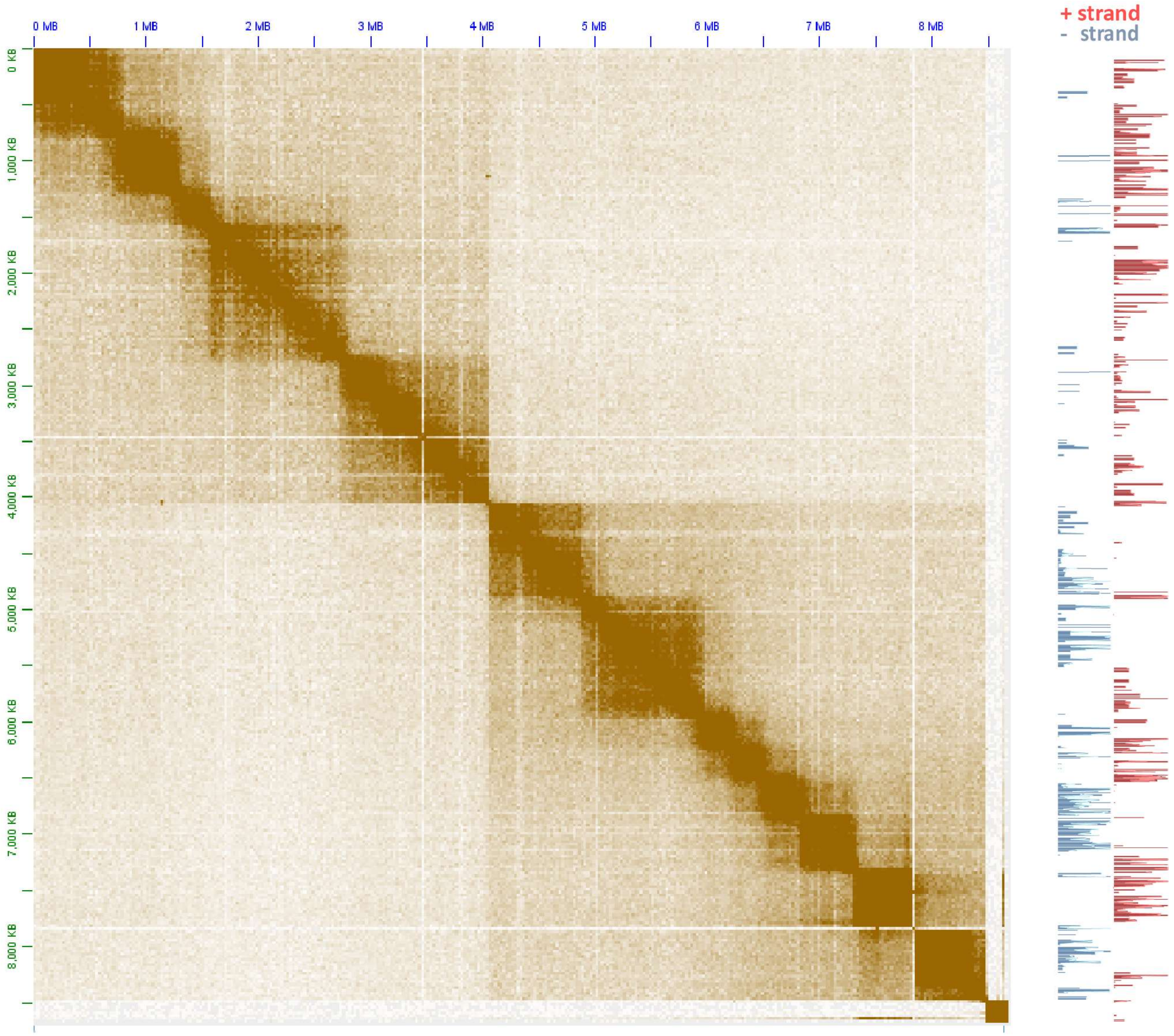

Supplementary Figure 13: The topological domain organization of dinoflagellate chromosomes is related to tandem gene array orientation. Shown is the 5kb-resolution KR-normalized Hi-C map together with strand-specific RNA expression levels for pseudochromosome 89.

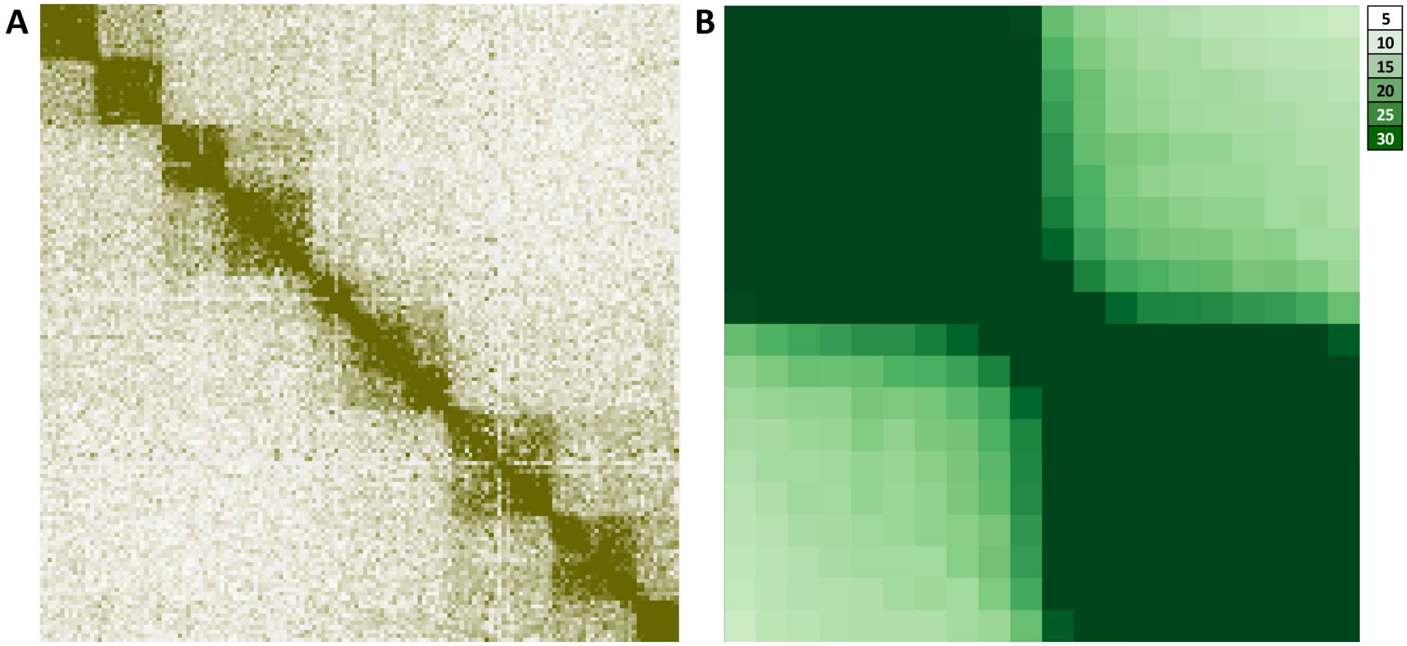

Supplementary Figure 14: DinoTADs become more strongly defined in Hi-C datasets generated by omitting the SDS denaturation step (sample "L1859" in Supplementary Table 1). (A) Snapshot of pseudochromosome 10 at 50-kbp resolution. (B) Metaplot across all dinoTAD boundaries at 50-kbp resolution (drawn to same scale as metaplots in main figures and elsewhere in the supplement)

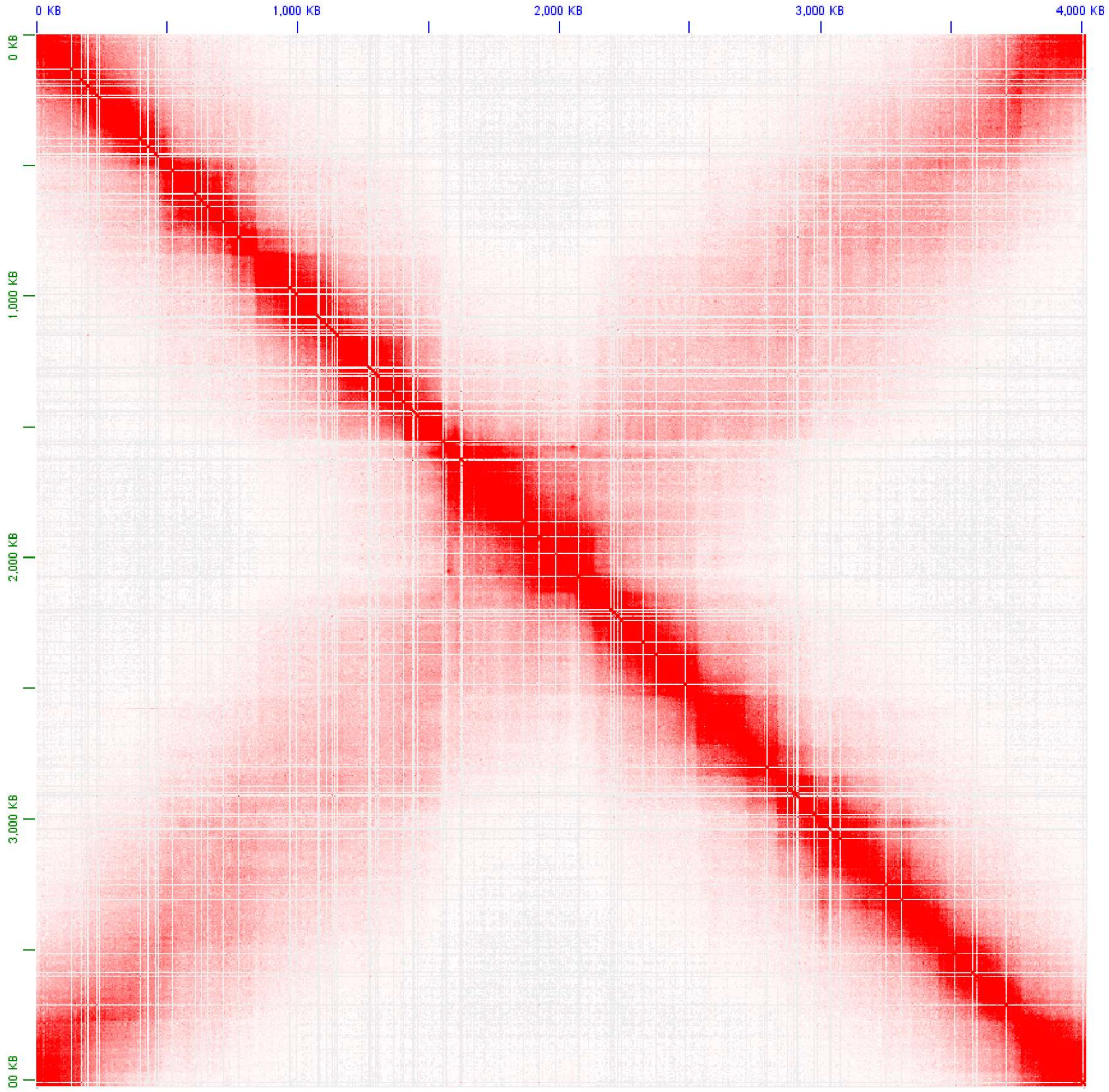

Supplementary Figure 15: Topological structure of the *Caulobacter crescentus* CB15 genome. Shown is the KR-normalized 5-kb resolution maps for the whole *Caulobacter* chromosome (GEO accession GSM1120448).

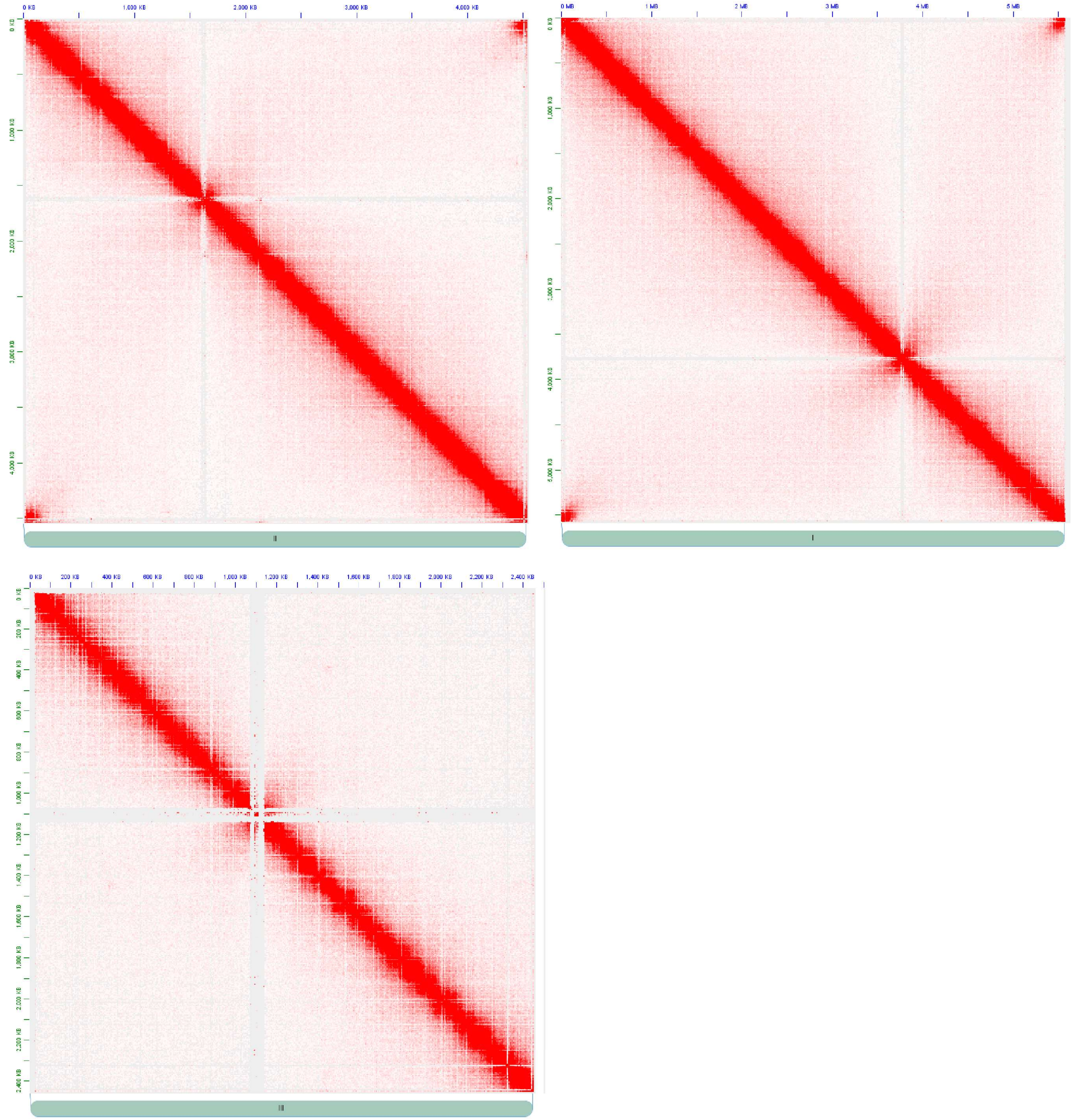

Supplementary Figure 16: Topological structure of the *Schizosaccharomyces pombe* genome. Shown are the KR-normalized 5-kb resolution maps for all three *S. pombe* chromosome (GEO accession GSM1379427).

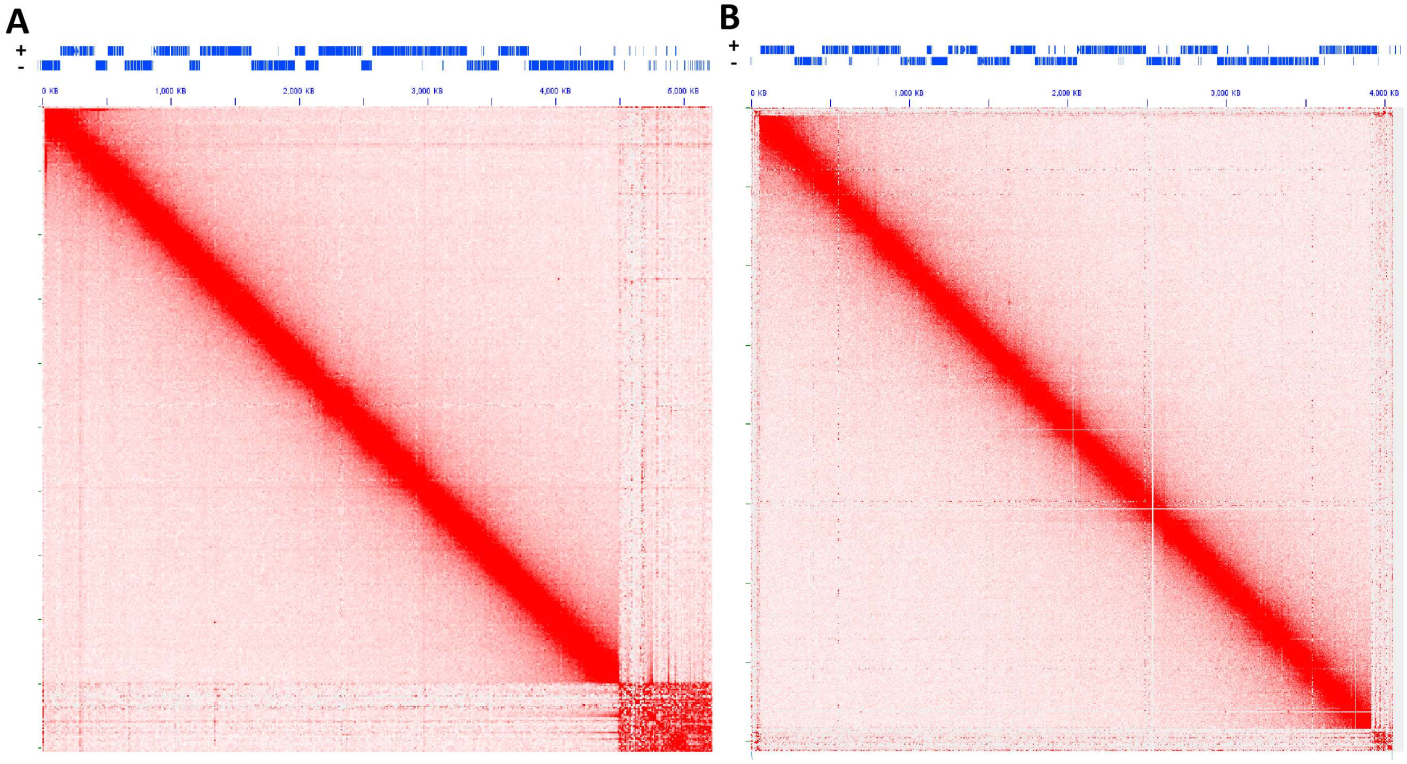

Supplementary Figure 17: No topological domains associated with gene arrays are observed in the kinetoplastid *Trypanosoma brucei*. Shown are KR-normalized 10-kb resolution maps for chr11 (A) and chr10 (B) for GEO accession GSM3346690.

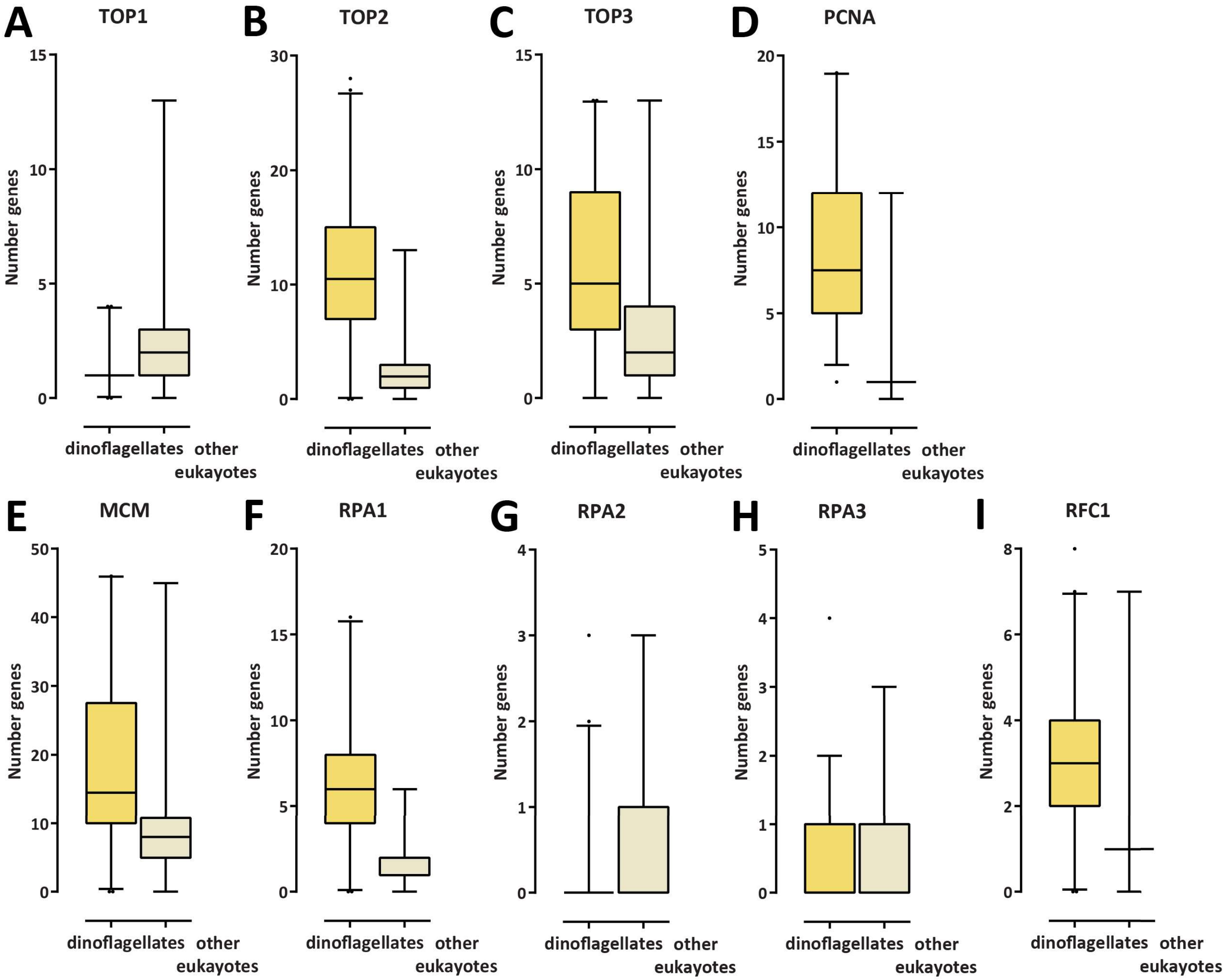

Supplementary Figure 18: Expansion of the Type II and II topoisomerase gene repertoire as well as of certain other replication-related (see Hou et al.[36] for more details) proteins in dinoflagellates. Shown are the number of genes annotated in MMETSP transcriptome assemblies of dinoflagellates and other eukaryotes. (A) Number of Type I topoisomerase genes; (B) Number of Type II topoisomerase genes; (C) Number of Type III topoisomerase genes; (D) Number of PCNA genes; (E) Number of MCM genes; (F) Number of RPA1 genes; (G) Number of RPA2 genes; (H) Number of RPA3 genes; (I) Number of RFC1 genes. Box plots show the 25th, 50th and 75th percentiles, whiskers show the 5-95% intervals. The dinoflagellate (n=41) and non-dinoflagellate (n=243) species shown are the ones from Supplementary Table 2.

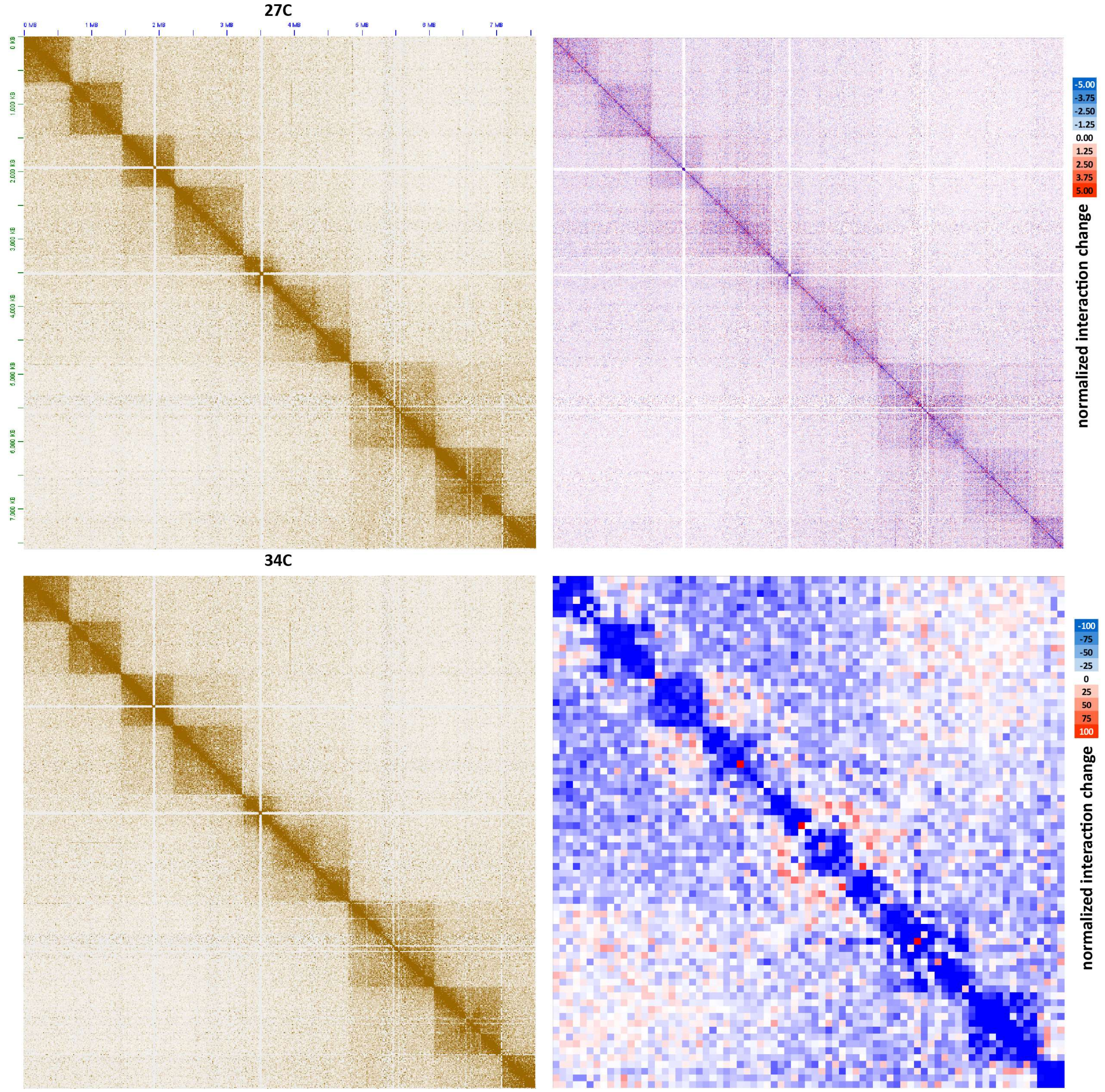

Supplementary Figure 19: Moderate decompaction of dinoTADs upon exposure to elevated temperatures. Shown is pseudochromosome 10 (KR-normalized) and the difference between the KR-normalized Hi-C maps generated from *B. minutum* grown at 34 °C and at 27 °C at 100-kb resolution (lower right) and 5-kb resolution (upper right).

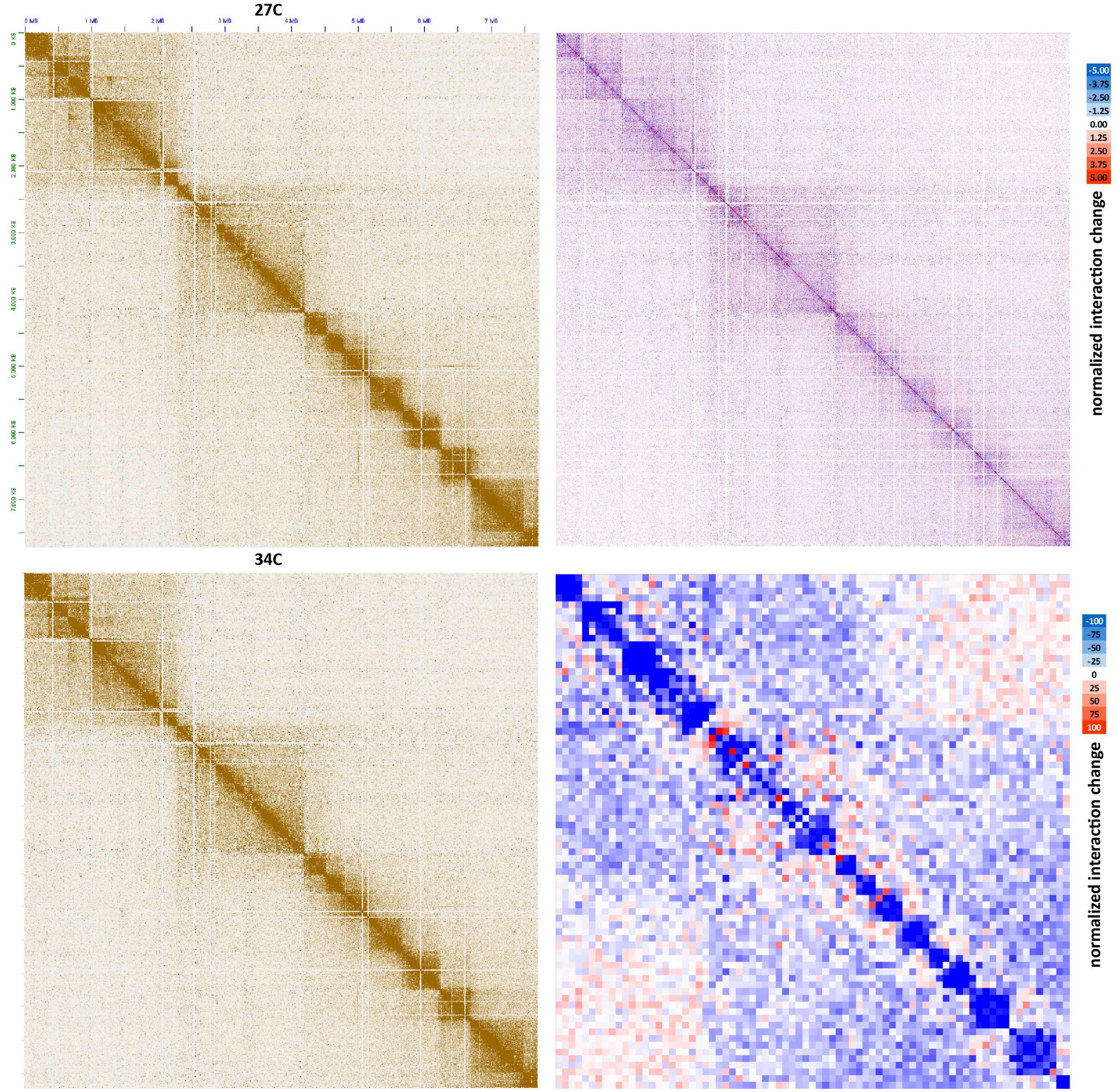

Supplementary Figure 20: Moderate decompaction of dinoTADs upon exposure to elevated temperatures. Shown is pseudochromosome 17 (KR-normalized) and the difference between the KR-normalized Hi-C maps generated from *B. minutum* grown at 34 °C and at 27 °C at 100-kb resolution (lower right) and 5-kb resolution (upper right).

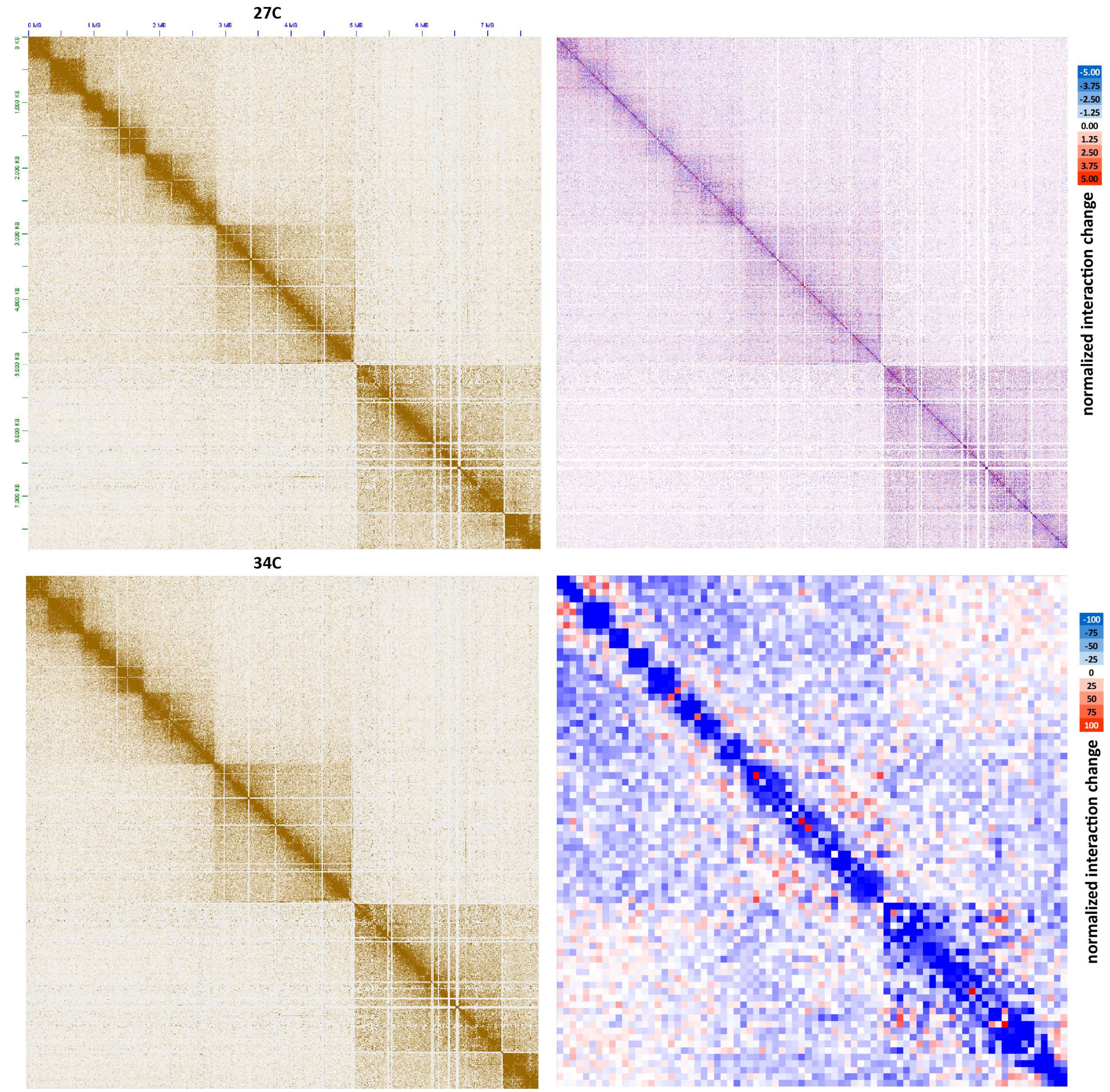

Supplementary Figure 21: Moderate decompaction of dinoTADs upon exposure to elevated temperatures. Shown is pseudochromosome 18 (KR-normalized) and the difference between the KR-normalized Hi-C maps generated from *B. minutum* grown at 34 °C and at 27 °C at 100-kb resolution (lower right) and 5-kb resolution (upper right).

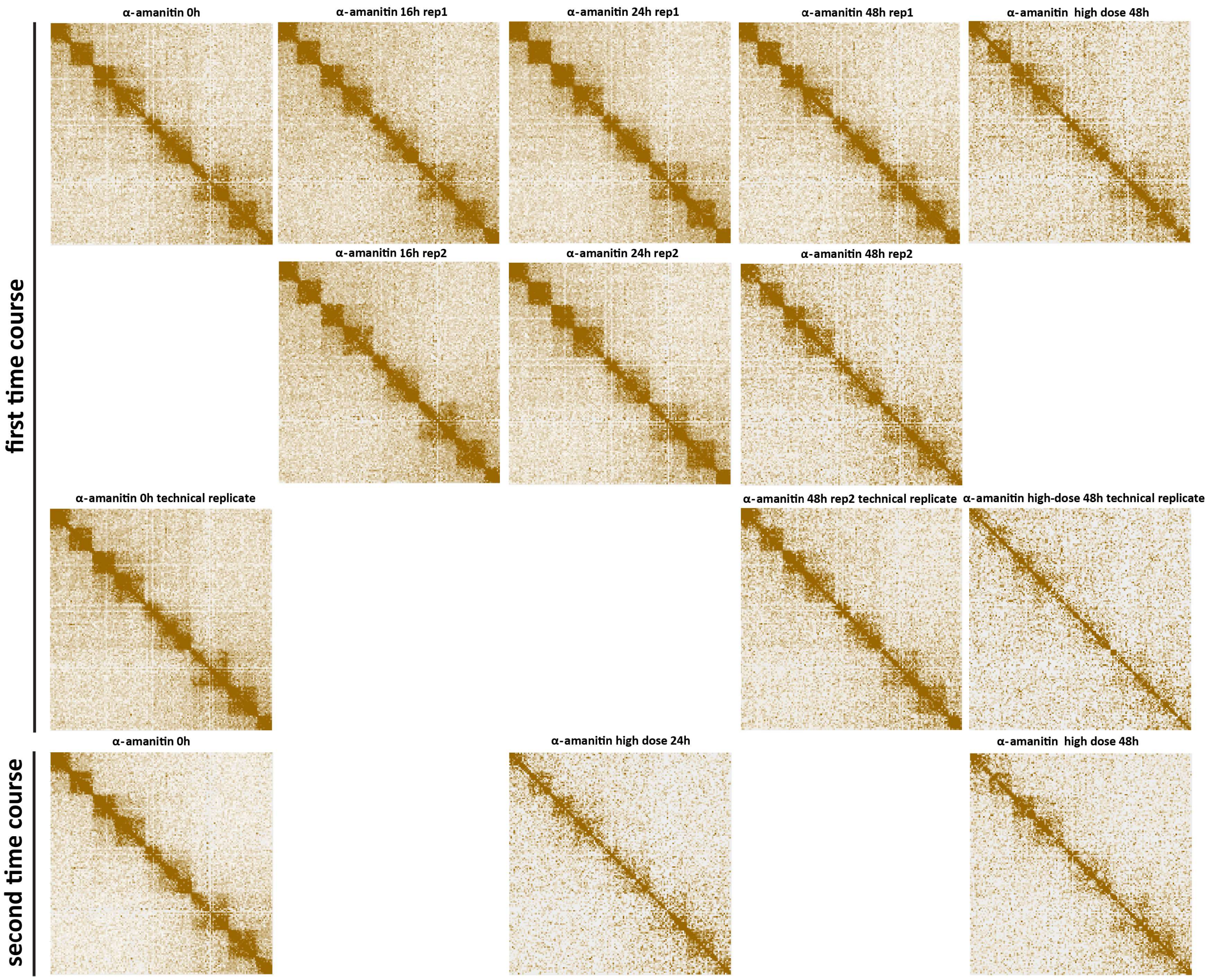

Supplementary Figure 22: Decompaction of dinoTADs upon transcriptional inhibition using α-amanitin. Shown is pseudochromosome 10. Two time courses were carried out following the outline presented in Figure 2B.

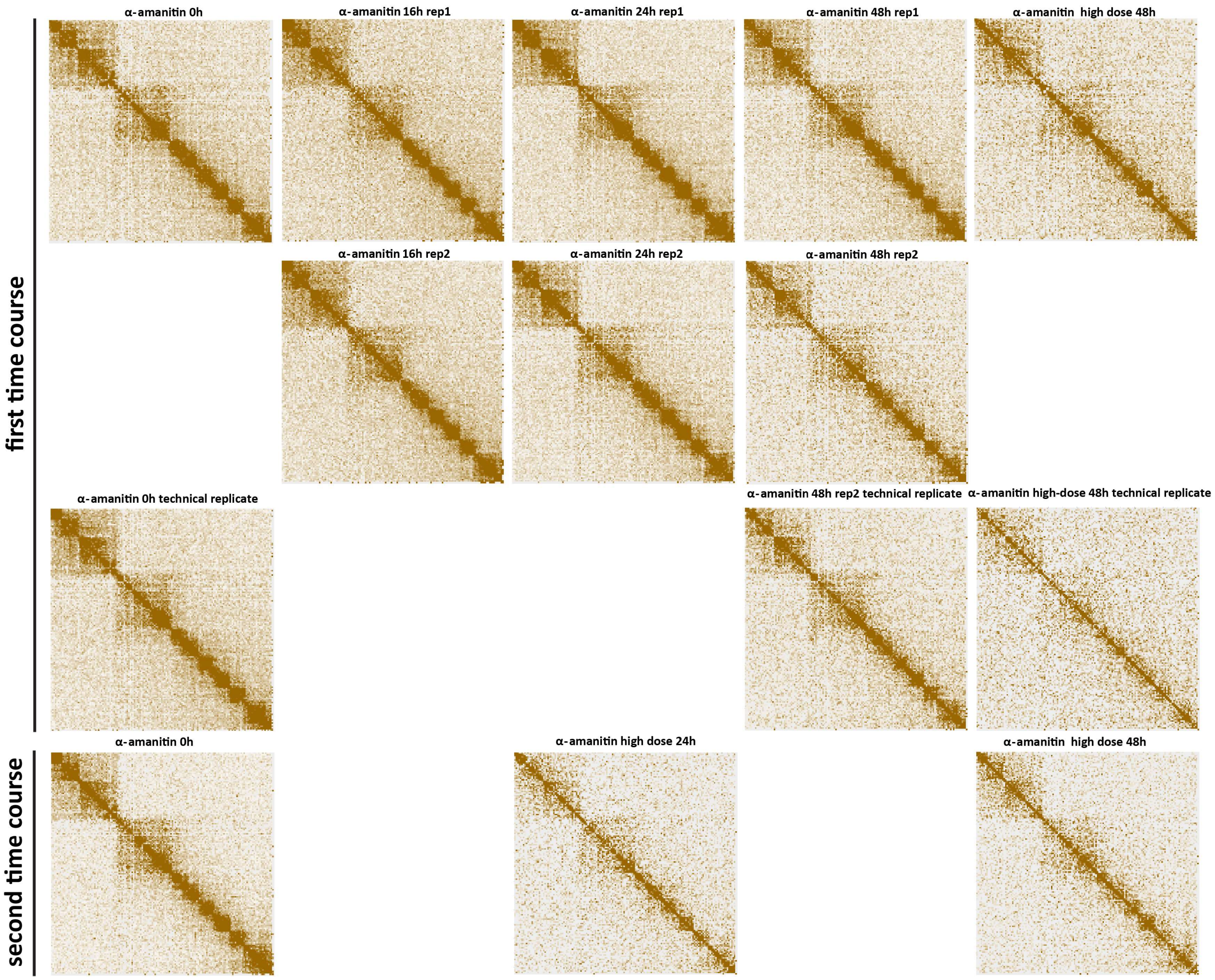

Supplementary Figure 23: Decompaction of dinoTADs upon transcriptional inhibition using α-amanitin. Shown is pseudochromosome 17. Two time courses were carried out following the outline presented in Figure 2B.

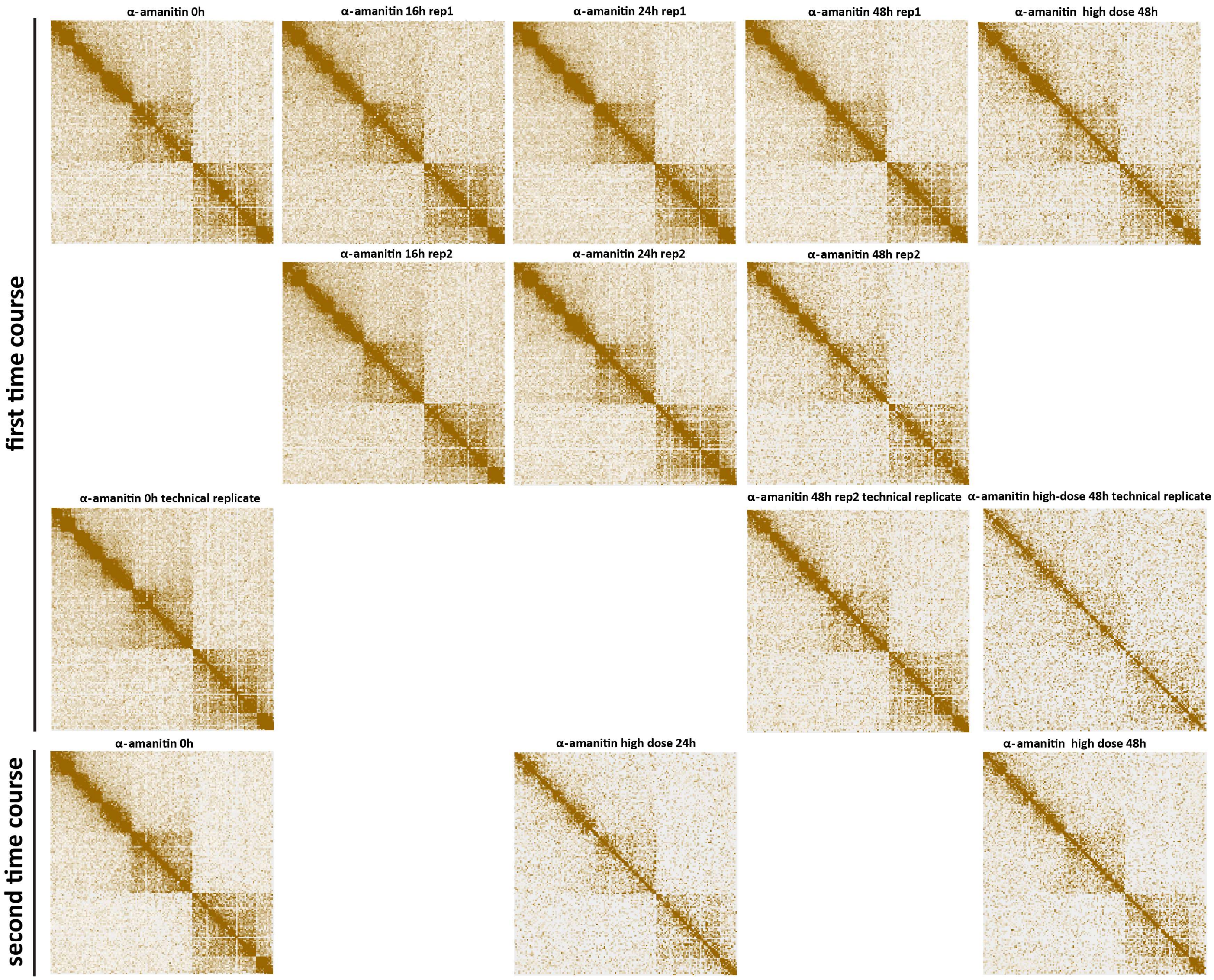

Supplementary Figure 24: Decompaction of dinoTADs upon transcriptional inhibition using α-amanitin. Shown is pseudochromosome 18. Two time courses were carried out following the outline presented in Figure 2B.

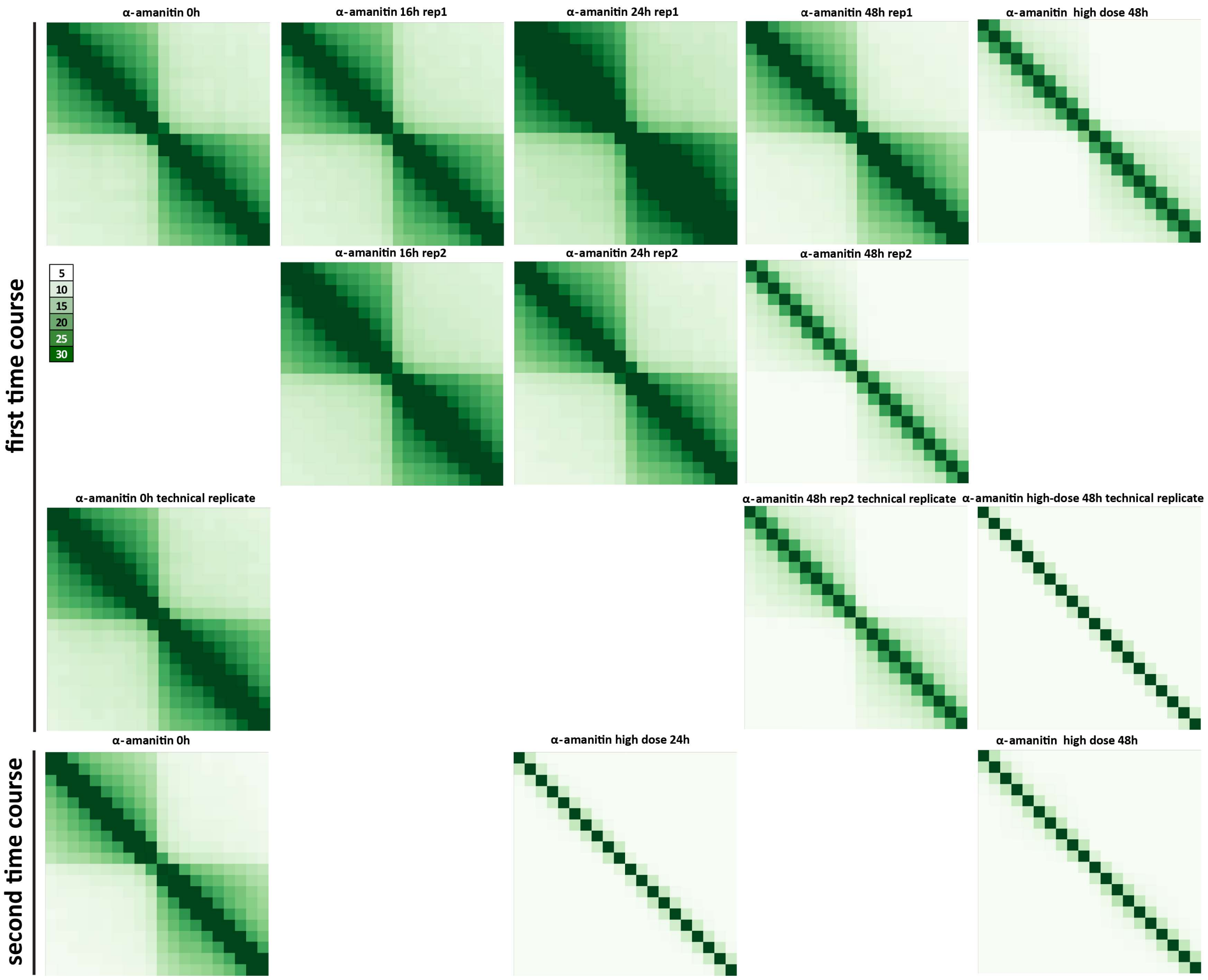

Supplementary Figure 25: Decompaction of dinoTADs upon transcriptional inhibition using α-amanitin. Shown are 50-kb resolution metaplots centered on dinoTAD domain boundaries. Two time courses were carried out following the outline presented in Figure 2B.

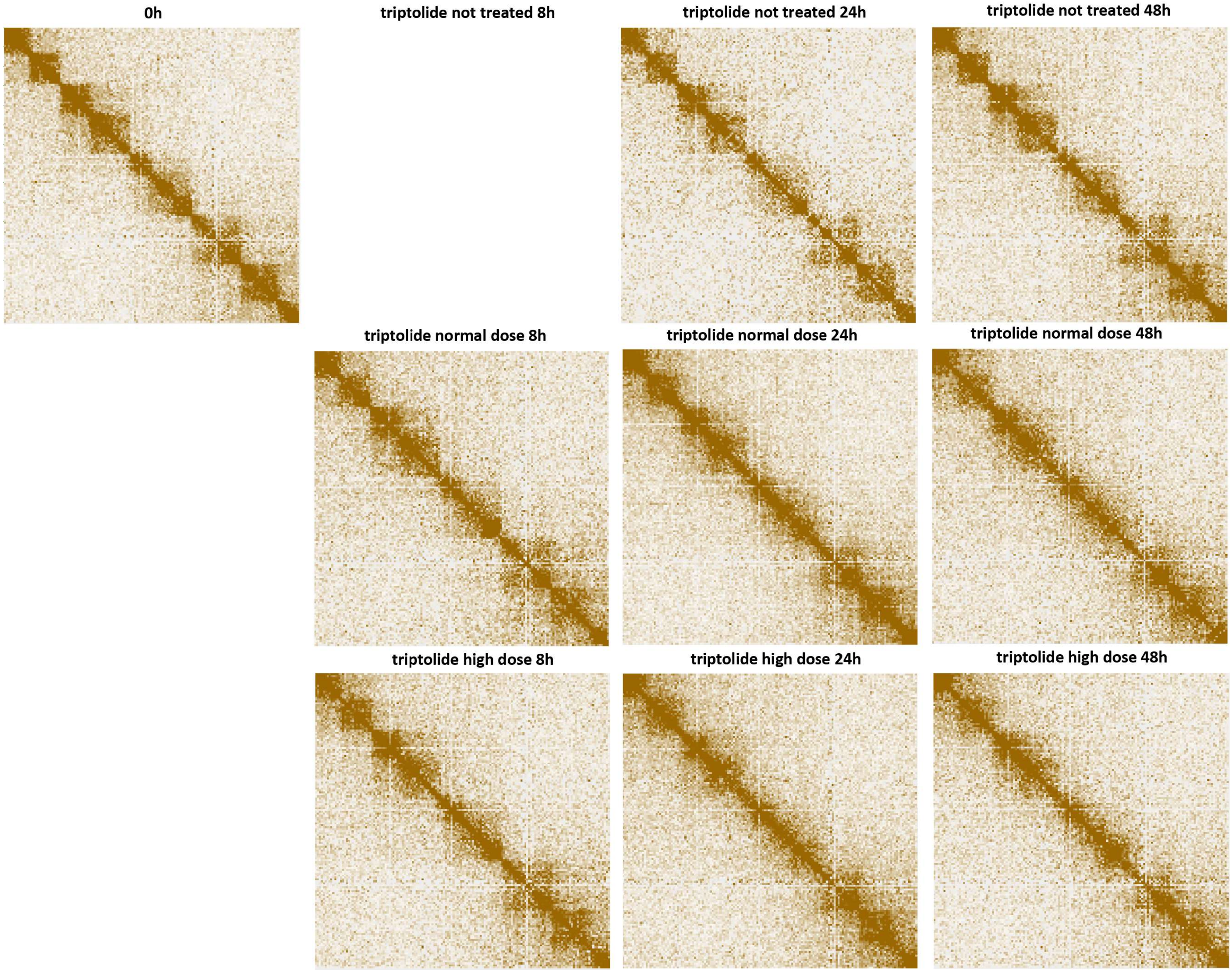

Supplementary Figure 26: Blurring of dinoTAD boundaries upon transcriptional inhibition using triptolide. Shown is pseudochromosome 10. The triptolide time course was carried out following the outline presented in Figure 2B.

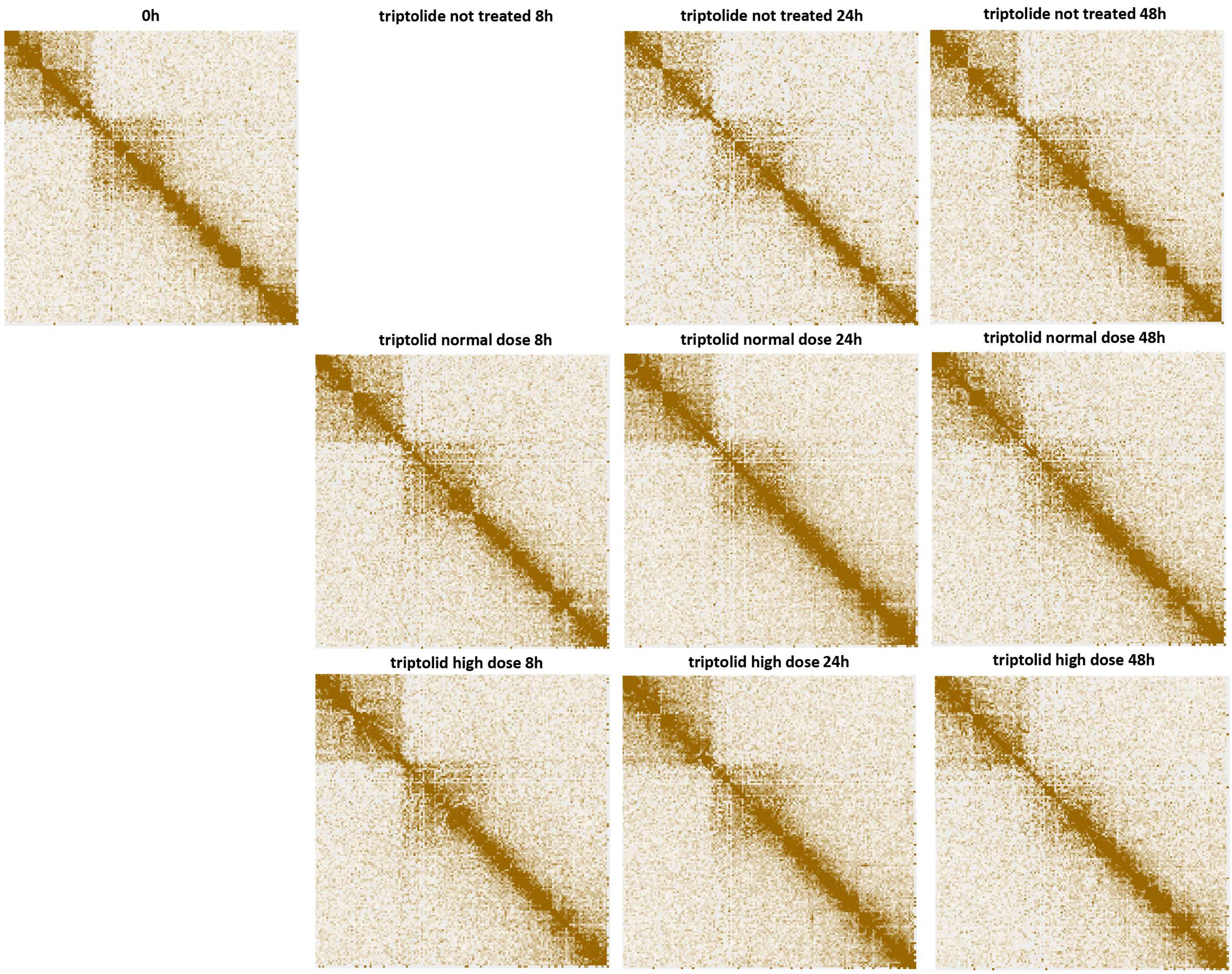

Supplementary Figure 27: Blurring of dinoTAD boundaries upon transcriptional inhibition using triptolide. Shown is pseudochromosome 17. The triptolide time course was carried out following the outline presented in Figure 2B.

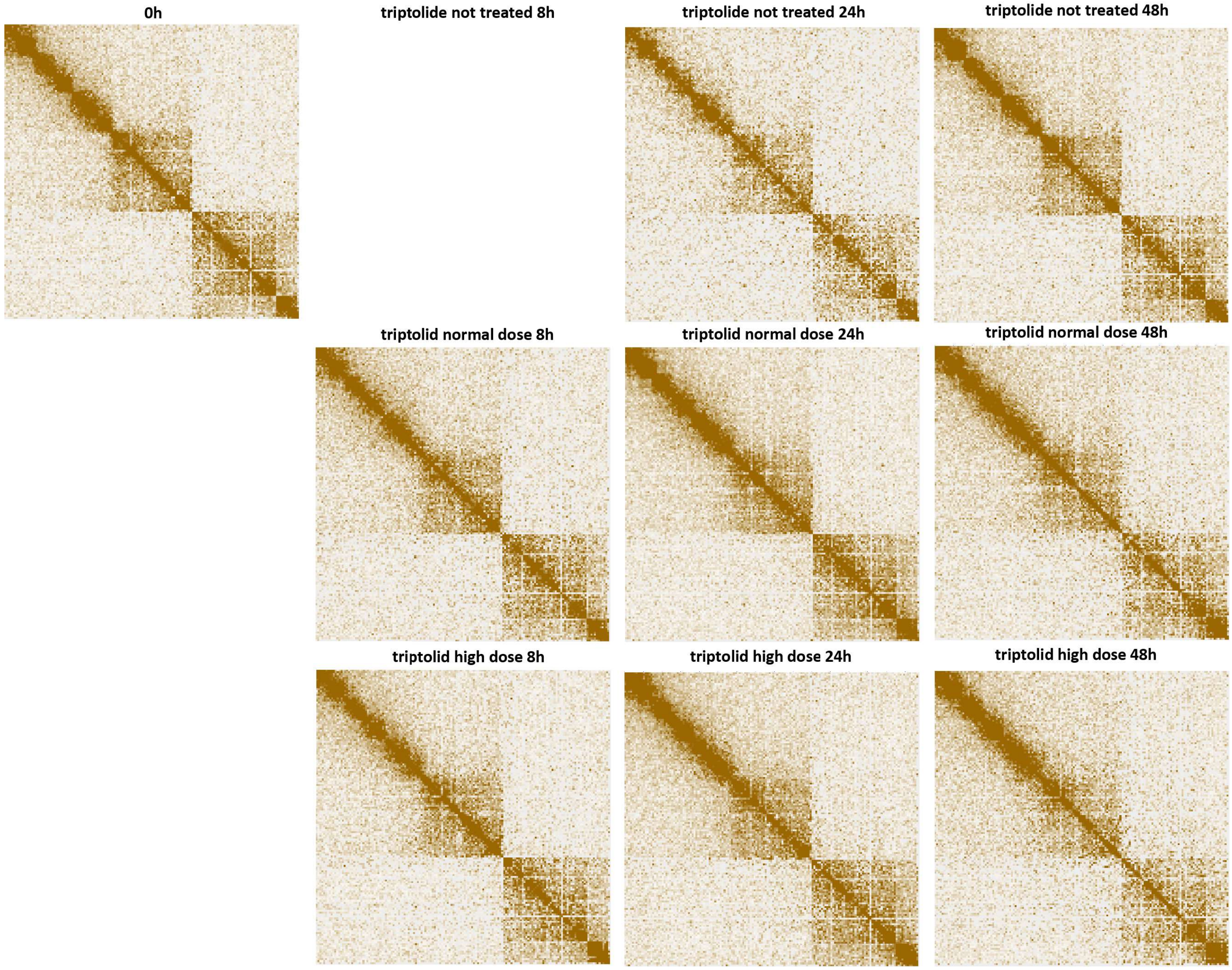

Supplementary Figure 28: Blurring of dinoTAD boundaries upon transcriptional inhibition using triptolide. Shown is pseudochromosome 18. The triptolide time course was carried out following the outline presented in Figure 2B.

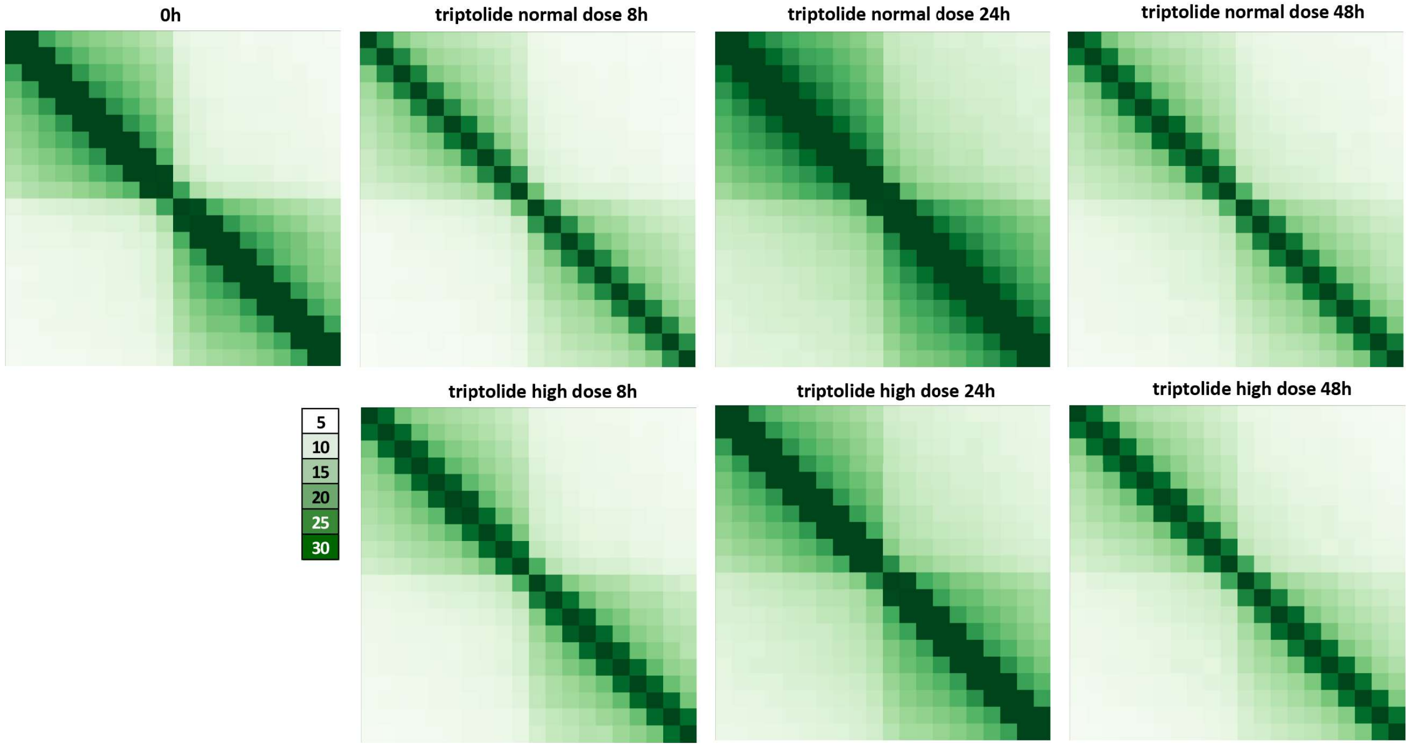

Supplementary Figure 29: Blurring of dinoTAD boundaries upon transcriptional inhibition using triptolide. Shown are 50-kb resolution metaplots centered on dinoTAD domain boundaries. The triptolide time course was carried out following the outline presented in Figure 2B.

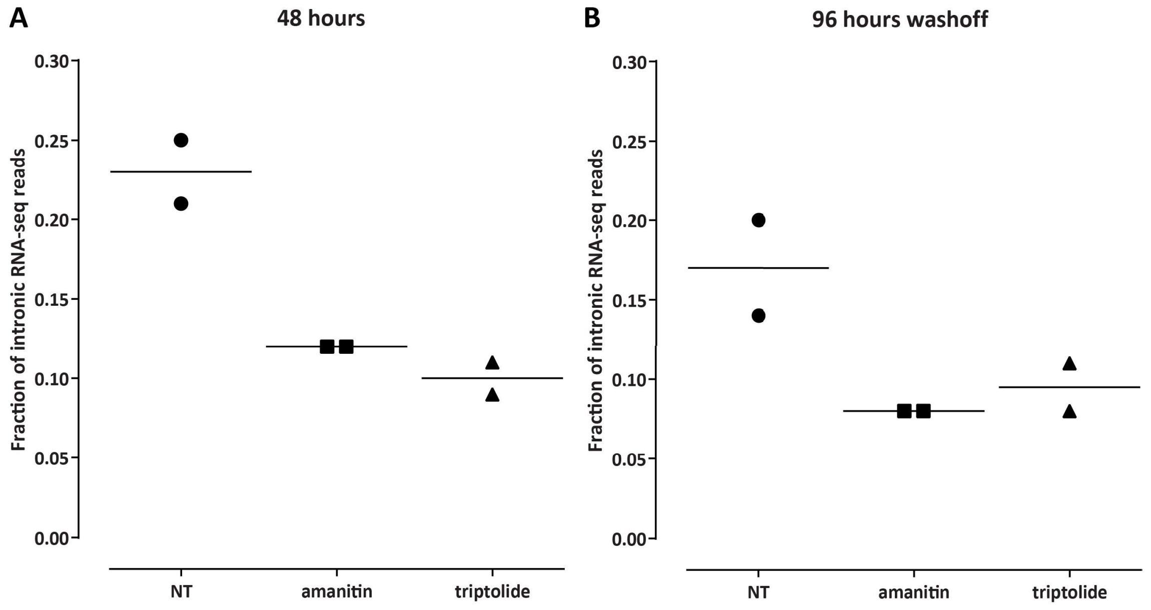

Supplementary Figure 30: Assessment of transcriptional activity upon α-amanitin and triptolide treatment and after withdrawal of the inhibitors. Shown is the fraction of intronic reads in PolyA+ RNA-seq datasets generated from cells treated with the "high" doses of the two drugs or no drug for 48 hours (A) , and at 48 hours later after withdrawing the inhibitor (B; "96 hours washoff"). Note that these samples correspond to the "third time course" shown in Supplementary Figures 32 and 33.

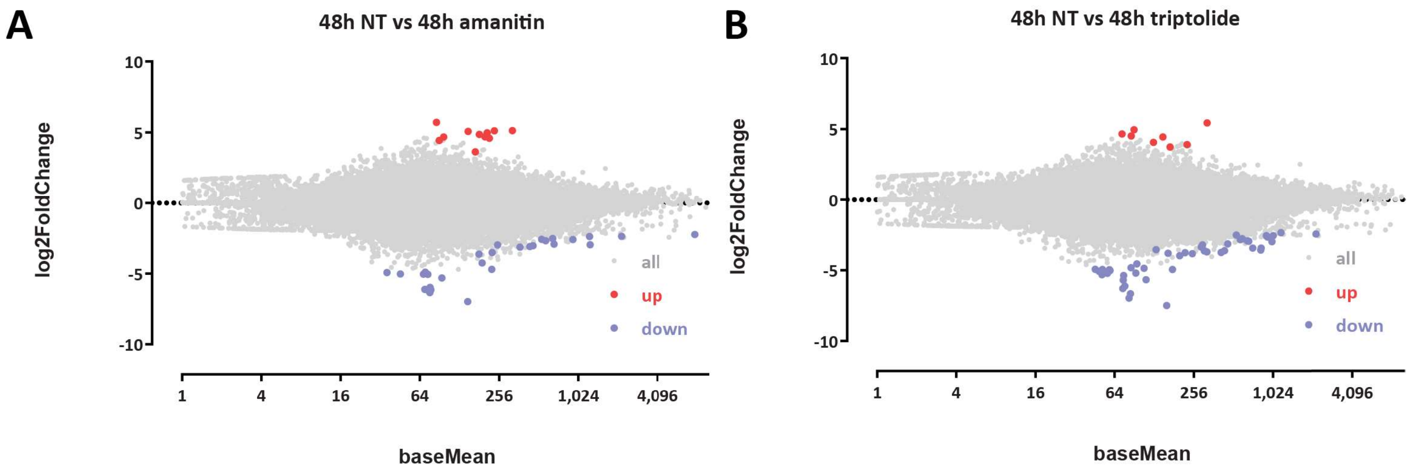

Supplementary Figure 31: Lack of large-scale transcript level changes upon α-amanitin and triptolide treatment. Differential expression was assessed using DESeq2 (see Methods). Number of differential genes: 12 genes up in and 30 genes down in the α-amanitin-treateed relative to the untreated sample; 9 genes up in and 47 genes down in the triptolide-treated relative to the untreated sample. Note that these samples correspond to the "third time course" shown in Supplementary Figures 32 and 33.

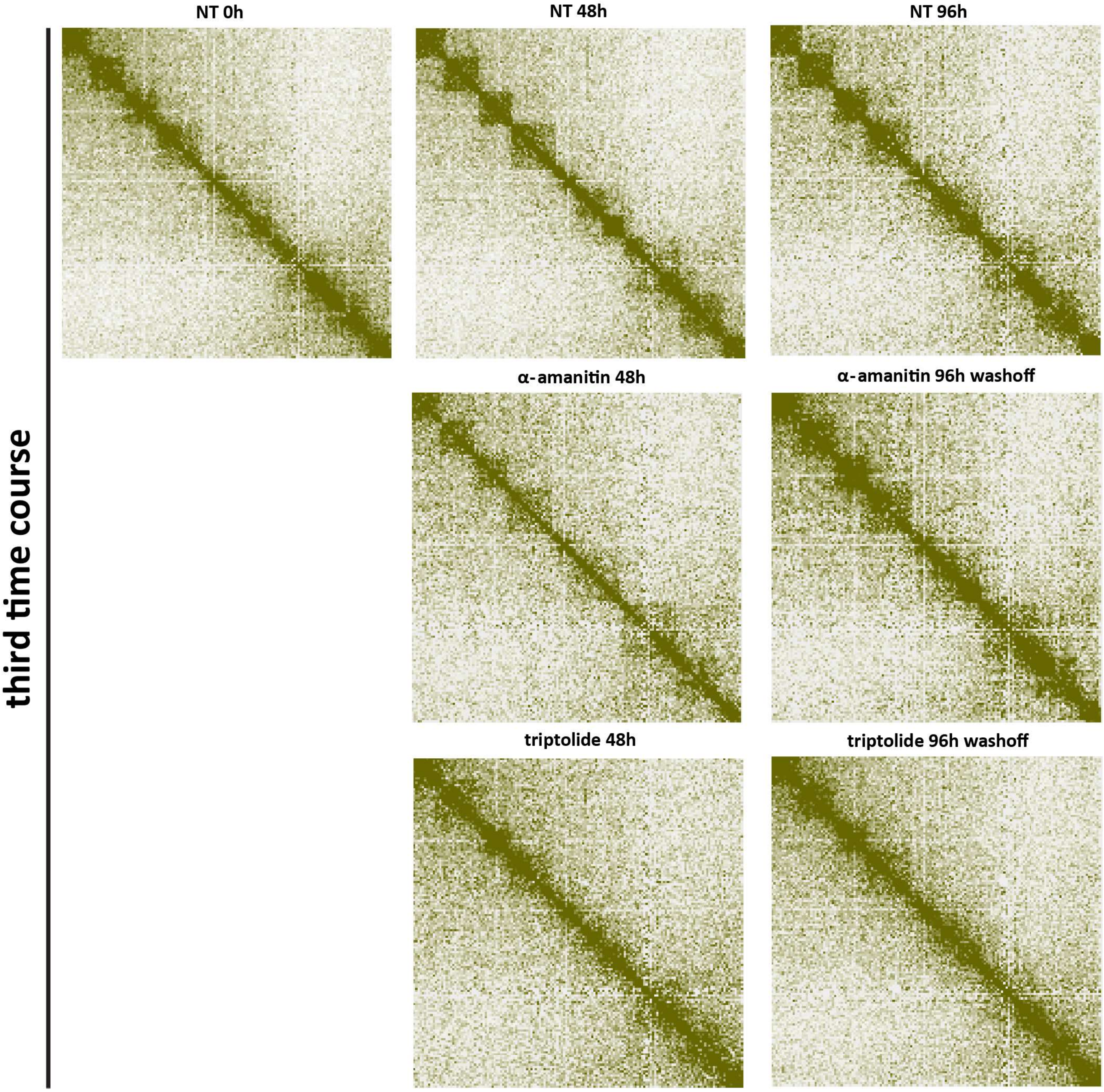

Supplementary Figure 32: Partial restoration of dinoTADs within 48 hours after removal of transcriptional inhibitors. Cells were treated with α-amanitin or triptolide ("high" doses) for 48 hours, then the inhibitors was washed away, and cells were harvested another 48 hours later ("96 hours washoff"). Shown is pseudochromosome 10.

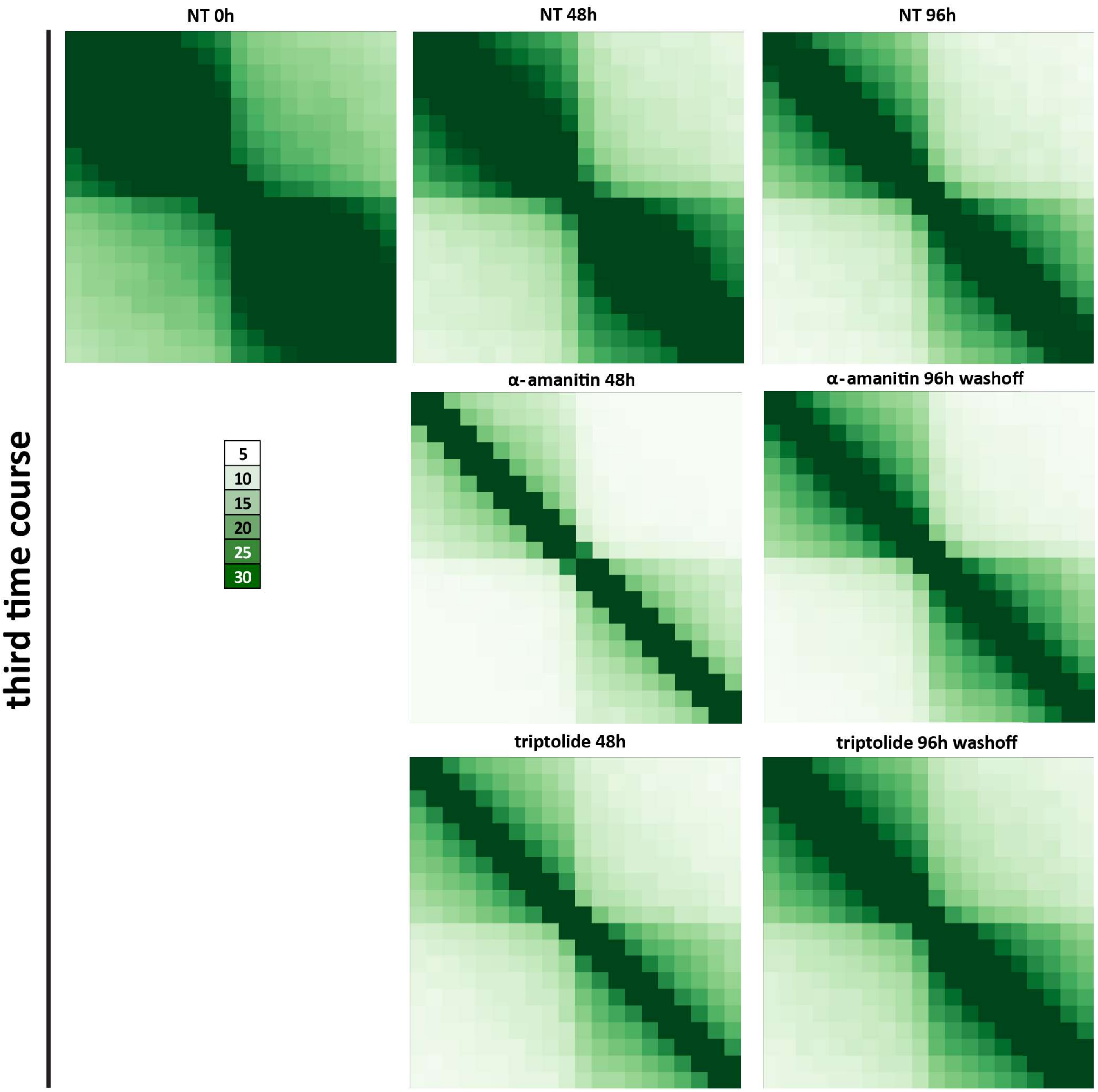

Supplementary Figure 33: Partial restoration of dinoTADs within 48 hours after removal of transcriptional inhibitors. Cells were treated with α-amanitin or triptolide ("high" doses) for 48 hours, then the inhibitors was washed away, and cells were harvested another 48 hours later ("96 hours washoff"). Shown is a metaplot across all dinoTAD boundaries.

35. Knight, P. & Ruiz, D. A fast algorithm for matrix balancing. *IMA J. Numer. Anal.* **33,** 1029–1047 (2013).
36. Hou, Y. et al. Genome size-dependent pcna gene copy number in dinoflagellates and molecular evidence of retroposition as a major evolutionary mechanism. *J. Phycol.* **55**, 37–46 (2019).
