# Supplementary material for: Transcription-dependent domain-scale three-dimensional genome organization in the dinoflagellate Breviolum minutum
Source: Nat Genet. 2021 Apr 29;53(5):613–7. doi: 10.1038/s41588-021-00848-5 (PMC8110477; doi:10.1038/s41588-021-00848-5)
Supplement: Supplementary file 3 — Peer Review Information [file 41588_2021_848_MOESM3_ESM.pdf]

Peer Review Information

Journal: Nature Genetics
Manuscript Title: Transcription-dependent domain-scale three-dimensional genome organization in the dinoflagellate *Breviolum minutum*
Corresponding author name(s): Dr. Georgi Marinov

Reviewer Comments & Decisions:

Decision Letter, initial version:

5th Aug 2020

Dear Georgi and Will,

Your Brief Communication, "Transcription-dependent domain-scale 3D genome organization in dinoflagellates" has now been seen by 3 referees. You will see from their comments copied below that while they find your work of considerable potential interest, they have raised quite substantial concerns that must be carefully addressed. In light of these comments, we cannot accept the manuscript for publication, but would be very interested in considering a revised version that addresses these serious concerns.

Reviewer #1 is quite positive about this work, in terms of resource value and biological novelty. Their comments are minor.
Reviewer #2 thinks the findings could be interesting but that the Hi-C data have low resolution (deeper sequencing is warranted) and that you should perform RNA-seq analysis. These requests seem doable.
Reviewer #3 says the work is interesting but preliminary. This reviewer is also worried about the quality and resolution of the Hi-C data and has suggested some additional experiments that are necessary to further strengthen the conclusions.

We hope you will find the referees' comments useful as you decide how to proceed. If you wish to submit a substantially revised manuscript, please bear in mind that we will be reluctant to approach the referees again in the absence of major revisions. You might want to also consider expanding the manuscript to a Letter format (https://www.nature.com/ng/about/content#letter).

If you choose to revise your manuscript taking into account all reviewer comments, please highlight all changes in the manuscript text file. At this stage we will need you to upload a copy of the manuscript

in MS Word .docx or similar editable format.

We are committed to providing a fair and constructive peer-review process. Do not hesitate to contact me if there are specific requests from the reviewers that you believe are technically impossible or unlikely to yield a meaningful outcome.

If revising your manuscript:

*1) Include a “Response to referees” document detailing, point-by-point, how you addressed each referee comment. If no action was taken to address a point, you must provide a compelling argument. This response will be sent back to the referees along with the revised manuscript.

*2) If you have not done so already please begin to revise your manuscript so that it conforms to our Brief Communication format instructions, available here.
Refer also to any guidelines provided in this letter.

*3) Include a revised version of any required Reporting Summary:
https://www.nature.com/documents/nr-reporting-summary.pdf
It will be available to referees (and, potentially, statisticians) to aid in their evaluation if the manuscript goes back for peer review.
A revised checklist is essential for re-review of the paper.

Please be aware of our guidelines on digital image standards.

You may use the link below to submit your revised manuscript and related files:

[REDACTED]

Note: This URL links to your confidential home page and associated information about manuscripts you may have submitted, or that you are reviewing for us. If you wish to forward this email to co-authors, please delete the link to your homepage.

Please do not hesitate to contact me if you have any questions or would like to discuss the required revisions further.

Nature Genetics is committed to improving transparency in authorship. As part of our efforts in this direction, we are now requesting that all authors identified as ‘corresponding author’ on published papers create and link their Open Researcher and Contributor Identifier (ORCID) with their account on the Manuscript Tracking System (MTS), prior to acceptance. ORCID helps the scientific community achieve unambiguous attribution of all scholarly contributions. You can create and link your ORCID from the home page of the MTS by clicking on ‘Modify my Springer Nature account’. For more information please visit please visit www.springernature.com/orcid.

Thank you for the opportunity to review your work.

Sincerely,

Tiago

Tiago Faial, PhD
Senior Editor
Nature Genetics
https://orcid.org/0000-0003-0864-1200

Referee expertise:

Referee #1: protist biology

Referee #2: 3D genomics

Referee #3: 3D genomics

Reviewers' Comments:

Reviewer #1:
Remarks to the Author:
In this manuscript, Marinov et al. used Hi-C to examine the three-dimensional organization of the Breviolum genome. The remarkable divergence of dinoflagellate chromosomes makes this a pertinent topic not only for understanding dinoflagellate biology but concepts in genome organization more broadly. The discovery of transcription-dependent topological domains containing convergent gene arrays is striking and the development of a chromosome scale assembly of the Breviolum genome will be an invaluable resource.
The manuscript was concise, well written, and well presented. The brevity of the article was appreciated but I think the text could be expanded for clarity and thoroughness in some instances. Nonetheless, my comments are minor and should be straightforward to address.
1. The mention of polycistronic arrays in the abstract and introduction may be misleading because there is little evidence for nuclear polycistronic mRNAs in dinoflagellates (see Beauchemin et al. 2012. PNAS 109:15793-15798). This could be replaced with “tandem gene arrays” to be more accurate.
2. It would be worthwhile to add a brief explanation of what Hi-C is at the start of paragraph four as this technique may not be widely known outside of genomics/chromatin fields.
3. For Figure 1A,B,C,F legends and bin sizes should be added (bin sizes could be added to the figure legend).
4. In Figure 1C, what does the dotted circle represent? It is not mentioned in the figure legend.
5. In Figure 1A and 1C, species names should be italicized.
6. In Figure 1G, a y-axis should be added. It’s a bit confusing that the positive values on the y-axis are above the negative values. Also for the legend, is TPM the correct unit here since the T refers to transcript and the legend indicates the plot is showing Hi-C contacts? A variation of this may be more appropriate (FPM?).

7. At the beginning of paragraph five, the topological domains are referred to as strong. What is meant by strong? I assume it is meant to reflect the well defined TAD boundaries/insulation but this should be clarified. It also may be worth citing Figure 1G here.
8. In its present form, the analysis of topoisomerases is not very convincing nor a robust point supporting the plectoneme model. The main issue is that, in general, dinoflagellates have expanded gene repertoires (reflected in their tandem gene arrays) so topoisomerase may not be a special case. This is even demonstrated in Supplemental Figure 17 where the majority of non-topoisomerase genes are also increased in dinoflagellates. To make this claim, it would be important to show that topoisomerases have expanded more relative to other genes, otherwise this could be an argument for almost anything. To do this, I imagine you would need to compare copy number for all orthologues between dinoflagellates and other eukaryotes and then inspect where topoisomerases fall relative to the others.
9. In paragraph 15, another likely rationalization for the differences between domain insulation in dinoflagellates compared the bacteria and yeast may be the structure of the chromosomes themselves (i.e., rod shaped instead of traditional interphase chromosomes). A more comparable system may be TADs in mitotic chromosomes.
10. It would be worthwhile to discuss previous observations of plectoneme like structures in dinoflagellate chromosome spreads (see Livolant and Bouligand 1980. Chromosoma. 80, 97-118). Models have also already been proposed describing a role for plectoneme domains in dinoflagellate chromosomes (see Wong 2019 Microorganisms 7, 27; doi:10.3390/microorganisms7020027). It would be interesting to discuss these models (which were not based on Hi-C data) and observations in the context of the new data. This would also further support the presence of plectonemes given that they don’t seem to be very apparent in the Hi-C heatmaps.
11. In paragraph 16, additional discussion on the role of dinoflagellate chromatin proteins, specifically DVNP and HLPs, in defining chromosome structure would be interesting. For example, the acquisition of HLPs coincides with the emergence of the unique liquid crystal chromosomes suggesting they may play a role in chromosome structure. Some in vitro work has been done on these proteins and this literature may be worth reviewing and discussing if relevant (possibly in boundary formation?) (see Chan and Wong, 2007, Nucleic Acids Research, 35:2573-2583).
12. In the supplemental methods, in the last sentence of Transcription inhibition experiments, “fat” should be “at”.
13. The supplemental methods seem to switch between Breviolum and Symbiodinium, this should be standardized throughout.

Reviewer #2:
Remarks to the Author:
The manuscript describes an analysis of the 3D organization of the B. minitum genome. This dinoflagellate is of special interest because its DNA is not packed into nucleosomes, making this organism an interesting system to study the mechanisms underlying the formation of contact domains. Authors conclude that supercoiling plays a critical role in the formation of contact domains in dinoflagellates.

The manuscript could be, in principle, interesting if the authors are able to derive general principles of 3D organization from the study of this organism. Major issues with the paper in its current form include the low resolution of the Hi-C data, which limits the ability of the authors to make meaningful conclusions, and the lack of RNA-seq data in the different conditions used to analyze the possible role

of transcription in 3D organization, which makes the results difficult to interpret. The following are specific issues that I hope will help the authors improve the significance and validity of the conclusions:

1. One critical issue in the interpretation of Hi-C data is the resolution. Since all analyses described in the manuscript involve intra-chromosomal interactions, authors should include additional columns in Table S1 indicating the fraction of intra- and inter-chromosomal contacts. Each Hi-C heatmap in the figures should indicate the resolution of the data, and scale bars should be included. The easiest way to do this would be to process the data using Juicer and to show plots from Juicebox. Each experiment should have biological, not technical, replicates and information on each replicate should be included. The number of contacts for some of the samples, as low as 15 million, is woefully inadequate. It is difficult to judge the resolution of the data because the size of the B. minitum genome to which the reads are being mapped (excluding repeated sequences) is not mentioned. Any description of the data as “high-resolution” should be deleted from the text unless it is really high-resolution. In mammals, Hi-C data from Rao et al Cell 2014 containing 4.5 billion contacts is the standard for high-resolution, which in this case is 1 kb.

2. Figure 1F. The overlap between the TAD boundaries and the location of divergent transcription is difficult to judge visually. In fact, from this figure, the two do not appear to overlap at many boundaries. Figures S3-13 show many domain boundaries that do not correspond sites of convergent transcription and many sites of convergent transcription that do not correspond to domain boundaries. This conclusion is an important aspect of the manuscript and cannot be reached based on visual observations only. It’s unclear whether Figure 1H really addresses this concern. Are all RNA-seq data plotted in this figure or on the data for the convergent genes at the boundaries? Shouldn’t transcript levels be zero at the boundary? Authors should map the location of the TAD boundaries and then analyze the overlap with the location of boundaries between divergent gene arrays. Please note that the ability to properly map the location of TAD boundaries will depend on the resolution of the data.

3. Figure 3C. Please include a distance scale and locations of divergent transcription in this panel. Visually, it appears that this “TAD” is composed of smaller domains that authors may be able to identify with higher-resolution Hi-C data. This is also obvious in supplementary figures 3-13.

4. Page 3, top left. The nomenclature used to name “contact domains” in the mammalian literature is confusing and following the mammalian convention for dinoflagellates is even more confusing. Here authors use “TAD” to designate CTCF loops in mammals and suggest a different mechanism for the formation of dinoTADs because these domains lack “corner dots” and extrusion “stripes”. It may not be appropriate to make this comparison. Mammalian cells depleted of CTCF lack CTCF loops but still have contact domains similar to the dinoTADs. Therefore, dinoTADs are likely the same as domains present in mammalian cells different from CTCF loops. Please see Rao et al Cell 2017 for a discussion of the nature of these domains.

5. If I understand correctly, the treatments with HS or Pol II inhibitors was not followed by RNA-seq experiments. It may be important to know how much transcription is affected in each condition in order to properly interpret the Hi-C differences. This is especially important because, unless the authors know this, it is not known whether B. minitum undergoes a heat shock response at the temperatures used and whether triptolide and a-amanitin can get inside of the cells or affect Pol II. For example, a-amanitin does not affect yeast cells because they are not permeable to this compound. Please cite the appropriate references if this is known. One additional concern is the long treatment

times used here, which could affect transcription of proteins necessary for interactions detected by Hi-C and thus the observed effects could be indirect.

6. It is unclear what results led the authors to conclude that supercoiling must be involved in the formation of contact domains in B. minitum. Although the authors name the contact domains observed in Hi-C as “TADs”, there is no evidence that these domains are “topological” in any organism. The positive/negative supercoiling generated during transcription is released as Pol II advances and it does not accumulate at the 3’ of genes. Domains observed in Hi-C data are composed of interactions mediated by proteins that are sufficiently closed to be crosslinked by formaldehyde, which is a short-range (2-3 A) cross-linker. Therefore, proteins mediating interactions in Hi-C are probably interacting biochemically with each other. A contact domain is formed because of changes in the directionality of interactions, which is probably determined by the nature of the proteins present in different regions of the genome rather than by some physical force caused by supercoiling. In other organisms there is no correlation between the location of supercoils determined by psoralen experiments and location of domain boundaries. In addition to inhibiting transcription, triptolide and a-amanitin result in degradation of Poll II, which may also affect the recruitment of other components of the transcription process. Performing ChIP-seq with antibodies to Pol II phosphorylated in Ser2 or ser5, if the polymerase of B. minitum has CTDs, may be a way to explore this question in more detail. In yeast cohesin accumulates at the 3’ ends of genes as a consequence of transcription. If this happens in B. minitum and cohesin makes a loop between the 5’ and 3’ while it happens (some evidence for this is presented in Zheng et al Nature 2019 PMID 30778195), it may explain the formation of domains in dinoflagellates. This possibility is also supported by results in Drosophila where single DpnII fragment resolution (250 bp on average) Hi-C data suggest the formation of loops between the 5’ and 3’ of individual genes. Higher resolution Hi-C maps for B. minitum may allow the authors to map subdomains within the dinoTADs and analyze their correlation with genomic and transcription feature. A more accurate map of boundaries may allow the authors to gain insights into these different possibilities.

7. I wonder if the authors could learn something about mechanisms of 3D organization in B. minitum by comparing their data to that of S. cerevisiae, where genes are also closely packed. Do domain boundaries in cerevisiae form at each gene boundary, independent of the orientation, or only at sites of convergent genes?

Reviewer #3:
Remarks to the Author:
In eukaryotes, genome folding is known to be driven by cohesin-mediated loop extrusion, together with CTCF in vertebrates, to establish topologically associating domains (TADs). The role of transcription in genome folding remains elusive. Besides, replication has also been linked to the establishment of genome structure.

In this paper, Marinov, Trevino, Xiang and colleagues carry out Hi-C analysis on a species of dinoflagellate, Breviolum minutem, generating 91 major pseudochromosomes, and reveal dinoTADs, appearing as squares on heatmaps, similar to TADs in metazoan genomes. But these dinoTADs are much larger than mammalian TADs. Each dinoTAD corresponds to a pair of divergent gene arrays with a boundary coinciding with convergence between gene blocks.

After transcription inhibition by different methods, these dinoTADs are weakened to different extents. The primary conclusion of this paper is that transcription-induced supercoiling is the primary force driving dinoTAD formation. Though it is plausible, there are a few concerns that require further clarification from the authors.

Major comments:

1. The quality of Hi-C experiments may cause unexpected differences in heatmaps, and sometimes may complicate the interpretation. In Supplementary Table 1, the percentage of the Hi-C contacts over the raw read pairs differs for different experimental conditions. For control Hi-C, 27 and 34 degree cultures, the average percentage of Hi-C contact is 33% (27% to 41%). For samples treated with low concentration of alpha amanitin, the average percentage is 31% (26 to 40%), similar to control. Under this condition, the dinoTADs are not obviously weakened to my eyes (Supplementary Figures 21 to 23). However, when the alpha amanitin concentration is increased, the average Hi-C contact percentage drops to averagely 22% (17% to 27%), which, I think, is significantly lower than Hi-C of controls, heat shocked, and low alpha amanitin concentration treated conditions. For triptolide-treated samples, the average percentage of Hi-C contacts is 29% (25% to 33%), similar to Hi-C of controls, heat shocked and low alpha aminitin concentration treated conditions. And accordingly, the dinoTADs seem to be better maintained. Whether the percentage of Hi-C contacts in raw total paired reads can be used as an indicator of Hi-C quality can be debated. However, the correlation of low Hi-C contact percentage and weakened dinoTAD partition may not totally be a coincidence.

2. If transcription inhibition does degrade dinoTADs (Figure 2), we may well expect that after the removal of the inhibiting drugs, transcription will resume and dinoTADs will reappear. The authors may consider to do some Hi-C on cells recovering from transcription inhibition and to show if dinoTADs will reemerge.

3. In Figure 2H, topological domain formation is proposed to be established by transcription. If unidirectional genes are in a block twisted together and separated from genes of different orientation in the adjacent block, which is still in the same dinoTAD, we would expect to see two “sub-TADs” within a large dinoTAD. Actually, if we examine the Hi-C heatmap plotted at 5kb, indeed, sub-TADs corresponding to single unidirectional gene blocks can be observed (Supplementary Figures 3-13). The authors may reexamine the heatmaps of Hi-C at high resolution (5kb) to reveal to what extent these substructures exist in the genome.

4. Following point #3, florescence in situ hybridization (FISH) may be carried out to show if genes within the same block are closer than genes in adjacent blocks that are in the same dinoTAD.

5. Another force that the authors overlooked is replication. How long is the cell cycle? The time length of transcription inhibition may just be enough to reduce gene expression to a level that arrests cells at a specific phase of cell cycle. The authors may need to consider inhibiting replication as well to see if the dinoTADs will be weakened.

Minor comments:

1. Convergent gene transcription at borders was reported in Drosophila genome by Hou and colleagues in 2012 (Mol. Cell). Despite the huge differences between the genomes of insects and dinoflagellates, it would be interesting to discuss if convergent genes frequently found at boundaries

could be evolutionarily conserved.

2. Could the authors use any metrics to quantitatively measure the differences, for example, in the border separation, or directionality index, or the dinoTADs internal strength?

3. Color bars are missing for nearly all Hi-C heatmaps in both Figures and supplementary materials.

Overall, this is an interesting work providing evidence for the role of transcription in driving the establishment of dinoTADs. However, as stated above, I think at this stage, additional evidence is required to support this claim.

Author Rebuttal to Initial comments

Transcription-dependent domain-scale 3D genome organization in dinoflagellates. Response to reviewer comments

Reviewer 1:

In this manuscript, Marinov et al. used Hi-C to examine the three-dimensional organization of the Breviolum genome. The remarkable divergence of dinoflagellate chromosomes makes this a pertinent topic not only for understanding dinoflagellate biology but concepts in genome organization more broadly. The discovery of transcription-dependent topological domains containing convergent gene arrays is striking and the development of a chromosome scale assembly of the Breviolum genome will be an invaluable resource.

The manuscript was concise, well written, and well presented. The brevity of the article was appreciated but I think the text could be expanded for clarity and thoroughness in some instances. Nonetheless, my comments are minor and should be straightforward to address.

We thank the reviewer for the thoughtful comments.

1. The mention of polycistronic arrays in the abstract and introduction may be misleading because there is little evidence for nuclear polycistronic mRNAs in dinoflagellates (see Beauchemin et al. 2012. PNAS 109:15793-15798). This could be replaced with "tandem gene arrays" to be more accurate.

We thank the reviewer for the comment. We have replaced the term "polycistronic gene arrays" with "tandem gene arrays" throughout the manuscript, though we would like to point out that dinoflagellate

transcripts are certainly *trans*-spliced (even if that can happen without them being polycistronic) and that there is little evidence for independent promoter elements for each gene in an array. This is a subject we expect to see dissected in detail in future studies.

2. It would be worthwhile to add a brief explanation of what Hi-C is at the start of paragraph four as this technique may not be widely known outside of genomics/chromatin fields.

We have added such a clarification to the text.

3. For Figure 1A,B,C,F legends and bin sizes should be added (bin sizes could be added to the figure legend).

We are unsure as to what the reviewer is referring to as the bin size (5-kb) is indeed already specified in the figure legend. We now also mention it in the text.

4. In Figure 1C, what does the dotted circle represent? It is not mentioned in the figure legend.

We have added a clarification in the figure legend.

5. In Figure 1A and 1C, species names should be italicized.

We have corrected this.

6. In Figure 1G, a y-axis should be added. It's a bit confusing that the positive values on the y-axis are above the negative values. Also for the legend, is TPM the correct unit here since the T refers to transcript and the legend indicates the plot is showing Hi-C contacts? A variation of this may be more appropriate (FPM?).

Hi-C maps are generally understood to be 2D matrices with the same axes on the x and the y. We had not added a second labeled axis in order to avoid cluttering the figure with unnecessary text.

Regarding TPMs (we assume the reviewer is referring to 1H, not 1G in that case): the RNA-seq datasets we worked with were not strand-specific, thus simple alignment and calculation of stranded tracks in RPM/FPM units is not possible. As explained in the Methods section, we instead quantified newly assembled (in genome space) transcripts, which are stranded thanks to the presence of splice junctions, then used the TPM units of the transcript quantification to assign strand-specific expression values for each exon of each transcript.

7. At the beginning of paragraph five, the topological domains are referred to as strong. What is meant by strong? I assume it is meant to reflect the well defined TAD boundaries/insulation but this should be clarified. It also may be worth citing Figure 1G here.

They are "strong" in the sense that there are few contacts between them and a lot of contacts within them, comparable to TADs in mammals. This domain strength is not seen in other species where

supercoiling domains have been observed. The comparison with *S. pombe* and *Caulobacter* that we provide in the supplement (Supplementary Figures S15 and S16) visualizes the difference. We have added a clarification in the text to better explain that point.

8. In its present form, the analysis of topoisomerases is not very convincing nor a robust point supporting the plectoneme model. The main issue is that, in general, dinoflagellates have expanded gene repertoires (reflected in their tandem gene arrays) so topoisomerase may not be a special case. This is even demonstrated in Supplemental Figure 17 where the majority of non-topoisomerase genes are also increased in dinoflagellates. To make this claim, it would be important to show that topoisomerases have expanded more relative to other genes, otherwise this could be an argument for almost anything. To do this, I imagine you would need to compare copy number for all orthologues between dinoflagellates and other eukaryotes and then inspect where topoisomerases fall relative to the others.

Dinoflagellates indeed have frequently expanded gene families, but they have neither universally nor uniformly expanded gene repertoires. See for example Marinov & Lynch 2015[1] for an analysis of chromatin-related genes. The analysis in the supplement (Supplementary Figure 18) demonstrates that too (for example, RPA2 and RPA3 are not expanded), as does the analysis in the main text (Figure 2H) – TOP1 topoisomerases are not expanded, while TOP2 genes are greatly amplified, and TOP3 genes are somewhat amplified. Furthermore, expansion is specifically observed for TOP2 and not TOP1 topoisomerase, and it is TOP2 topoisomerases that are expected to deal with transcription-induced supercoiling.

To further clarify this point, we analyzed the number of genes containing a given PFAM domains in the *B. minutum* genome as a means to measure the distribution of gene amplifications (Response To Reviewers Figure 1). This analysis demonstrates that gene family amplifications are highly non-uniform, and that most gene families are in fact not amplified.

We include analysis of PCNA and MCM genes in the supplement because there are in fact putative reasons why those genes are specifically amplified in dinoflagellates that were previously discussed in the literature (see PMID: 30468510 for more details); we have now added a reference to make that connection more explicit.

9. In paragraph 15, another likely rationalization for the differences between domain insulation in dinoflagellates compared the bacteria and yeast may be the structure of the chromosomes themselves (i.e., rod shaped instead of traditional interphase chromosomes). A more comparable system may be TADs in mitotic chromosomes.

The relationship between the large-scale shape of dinoflagellate chromosomes and the local domains seen in Hi-C maps is at present difficult to establish. One possibility is that the coil-like structures within the rods that are observed in high-resolution EM images correspond to the domains seen in Hi-C; this

[1] Marinov GK, Lynch M. 2015. Diversity and Divergence of Dinoflagellate Histone Proteins. *G3 (Bethesda)* **6**(2):397–422.

would imply one-to-one correspondence between the number and identity of dinoTADs and the number and identity of coil-like structures. However, we suspect that in reality the picture is much more complicated, as existing EM images of *Symbiodinium* cells show examples of rods with many more coils than the number of domains in any of the available pseudochromosomes. It is hard to envisage a possible mechanism through which global compaction into rods could lead to the local domains seen in Hi-C. We hope techniques like superresolution imaging could resolve these questions in the future, but this is beyond the scope of the current manuscript.

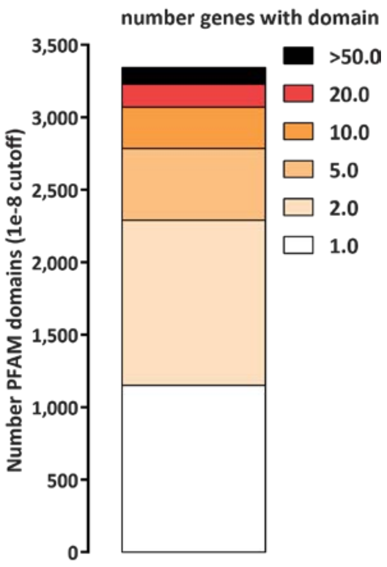

Response to Reviewers Figure 1: Dinoflagellate genes are not uniformly massively expanded and such a general non-specific expansion does not explain the observed TOP2 amplification. Shown is the number of genes per PFAM domain (at a cutoff of 1e-8) for the *B. minitum* genome. A majority of domains are found in only one or two genes.

10. It would be worthwhile to discuss previous observations of plectoneme like structures in dinoflagellate chromosome spreads (see Livolant and Bouligand 1980. Chromosoma. 80, 97-118). Models have also already been proposed describing a role for plectoneme domains in dinoflagellate chromosomes (see Wong 2019 Microorganisms 7, 27; doi:10.3390/microorganisms7020027). It would be interesting to discuss these models (which were not based on Hi-C data) and observations in the context of the new data. This would also further support the presence of plectonemes given that they don’t seem to be very apparent in the Hi-C heatmaps.

We thank the reviewer for the suggestions. We had in fact included a section on previous models of dinoflagellate chromosomes, but eliminated it from the original submission due to length constraints. We have now reincorporated these references and discussion into the text.

11. In paragraph 16, additional discussion on the role of dinoflagellate chromatin proteins, specifically DVNP and HLPs, in defining chromosome structure would be interesting. For example, the acquisition of HLPs coincides with the emergence of the unique liquid crystal chromosomes suggesting they may play a role in chromosome structure. Some in vitro work has been done on these proteins and this literature may be worth reviewing and discussing if relevant (possibly in boundary formation?) (see Chan and Wong, 2007, Nucleic Acids Research, 35:2573-2583).

We thank the reviewer for the suggestion. In our view it is more likely that the histone proteins that dinoflagellates still retain, although in a very divergent form, are involved in setting boundaries rather than the DVNPs and the HLPs; this would fit better with the fact that histones are not very abundant (and thus are more likely to associate with specific regions of the genome) while the DVNPs are the main packaging component. Although this is speculation, such a putative role is also supported by the evidence that histones play an inhibitory role on plectoneme formation, as suggested by Hi-C data from dinoflagellates and kinetoplastids. These questions remain to be resolved by future studies that directly map histone occupancy over dinoflagellate genomes; this is at present not possible due to the lack of antibodies that recognize *Symbiodinium* histones, and due to the great difficulty of successfully introducing epitope-tagged transgenes into dinoflagellate cells.

12. In the supplemental methods, in the last sentence of Transcription inhibition experiments, “fat” should be “at”.

We thank the reviewer for spotting this typo. We have corrected it.

13. The supplemental methods seem to switch between Breviolum and Symbiodinium, this should be standardized throughout.

We thank the reviewer for pointing this out. We have standardized the species name to *B. minutum* throughout the supplement.

Reviewer 2:

The manuscript describes an analysis of the 3D organization of the B. minitum genome. This dinoflagellate is of special interest because its DNA is not packed into nucleosomes, making this organism an interesting system to study the mechanisms underlying the formation of contact domains. Authors conclude that supercoiling plays a critical role in the formation of contact domains in dinoflagellates.

The manuscript could be, in principle, interesting if the authors are able to derive general principles of 3D organization from the study of this organism. Major issues with the paper in its current form include the low resolution of the Hi-C data, which limits the ability of the authors to make meaningful conclusions, and the lack of RNA-seq data in the different conditions used to analyze the possible role of transcription in 3D organization, which makes the results difficult to interpret. The following are specific issues that I hope will help the authors improve the significance and validity of the conclusions: We thank the reviewer for the comments and suggestions.

1. One critical issue in the interpretation of Hi-C data is the resolution. Since all analyses described in the manuscript involve intra-chromosomal interactions, authors should include additional columns in Table S1 indicating the fraction of intra- and inter-chromosomal contacts. Each Hi-C heatmap in the figures should indicate the resolution of the data, and scale bars should be included. The easiest way to do this would be to process the data using Juicer and to show plots from Juicebox. Each experiment should have biological, not technical, replicates and information on each replicate should be included. The number of contacts for some of the samples, as low as 15 million, is woefully inadequate. It is difficult to judge the resolution of the data because the size of the B. minitum genome to which the reads are being mapped (excluding repeated sequences) is not mentioned. Any description of the data as high-resolution should be deleted from the text unless it is really high-resolution. In mammals, Hi-C data from Rao et al Cell 2014 containing 4.5 billion contacts is the standard for high-resolution, which in this case is 1 kb.

We are afraid that there is some confusion regarding the resolution of our Hi-C data sets. For the purposes of scaffolding the genome and also for making the claim that loops are not observed, reads were pooled across the initial set of libraries (from samples not treated with transcription inhibitors), resulting in resolution of 5-kbp. The density of reads in a HiC matrix scales as slightly slower than the square of the genome size. Because the *B. miniutum* assembly is approximately 1/5th the size of the human haploid genome (~613Mbp vs ~3,000 Mbp; we have now changed Figure 1 to show the assembly size, and we apologize for this omission), we need far fewer contacts (perhaps 1/10th) than the 4.5 billion in Rao et al. We comfortably exceed that threshold, with ≥550M contacts.

Furthermore, the presence of loop and stripe features is readily apparent in an unrelated Hi-C dataset that we have generated for a vertebrate organism with a genome size of 1.2 Gbp, for which only 75M contacts were obtained (Response To Reviewers Figure 2). Therefore we are confident these features do not exist to the degree they do in other vertebrate organisms.

When low-depth sequencing datasets were used, they were only used for the analysis of very large-scale features, for which their depth is sufficient. To further justify this decision, we downsampled libraries to a uniform depth, and found that our conclusions were unchanged (see Response To Reviewers Figure 4 further below). This was in fact an analysis carried out prior to submitting the original manuscript.

Our results are also concordant with those of the related paper by Nand et al.[2], which mapped 3D organization in a different Symbiodiniaceae species, *Symbiodinium microadriaticum*.

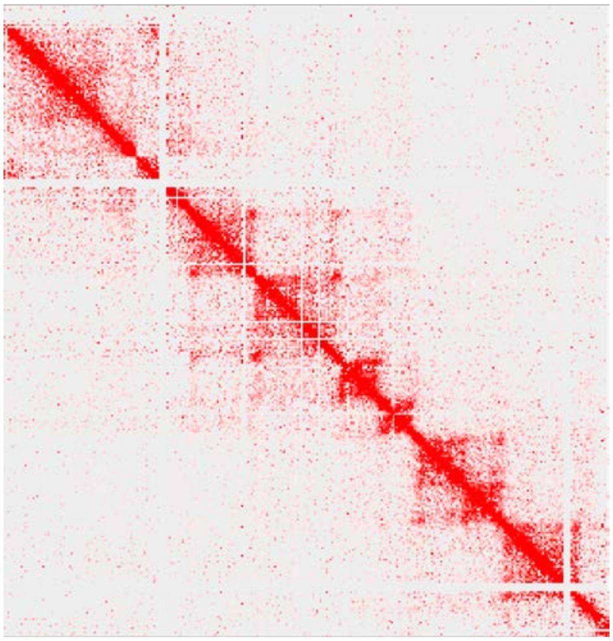

Response to Reviewers Figure 2: Sufficiency of the Hi-C coverage obtained in this manuscript for the detection of loop and stripe features. Shown is a Hi-C map for a vertebrate genome whose size is ~1.2 Gbp, and for which only 75M Hi-C contacts were obtained. Loop and stripe features are readily apparent. In contrast, the analysis presented in the main text is based on ≥550M contacts for a ~613 Mbp genome assembly.

2. Figure 1F. The overlap between the TAD boundaries and the location of divergent transcription is difficult to judge visually. In fact, from this figure, the two do not appear to overlap at many boundaries. Figures S3-13 show many domain boundaries that do not correspond sites of convergent transcription and many sites of convergent transcription that do not correspond to domain boundaries. This conclusion is an important aspect of the manuscript and cannot be reached based on visual observations only. It's unclear whether Figure 1H really addresses this concern. Are all RNA-seq data plotted in this figure or on the data for the convergent genes at the boundaries? Shouldn't transcript levels be zero at the boundary? Authors should map the location of the TAD boundaries and then analyze the

[2] Chromosome-scale assembly of the coral endosymbiont *Symbiodinium microadriaticum* genome provides insight into the unique biology of dinoflagellate chromosomes. Ankita Nand, Ye Zhan, Octavio R. Salazar, Manuel Aranda, Christian R. Voolstra, Job Dekker. *bioRxiv* 2020.07.01.182477

overlap with the location of boundaries between divergent gene arrays. Please note that the ability to properly map the location of TAD boundaries will depend on the resolution of the data.

We are confused by why the reviewer thinks that TAD boundaries do not overlap with the location of divergent transcription. There are cases where domains have a more complex structure, i.e. a rather large more diffuse domain includes several smaller weaker domains, which do consist of pairs of divergent arrays (the middle part of Supplementary Figure 4 features a prominent such example). We included those examples in the supplement to better represent the diversity of features observed in the data.

Because Symbiodiniaceae gene arrays are much longer than the Hi-C resolution we are working with, we do not expect that the resolution is problematic with regards to our overall conclusion.

As the reviewer points out, visual examples, though helpful, are insufficient to capture a particular architecture genome-wide. For this reason, we included 1H. However, RNA-seq data is not expected to be zero across TAD boundaries, as the boundaries between transcription units are much finer than the resolution at which TADs are defined by TAD-calling algorithms. This is why we observe some “bleedthrough” on both sides across TAD boundaries in the metaplots. To avoid this confusion, we could remove the points ~±20 kbp around the slightly “fuzzy” tad boundary to avoid this confusion – we are happy to do this at the editor’s discretion.

3. Figure 3C. Please include a distance scale and locations of divergent transcription in this panel. Visually, it appears that this TAD is composed of smaller domains that authors may be able to identify with higher-resolution Hi-C data. This is also obvious in supplementary figures 3-13.

We assume the reviewer is actually referring to Figure 1C (there is no Figure 3 in the main text). The scale is in fact already included, as Figure 1C is the inset from Figure 1B. The divergent units are also included, as Figure 1F features the exact same pseudochromosome as Figure 1C, which is also stated in the figure legend.

4. Page 3, top left. The nomenclature used to name “contact domains” in the mammalian literature is confusing and following the mammalian convention for dinoflagellates is even more confusing. Here authors use TAD to designate CTCF loops in mammals and suggest a different mechanism for the formation of dinoTADs because these domains lack “corner dots” and extrusion “stripes”. It may not be appropriate to make this comparison. Mammalian cells depleted of CTCF lack CTCF loops but still have contact domains similar to the dinoTADs. Therefore, dinoTADs are likely the same as domains present in mammalian cells different from CTCF loops. Please see Rao et al Cell 2017 for a discussion of the nature of these domains.

We thank the reviewer for initiating this discussion. However, we do indeed think that dinoTAD represent a distinct class of features than contact domains in mammals, thus leading us to define a new

term to separate them from what has previously been observed. This was done in order to avoid further confusion. Below, we discuss some of the distinctions.

It is true that compartments are preserved after CTCF depletion, and that some contact domain structure remains (Nora et al. 2017[34]). However, those contact domains are not suggested to coincide with divergent transcriptional units the way they do in dinoflagellates. Instead, they are hypothesized to arise from the preferential association of similarly modified nucleosomes.

Moreover, if the loss of histones in dinoflagellates had nothing to do with the appearance of dinoTADs in Hi-C maps, the expectation would be that the same features would be observed in kinetoplastids, which do possess the same kind of long divergent gene arrays but have retained a conventional set of histones. However, that is not the case – in fact, the available *Trypanosoma* Hi-C maps show no domain structure at all. It is also not the case that A/B compartments are observed in dinoflagellates. Thus, we think that for the time being it is best to refer to dinoflagellate domains with a distinct term.

5. If I understand correctly, the treatments with HS or Pol II inhibitors was not followed by RNA-seq experiments. It may be important to know how much transcription is affected in each condition in order to properly interpret the Hi-C differences. This is especially important because, unless the authors know this, it is not known whether B. minitum undergoes a heat shock response at the temperatures used and whether triptolide and a-amanitin can get inside of the cells or affect Pol II. For example, a-amanitin does not affect yeast cells because they are not permeable to this compound. Please cite the appropriate references if this is known. One additional concern is the long treatment times used here, which could affect transcription of proteins necessary for interactions detected by Hi-C and thus the observed effects could be indirect.

We fully agree with the reviewer that it is important to show that transcription was indeed inhibited. We would like, however, to point out that it is highly unlikely that α-amanitin and triptolide are not taken up by *Symbiodinium* cells – if indeed these cells were not permeable to them, we expect to see no difference between untreated and treated cells in our chromatin contact maps, and would likewise not expect a dose-dependent response to the drugs, both of which we do observe.

Dinoflagellate RNA Polymerase is indeed known to be sensitive to α-amanitin based on previous studies dating back to the 1970s (Rizzo 1979[5]), although those studies also noted some residual activity, which probably explains the dose dependence that we observe in our experiments. Nevertheless, we do agree that inhibition ought to be demonstrated directly within the context of our experiments.

---

[3] Nora EP, Goloborodko A, Valton AL, Gibcus JH, Uebersohn A, Abdennur N, Dekker J, Mirny LA, Bruneau BG. 2017. Targeted Degradation of CTCF Decouples Local Insulation of Chromosome Domains from Genomic Compartmentalization. *Cell* **169**(5):930–
[4] .e22.

[5] Rizzo PJ. 1979. RNA synthesis in isolated nuclei of the dinoflagellate *Crypthecodinium cohnii*. *J Protozool* **26**(2):290–294.

However, RNA-seq itself at the level of gene expression is likely not informative about the extent of inhibition, as it is generally thought that there is very little regulation at the level of transcription in dinoflagellates, and substantial regulation achieved through modulation of transcript stability and translation. Thus transcript levels tell us little about actual transcriptional activity.

The optimal experiment for assessing transcriptional activity is metabolic labeling using a nucleotide analog such as 4SU/4TU and then conversion of newly incorporated thiol-uraciles into cytosines following the SLAM-seq or TUC-seq protocols. Such an experiment has been carried out once in a dinoflagellate previously, in *Karenia brevis*[6]. In that case it was found that *Karenia* does not incorporate 4SU, likely due to poor intake, but does incorporate 4TU, and that was used to determine the half-lives of mRNAs for that species. The mean half life was ~40 hours and the median half life was 33 hours, and examination of that dataset showed that none of the chromatin-related genes (histones, DVNPs, RNA polymerase) exhibits half lives shorter than the median. If we are to take these results as a guidance, it is unlikely that effects at the level of transcript levels for particular genes had any significant influence over the results we observe at the level of Hi-C domain.

However, these results were in *K. brevis*, not a *Symbiodinium* species. To measure nascent transcripts, we have carried out numerous attempts to metabolically label transcripts in *B. minutum*. However, it appears that *B. minutum* cells are not permeable not just to 4SU, but also to other nucleotide/nucleoside analogs, such as 4TU, which precludes the possibility of using SLAM-seq/TUC-seq for this purpose.

In the absence of a SLAM-seq/TUC-seq experiment, the next best available way of assessing transcriptional activity is to look for nascent RNAs by measuring the fraction of unspliced intronic reads in RNA-seq datasets, although this provides more of a qualitative than quantitative measure of inhibition (as we know very little about the stability of unspliced transcripts in dinoflagellate cells). Therefore we carried out another time course of transcriptional inhibition and performed RNA-seq experiments to measure nascent RNA by calculating the fraction of unspliced intronic reads as a proxy for nascent transcription. We observe reduction of nascent RNA in both α-amanitin- and triptolide-treated cells (Supplementary Figure 30 in the revised submission).

Regarding secondary effects of inhibition in the nucleus, we do not observe large-scale change in transcript levels between treated and untreated cells (Supplementary Figure 31 in the revised submission), making it unlikely that the effects we observe are due to changes in gene expression of particular sets of genes (with all caveats already mentioned above about interpreting the significance of transcript levels in dinoflagellates). Moreover, we again cite the long half-life of dinoflagellate mRNA and that no chromatin-related genes (specifically, histones, DVNPs, or polymerases) exhibited half-lives shorter than the median in the *Karenia* study. We believe this point from the literature, combined with

[6] Morey JS, Van Dolah FM. 2013. Global analysis of mRNA half-lives and de novo transcription in a dinoflagellate, *Karenia brevis*. *PLoS One* **8**(6):e66347.

our observation of very few and modest transcript level changes after inhibition, is evidence that decompaction of dinoTADs after transcription inhibition is most likely due directly to loss of transcription, and not due to secondary effects.

6. It is unclear what results led the authors to conclude that supercoiling must be involved in the formation of contact domains in B. minitum. Although the authors name the contact domains observed in Hi-C as "TADs", there is no evidence that these domains are "topological" in any organism. The positive/negative supercoiling generated during transcription is released as Pol II advances and it does not accumulate at the 3 of genes. Domains observed in Hi-C data are composed of interactions mediated by proteins that are sufficiently closed to be crosslinked by formaldehyde, which is a short-range (2-3 A) cross-linker. Therefore, proteins mediating interactions in Hi-C are probably interacting biochemically with each other. A contact domain is formed because of changes in the directionality of interactions, which is probably determined by the nature of the proteins present in different regions of the genome rather than by some physical force caused by supercoiling. In other organisms there is no correlation between the location of supercoils determined by psoralen experiments and location of domain boundaries. In addition to inhibiting transcription, triptolide and a-amanitin result in degradation of Poll II, which may also affect the recruitment of other components of the transcription process. Performing ChIP-seq with antibodies to Pol II phosphorylated in Ser2 or ser5, if the polymerase of B. minitum has CTDs, may be a way to explore this question in more detail. In yeast cohesin accumulates at the 3 ends of genes as a consequence of transcription. If this happens in B. minitum and cohesin makes a loop between the 5 and 3 while it happens (some evidence for this is presented in Zheng et al Nature 2019 PMID 30778195), it may explain the formation of domains in dinoflagellates. This possibility is also supported by results in Drosophila where single DpnII fragment resolution (250 bp on average) Hi-C data suggest the formation of loops between the 5 and 3 of individual genes. Higher resolution Hi-C maps for B. minitum may allow the authors to map subdomains within the dinoTADs and analyze their correlation with genomic and transcription feature. A more accurate map of boundaries may allow the authors to gain insights into these different possibilities.

We would like to emphasize that transcription-induced torsional strain is our leading hypothesis for a force that underlies the formation of dinoTADs. DNA supercoiling (1) accumulates directionally in response to transcription[7,8], (2) has been reported to generate super-structures and Hi-C contact domains in the genomes of some organisms[9], and (3) remodels chromatin[10]. Our inhibition experiments

[7] Liu LF, Wang JC. 1987. Supercoiling of the DNA template during transcription. *PNAS* **84**(20):7024–7027.

[8] Ma J, Bai L, Wang MD. Transcription Under Torsion. 2013. *Science* **340**:1580–1583.

[9] Le T et al. High-Resolution Mapping of the Spatial Organization of a Bacterial Chromosome. 2013. *Science* High-Resolution Mapping of the Spatial Organization of a Bacterial Chromosome:731–734.

[10] Teves SS, Henikoff S. Transcription-generated torsional stress destabilizes nucleosomes. 2014. *Nat. Struct. Mol. Bio.* **21**(1):88–94.

demonstrate that the act of continued transcription is necessary for the maintenance of domains in dinoflagellates.

Our supercoiling hypothesis is not formulated in isolation. In other eukaryotes, psoralen binding domains are indeed observed to correspond to gene boundaries, and in mammals, topoisomerases localize to CTCF-marked loop boundaries[11].

The fact that this has not been observed in other eukaryotes likely speaks to the uniqueness of dinoflagellate nuclear organization, specifically the loss of histones, which eliminates the interactions between nucleosomes and between similarly modified histones that are observed in conventional eukaryotes, together with the length of tandem gene arrays and the uniformity of their transcription rates. These features allow transcription-induced torsional stress to manifest itself in the formation of contact domains of likely supercoiling origin.

However, it is a mistake to think that because dinoflagellates have lost nucleosomes as their main packaging component their DNA is therefore naked and there is nothing to be crosslinked – dinoflagellates have DVNPs and HLPs. Whether these proteins form specific interactions with each other is not known at present as neither they nor their post-translational modifications have been studied in detail, but strong interactions are not necessary to explain the crosslinking to each other of distal regions of the genome in a highly supercoiled state. First, such supercoiling-driven domains are well known in prokaryotes as already discussed in the manuscript, second, polymer simulations show that supercoiling can in fact bring very distal regions of DNA in very close proximity to each other (see for example Krajina & Spakowitz 2016[12]), in which context we can reasonably expect DVNPs to be crosslinked both to DNA and to each other.

To address some of these critiques, we carried out an additional Hi-C experiment in which the denaturation step was omitted (which would in theory better preserve protein-protein contacts), but while we observe accentuation of the strength of dinoTADs in that dataset, we do not find signs of specific contacts.

As noted above, the map generated by pooling contacts across multiple libraries is of sufficient depth to detect loops for the size of the *B. minutum* assembly, but we have carefully manually scanned the whole genome and observe no evidence for any such features.

It is unfortunately not possible to carry out ChIP-seq for Pol II in dinoflagellates because their CTD tails lack the phosphorylation motifs typical to conventional eukaryotes[13] and there is no antibody that reliably recognizes any of the dinoflagellate Pol II subunits.

---

[11] Uuskula-Reimand L et al. Topoisomerase IIβ interacts with cohesin and CTCF at topological domain borders. 2016. *Genome Biology* **17**:182.

[12] Krajina BA, Spakowitz AJ. 2016. *Biophys J* **111**(7):1339–1349. Large-Scale Conformational Transitions in Supercoiled DNA Revealed by Coarse-Grained Simulation.

[13] Marinov GK, Lynch M. 2015. Diversity and Divergence of Dinoflagellate Histone Proteins. *G3 (Bethesda)* **6**(2):397–422.

7. I wonder if the authors could learn something about mechanisms of 3D organization in B. minitum by comparing their data to that of S. cerevisiae, where genes are also closely packed. Do domain boundaries in cerevisiae form at each gene boundary, independent of the orientation, or only at sites of convergent genes?

As noted above and in the original submission, kinetoplastids are a much better "natural experiment" than *S. cerevisiae* because they, as a result of convergent evolution, share almost all of the highly divergent features of dinoflagellates – long gene arrays, *trans*-slicing, loss of transcriptional regulation, shift to post-transcriptional regulation as the main mechanism for regulation gene expression, etc. Expect for one – they have conventional nucleosomal chromatin while dinoflagellates have lost it.

Such a collection of characteristics represents as close to a controlled natural experiment as one can hope for, and kinetoplastids show no signs of domain structure associated with gene arrays (Supplementary Figure 16 in the original manuscript). We feel that this is further strong evidence for the model that we propose, in which the absence of histones allows for supercoiling domains to form while their presence and/or interactions with each other override supercoiling's manifestation in Hi-C maps in most eukaryotes. In short, we feel comparison to kinetoplastids is a more highly controlled and relevant consideration than a comparison to *S. cerevisiae*.

Reviewer 3:

In eukaryotes, genome folding is known to be driven by cohesin-mediated loop extrusion, together with CTCF in vertebrates, to establish topologically associating domains (TADs). The role of transcription in genome folding remains elusive. Besides, replication has also been linked to the establishment of genome structure.

In this paper, Marinov, Trevino, Xiang and colleagues carry out Hi-C analysis on a species of dinoflagellate, Breviolum minutem, generating 91 major pseudochromosomes, and reveal dinoTADs, appearing as squares on heatmaps, similar to TADs in metazoan genomes. But these dinoTADs are much larger than mammalian TADs. Each dinoTAD corresponds to a pair of divergent gene arrays with a boundary coinciding with convergence between gene blocks.

After transcription inhibition by different methods, these dinoTADs are weakened to different extents. The primary conclusion of this paper is that transcription-induced supercoiling is the primary force driving dinoTAD formation. Though it is plausible, there are a few concerns that require further clarification from the authors.

We thank the reviewer for the comments and suggestions.

Major comments:

1. The quality of Hi-C experiments may cause unexpected differences in heatmaps, and sometimes may complicate the interpretation. In Supplementary Table 1, the percentage of the Hi-C contacts over the raw read pairs differs for different experimental conditions. For control Hi-C, 27 and 34 degree cultures, the average percentage of Hi-C contact is 33% (27% to 41%). For samples treated with low concentration of alpha amanitin, the average percentage is 31% (26 to 40%), similar to control. Under this condition, the dinoTADs are not obviously weakened to my eyes (Supplementary Figures 21 to 23). However, when the alpha amanitin concentration is increased, the average Hi-C contact percentage drops to averagely 22% (17% to 27%), which, I think, is significantly lower than Hi-C of controls, heat shocked, and low alpha amanitin concentration treated conditions. For triptolide-treated samples, the average percentage of Hi-C contacts is 29% (25% to 33%), similar to Hi-C of controls, heat shocked and low alpha aminitin concentration treated conditions. And accordingly, the dinoTADs seem to be better maintained. Whether the percentage of Hi-C contacts in raw total paired reads can be used as an indicator of Hi-C quality can be debated. However, the correlation of low Hi-C contact percentage and weakened dinoTAD partition may not totally be a coincidence.

We appreciate the reviewer's concern and agree that controlling for Hi-C library metrics is important for interpreting results. We note that the fraction of reads that result in contacts is a function of a myriad factors having to do with DNA isolation, library prep, the details of how the sequencing run went, and many others. Thus is it is challenging to draw conclusions about the quality of the Hi-C itself from that metric alone. We plotted the ratio between Hi-C contacts and raw reads versus the strength of compaction of dinoTADs observed in the Hi-C maps, and found no positive relationship between the two (Response To Reviewers Figure 3). We also note that none

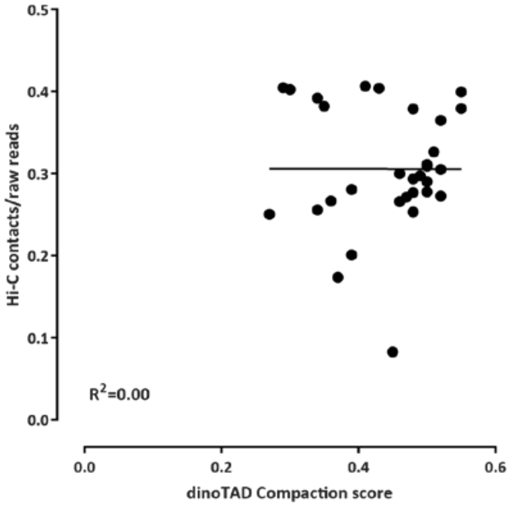

Response to Reviewers Figure 3: No relationship between the Hi-C contacts/raw reads ratio and observed dinoTAD compaction. The compaction scores were calculated as the ratio of the average contacts within a dinoTAD at a distance of 250 kbp vs. the average contacts off-the diagonal (at 50,000 kb).

of our datasets show other signs of Hi-C failure or suspect quality, such as non-uniform distribution of the four possible orientations of ligation events.

To further assuage this concern, we also reanalyzed the α-amanitin comparison by subsampling libraries down to the same number of Hi-C contacts (Response To Reviewers Figure 4); this did not affect results. We had in fact carried out this analysis before the original submission, since we too had shared these concerns.

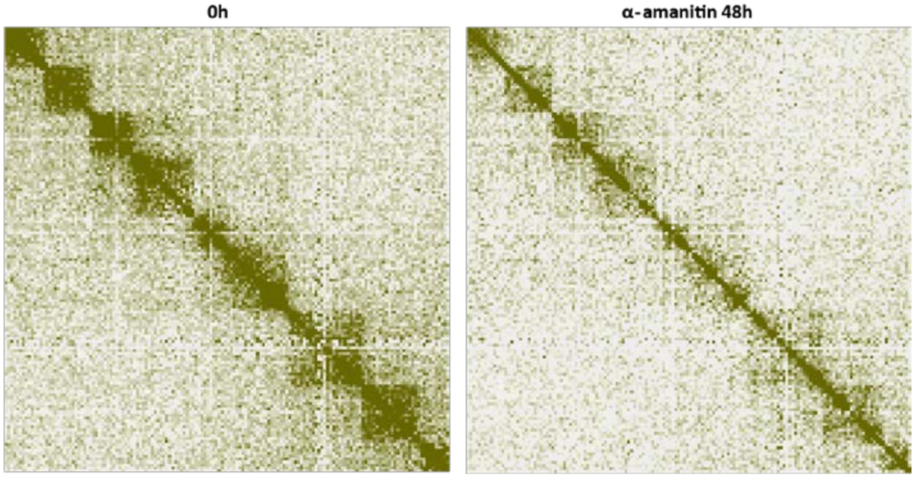

Response to Reviewers Figure 4: Subsampling Hi-C libraries to the same number of contacts (down to the number of contacts in the less deeply sequenced one) reveals the same pattern of dinoTAD compaction as observed with all reads. Shown is pseudochromosome 10 as in the main figures (the libraries shown are the “α-amanitin 0h” and “α-amanitin 48h” from the top row in Supplementary Figure 22).

2. If transcription inhibition does degrade dinoTADs (Figure 2), we may well expect that after the removal of the inhibiting drugs, transcription will resume and dinoTADs will reappear. The authors may consider to do some Hi-C on cells recovering from transcription inhibition and to show if dinoTADs will reemerge.

We have now carried out such an experiment, in which we inhibited transcription for 48 hours with α-amanitin and triptolide, then removed the drug, and carried out Hi-C after another 48 hours. We do observe partial restoration of domains at that point, but it also appears that transcription may not have fully restarted by that time (based on our estimates of nascent RNA in RNA-seq datasets). We would continue the time course, but we are concerned that at much further past that point, cells dividing and potentially restoring domains through other mechanisms could become a confounding factor.

3. In Figure 2H, topological domain formation is proposed to be established by transcription. If unidirectional genes are in a block twisted together and separated from genes of different orientation in the adjacent block, which is still in the same dinoTAD, we would expect to see two sub-TADs within a large dinoTAD. Actually, if we examine the Hi-C heatmap plotted at 5kb, indeed, sub-TADs corresponding to single unidirectional gene blocks can be observed (Supplementary Figures 3-13). The authors may reexamine the heatmaps of Hi-C at high resolution (5kb) to reveal to what extent these substructures exist in the genome.

We thank the reviewer for pointing out this trend, which we have observed ourselves too. Indeed, this trend is not only observable in the Supplementary Figures but also in Figure 1B and 1C (all of these figures are already at 5-kb resolution). However, global aggregate analyses obscure this trend because of the wide variation in local gene array structure. We have revised text to more directly point to that relationship.

4. Following point #3, florescence in situ hybridization (FISH) may be carried out to show if genes within the same block are closer than genes in adjacent blocks that are in the same dinoTAD.

We expect direct imaging approaches to resolve a lot of remaining questions about dinoflagellate chromosome organization, but these experiments are part of future efforts and are beyond the scope of the current study.

5. Another force that the authors overlooked is replication. How long is the cell cycle? The time length of transcription inhibition may just be enough to reduce gene expression to a level that arrests cells at a specific phase of cell cycle. The authors may need to consider inhibiting replication as well to see if the dinoTADs will be weakened.

We agree with the reviewer that replication could be a driving force for topological strain. However, we note that *B. minutum* cells grow slowly (with doubling times of several days), meanwhile the effects we observe with triptolide (a relatively fast acting inhibitor) are already visible at 8 hours, and those with α-amanitin (which takes at least 12 hours to begin acting, likely due to slow uptake) are very strong at 24 hours. Due to this separation of timescales, we believe replication is unlikely to be confounding the effects of decompaction following transcription inhibition. The question how dinoflagellate chromosome structure changes during cell cycle progression is an important one, but addressing these questions will require establishing protocols for synchronizing and/or isolating specific subpopulation of cells. Given

the discrepancy in timing of cell growth versus the relatively fast acting effects following transcription inhibition, we believe this type of experiment to be beyond the scope of the current study.

Minor comments:

1. Convergent gene transcription at borders was reported in Drosophila genome by Hou and colleagues in 2012 (Mol. Cell). Despite the huge differences between the genomes of insects and dinoflagellates, it would be interesting to discuss if convergent genes frequently found at boundaries could be evolutionarily conserved.

We agree that investigating the relationship between genome architecture and evolutionary conservation is a fascinating area of investigation. However, metazoans and dinoflagellates are separated by about a billion and a half years of evolution, and dinoflagellates also represent a radical departure from conventional genome organization as their genes have become organized into arrays. Essentially no conservation of synteny exists across such vast evolutionary time scales and radically divergent genomic architectures. This does not preclude a further analysis of the phenomenon of synteny at topological domain boundaries in metazoans, which we expect might yield more fruitful insights in future studies.

2. Could the authors use any metrics to quantitatively measure the differences, for example, in the border separation, or directionality index, or the dinoTADs internal strength?

We thank the reviewer for this suggestion, which sharpens our existing qualitative and quantitative descriptions of the topological phenomena observed.

We have found a metric that we believe captures the behavior of dinoTADs called a “compaction score”. This is calculated as the ratio of the average contacts within a dinoTAD at a certain distance relative to the average contacts just off-the diagonal (also used in the Response To Reviewers Figure 3). How this metric capture decompaction in an α-amanitin time course is shown in Response To Reviewers Figure 5.

As this metric differs slightly from the “insulation score” metrics that have been used previously in the HiC literature, for example, to quantify TAD strength in mammalian cells, we feel it necessary to note how the mechanisms of formation of dinoTADs differ from those of mammalian TADs. They are not quite “insulation” domains in the way that mammalian TADs are, i.e., in having a boundary that “blocks” interactions across it. The formation of these potentially supercoiled domains in fact makes very long-range contacts across dinoTAD boundaries more likely, when regions of neighboring supercoiled domains come in close contact to each other, which they would not do as often if they were not supercoiled (see the polymer simulation studies referred to above), and vice versa, the dissolution of the domains also decreases the absolute level of contacts across dinoTADs boundaries. The standard TAD insulation scores thus often do not fully capture the quite clearly observable disappearance of dinoTADs upon transcriptional inhibition.

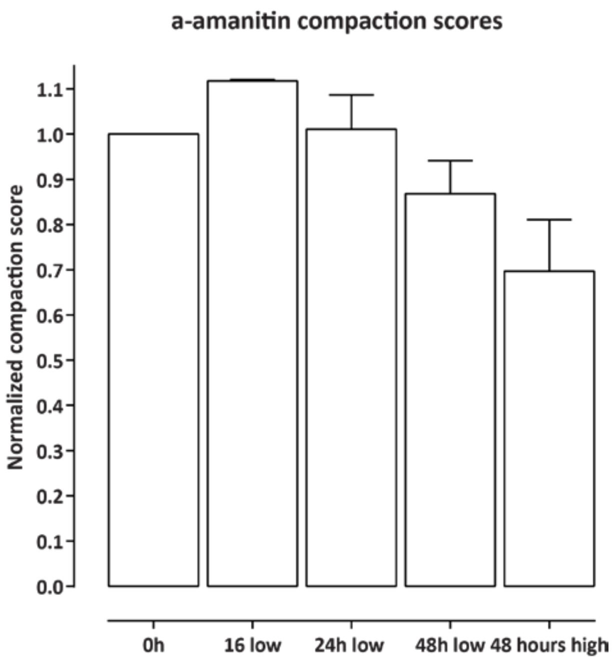

Response to Reviewers Figure 5: Quantifying dinoTAD compaction and decompaction using compaction scores. The compaction scores were calculated as the ratio of the average contacts within a dinoTAD at a distance of 250 kbp vs. the average contacts off-the diagonal (at 50,000 kb), then normalized to the matching untreated sample.

3. Color bars are missing for nearly all Hi-C heatmaps in both Figures and supplementary materials.

We have now added color bars to all metaplots; we apologize for this omission.

For context, local Hi-C maps have often not featured color bars in the Hi-C literature as the normalizations applied make the units in Hi-C matrices not directly interpretable in the same way that RPM/TPM/RPKM/FPKM units are in ChIP-seq/ATAC-seq/RNA-seq/etc. datasets.

Decision Letter, first revision:

20th Jan 2021

Dear Georgi,

Your Brief Communication, "Transcription-dependent domain-scale 3D genome organization in dinoflagellates" has now been seen by the 3 original referees. You will see from their comments below that while reviewer #1 is satisfied, some important points continue to be raised by the other two referees, especially #2.

We are interested in the possibility of publishing your study in Nature Genetics, but would like to consider your response to these serious criticisms in the form of a revised manuscript. The reviewers' concerns about the quality/analysis of the datasets need to be comprehensively and definitively addressed, if necessary by generating some new datasets.

We therefore invite you to revise your manuscript taking into account all reviewer comments. Please highlight all changes in the manuscript text file. At this stage we will need you to upload a copy of the manuscript in MS Word .docx or similar editable format.

We are committed to providing a fair and constructive peer-review process. Do not hesitate to contact me if there are specific requests from the reviewers that you believe are technically impossible or unlikely to yield a meaningful outcome. I would be happy to discuss the reviewers' points in detail.

When revising your manuscript:

*1) Include a “Response to referees” document detailing, point-by-point, how you addressed each referee comment. If no action was taken to address a point, you must provide a compelling argument. This response will be sent back to the referees along with the revised manuscript.

*2) If you have not done so already please begin to revise your manuscript so that it conforms to our Brief Communication format instructions, available
here.
Refer also to any guidelines provided in this letter.

*3) Include a revised version of any required Reporting Summary:
https://www.nature.com/documents/nr-reporting-summary.pdf
It will be available to referees (and, potentially, statisticians) to aid in their evaluation if the manuscript goes back for peer review.
A revised checklist is essential for re-review of the paper.

Please be aware of our guidelines on digital image standards.

Please use the link below to submit your revised manuscript and related files:

[REDACTED]

Note: This URL links to your confidential home page and associated information about manuscripts you may have submitted, or that you are reviewing for us. If you wish to forward this email to co-authors, please delete the link to your homepage.

Please do not hesitate to contact me if you have any questions or would like to discuss these revisions further.

Nature Genetics is committed to improving transparency in authorship. As part of our efforts in this direction, we are now requesting that all authors identified as ‘corresponding author’ on published papers create and link their Open Researcher and Contributor Identifier (ORCID) with their account on the Manuscript Tracking System (MTS), prior to acceptance. ORCID helps the scientific community achieve unambiguous attribution of all scholarly contributions. You can create and link your ORCID from the home page of the MTS by clicking on ‘Modify my Springer Nature account’. For more information please visit please visit www.springernature.com/orcid.

We look forward to seeing the revised manuscript and thank you for the opportunity to review your work.

Sincerely,

Tiago

Tiago Faial, PhD
Senior Editor
Nature Genetics
https://orcid.org/0000-0003-0864-1200

Reviewers' Comments:

Reviewer #1:
I have gone over the authors' changes and response to the reviews, and feel that they have addressed my comments adequately.

Reviewer #2:
Remarks to the Author:
The authors did not properly addressed most of my initial concerns. The fact that Reviewer 3 also had some of the same concerns suggests that the authors should not just argue their way out of the questions posed by the reviewers. After reading the manuscript again, I still think the following issues that should be addressed:

1. The view of 3D organization is not accurate. Figure 3 in the review cited in reference 1 clearly shows two types of self-associating domains, one corresponding to transcription and a second one corresponding to CTCF loops. Dixon et al Nature 2012 already showed that some TADs were flanked by CTCF sites but others were not. This was more recently clarified by Rao et al Cell 2017, who showed the existence of CTCF loops and “compartment domains”. “Compartments” are not just the plaid pattern interaction signal away from the diagonal. These interactions take place between contact

domains visible at the diagonal of Hi-C heatmaps, which Rao et al refer to as “compartment domains”. Szabo et al 2019, cited by the authors in Ref 1, show these two types of domains in their review. As I said in my previous review, by defining TADs in the introduction as equivalent to CTCF loops and then naming contact domains in B. minutum, which lacks CTCF, as dinoTADs, the authors are likely to confuse the readers.

2. Table S1. As I mentioned in my previous review, the strength of the conclusions of the manuscript depend on the quality of the Hi-C data. Most analyses in the manuscript use intra-chromosomal interactions over long genomic distances. Therefore, the fraction of interchromosomal reads and reads <20 kb in the column “Number of Hi-C contacts” of Table S1 do not contribute to these calculations. It would be informative and help the reader judge the validity of the conclusions if the authors include additional columns with this information in Table S1. The refusal of the authors to provide this information is perplexing. Furthermore, although the Hi-C libraries from the normal condition may have sufficient reads, the rest of the libraries do not, which will affect comparisons among conditions.

3. Page 3. “DinoTAD number correlates with chromosome size (Fig. 1D), and they are considerably larger than mammalian TADs (Fig. 1E).” Please note that these conclusions are irrelevant, since the number of TADs and their size depends on the resolution of the data. One can take the 4.5 million contacts from a specific cell type from Rao et al Cell 2014, downsample the reads to various numbers, call TADs, and find that the TAD size increases and TAD number decreases as the number of contacts used for the analysis decreases.

4. Page 3 and Figures 1G-H. Please explain this in more detail so that the reader can evaluate the significance of this conclusion. Did authors use kb resolution i.e. 5 kb bins to map the location of the boundaries? If so, does this mean that the boundaries are within +/- 5 kb of the site were genes converge? What is the distance between convergent genes? This information is important to understand possible mechanisms by which the dinoDomains form.

5. Figure 2D. Since the Hi-C libraries in the a-amanitin and triptolide samples have fewer reads, were the treatment libraries downsampled for this analysis? Please describe the resolution at which these comparisons were performed.

Minor comments

1. Bottom of page 1. How is “applied chromosome conformation capture” different from standard chromosome conformation capture. Please explain.

Reviewer #3:
Remarks to the Author:
The authors have performed additional experiments addressing my remarks. Most of my questions have been satisfactorily addressed. However, I am still confused by some replies from the authors.

The authors carried out additional experiments (inhibiting transcription for 48 hours with alpha-amanitin and triptolide, then removed the drug, and carried out Hi-C after another 48 hours). Surprisingly, the authors observed only partial restoration of domains. They did not carry out addition Hi-C after 48 hours with drugs removed, because the authors were concerned that “at much further

past that point, cells dividing and potentially restoring domains through other mechanisms could become a confounding factor".

One of the major points of the paper made by the authors is that the mechanism behind the formation of dinoTAD structure could be the transcription-driven supercoiling of the chromatin. I am quite surprised that the authors here are suggesting "other mechanisms" may restore the domain structure by saying "cells dividing and potentially restoring domains through other mechanisms".
I guess we can exclude replication as "other mechanism" because the cells were not synchronized at any point, basically the cells for Hi-C could be at any stage through the cell cycle in their experiment. So I am wondering exactly what "other mechanisms" the authors are considering if it is not "transcription" or not "replication".

If the authors could conduct Hi-C after 48 hours of the removal of drug, why could they carry out Hi-C at 72 or 96 hours? The authors' concern that "other mechanisms could become a confounding factor" does not seem to me compellingly convincing, especially when they did not name any other potential mechanism that "could become a confounding factor".

Author Rebuttal, first revision:

Transcription-dependent domain-scale 3D genome organization in dinoflagellates. Response to reviewer comments #2

Reviewer 2:

The authors did not properly addressed most of my initial concerns. The fact that Reviewer 3 also had some of the same concerns suggests that the authors should not just argue their way out of the questions posed by the reviewers. After reading the manuscript again, I still think the following issues that should be addressed:

1. The view of 3D organization is not accurate. Figure 3 in the review cited in reference 1 clearly shows two types of self-associating domains, one corresponding to transcription and a second one corresponding to CTCF loops. Dixon et al Nature 2012 already showed that some TADs were flanked by CTCF sites but others were not. This was more recently clarified by Rao et al Cell 2017, who showed the existence of CTCF loops and "compartment domains". "Compartments" are not just the plaid pattern interaction signal away from the diagonal. These interactions take place between contact domains visible at the diagonal of Hi-C heatmaps, which Rao et al refer to as "compartment domains". Szabo et al 2019, cited by the authors in Ref 1, show these two types of domains in their review. As I said in my previous review, by defining TADs in the introduction as equivalent to CTCF loops and then naming contact domains

in B. minutum, which lacks CTCF, as dinoTADs, the authors are likely to confuse the readers. In the last version of our manuscript introduction, we stated:

Mechanisms known to drive genome folding in eukaryotes include constraints on cohesin-mediated loop extrusion – imposed by CTCF in vertebrates – and self-associations between similar chromatin states. These generate topologically associating domains (TADs) and compartments.

In our view, this is an accurate, if brief summary. The language, in particular, the word "include," is not intended to suggest equivalence between TADs and CTCF loops, but rather that these are two of the forces that give rise, in potentially complex ways, to the observed architectures. This is consistent with Rao et al, 2017, who suggest that two mechanisms drive genome architecture in mammals:

> "The first is the cohesin and insulator protein-dependent formation of loop domains... The second mechanism is cohesin-independent compartmentalization of chromatin intervals with similar histone marks."

We agree that CTCF loops are not equivalent to TADs, and we agree that compartment-like self-association leads to both contact domains along the diagonal and the broad "plaid" contact patterns observed at larger length scales. We also acknowledge that transcribed genes have been reported to insulate domains in some cases. However, CTCF bounded domains are an archetype in mammals, CTCF sites are highly enriched at mammalian TAD boundaries, and compartments are largely not dependent on transcription in mammals. The goal of introducing a separate name in the current work, "dinoTADs", was to simultaneously evoke similarly sized contact-enriched domains and distance them mechanistically from those mammalian archetypes (more on this mechanistic distinction below, and more discussion of the domain sizes in the next points).

It was an instructive exercise to actually revisit the definition of topologically associating domain (TAD) in Szabo et al. 2019, which is quite general: "At a scale of tens to hundreds of kilobases, chromosomes fold into domains with preferential intradomain interactions compared to interdomain interactions with the neighboring cis chromatin domains, i.e., spanning domain borders." The authors go on to write, "the molecular mechanisms underlying their formation can be diverse, consistent with the existence of different types of TADs." Perhaps the most accurate response to the reviewer's semantic critique is to call the domains we have observed simply, "TADs." However, this would most likely cause more confusion than clarity as the current usage in the field, due to its focus on mammals, often conflates "TADs" with mammalian TADs. We feel strongly that the use of "dinoTADs" makes it clear these are not mammalian TADs, yet fall under the general rubric of the TAD definition as described above in Szabo et al 2019.

We are happy to take editorial guidance on clarifications that would be helpful to the reader. We have also updated the introductory text to avoid a direct conflation of CTCF loops and TADs:

Topologically associating domains (TADs) and compartments on the sub-megabase scale are conserved architectural features of eukaryote genomes, defined by increased intradomain contact frequencies and interdomain contact insulation. Mechanisms known to drive the folding of such domains include constraints on cohesin-mediated loop extrusion – imposed in part by CTCF in vertebrates – and self-associations between similar chromatin states. Other mechanisms, including insulation of domains by polymerases or specific boundary proteins, have also been proposed to play roles in genome architecture.

This leads us to the question of whether dinoTADs are produced primarily by mechanisms that are distinct from the two proposed by Rao in 2017. In subsequent points, we will discuss our data integrity, but here we focus specifically on our logic.

We see no signs of loop extrusion features despite our data sets being more than sufficiently powered to detect them, so it is quite likely that these domains are not formed as a result of constrains on loop extrusion analogous to CTCF-mediated loop domains in mammals.

This leaves the question of whether they are formed by compartment-like interactions between proteins. Dinoflagellates mostly do not have nucleosomes to mediate the same kind of interactions, but it is in principle possible that DVNPs and HLPs are a substrate for analogous epigenetic states that mediate such interactions. However, these states cannot easily explain the topological features observed in Symbiodinium Hi-C data. First, such interactions between protein states do not explain the correspondence between pairs of divergent gene arrays and dinoTADs. Second, if compartment-like interactions were forming the domains, then some form of plaid-like pattern may have been present, but it is not. Third, specific inhibition of transcription may not have decompacted the dinoTADs if they were formed by such a mechanism. This is precisely the case in mammals: In Rao 2017, after degrading cohesin, loops disappear, but compartments remain, representing the association of like histone states in the nucleus. Then, Vian et al. 2018 (which shares many of the same authors) further show that transcription inhibition has only a modest effect on both loop maintenance and intra-domain contact integrity in mammals (Figure 2B). Finally, based on a pair of studies in mouse embryogenesis, blocking transcription with alpha-amanitin does not affect the formation of TADs (De et al. 2017, Ke et al. 2017). Thus, active transcription is not required to maintain “compartment domains” in mammals.

Separately, one might also imagine that specific transcription inhibition in dinoflagellates could drastically reduce the amount of proteins mediating decompaction – for instance, if the kinetics of transcribing and degrading DVNP mRNAs were much faster than the timescales of our experiments. If such a secondary effect were in fact mediating decompaction, then we would have expected to observe that reduction in DVNP mRNA by RNA-seq, but we do not.

In the end, it is difficult for us to imagine how epigenetic state interactions could achieve a directionality which matches the directionality of gene transcription, and be dependent on active transcription at the hours-days timescale, without somehow being related to transcription.

Let us also clarify that we believe an “accurate” view on genome topology does not exist in a final sense. In addition to the ability to insulate some contact domains (referenced, as the reviewer notes, in Figure 3 of Szabo et al. 2019), transcription may indeed impact genome architecture profoundly in mammals, at sub-TAD length scales (Hsieh et al. 2020). Likewise, by stating our observations – transcription inhibition decompacts TADs of a certain size in dinoflagellates – and our primary hypothesis resulting from this observation - physical torque caused by RNA polymerases alters the genome structure - we are not precluding the involvement of other forces, including protein-protein interactions. We think our supercoiling hypothesis – for which there is precedent in prokaryotes – is the best explanation available based on our results.

> 2. Table S1. As I mentioned in my previous review, the strength of the conclusions of the manuscript depend on the quality of the Hi-C data. Most analyses in the manuscript use intra-chromosomal interactions over long genomic distances. Therefore, the fraction of interchromosomal reads and reads <20 kb in the column “Number of Hi-C contacts” of Table S1 do not contribute to these calculations. It would be informative and help the reader judge the validity of the conclusions if the authors include additional columns with this information in Table S1. The refusal of the authors to provide this information is perplexing. Furthermore, although the Hi-C libraries from the normal condition may have sufficient reads, the rest of the libraries do not, which will affect comparisons among conditions.

We apologize for misunderstanding the reviewer’s previous requests. We would like to assure the reviewer our intention was not to prevent the release of these data. A particular subset of columns of the Juicer output was originally provided for no other reason than to avoid cluttering the table with too many columns, as we felt the previously displayed statistics were the most important to clearly display at the time. We have now added the additional columns to the table (intra/interchromosomal reads and ≤/≥ 20 kbp contacts).

We would note that some of these QC outputs from non-model species often cannot be compared directly to those from mammals, e.g. Rao 2014. For example, while Hi-C scaffolding is very powerful in terms of creating pseudochromosomes, it is not omnipotent, and it leaves some small contigs outside the main pseudochromosomes. If they are filtered from the reference genome, interactions in those regions will be unmapped; if they are kept, the interactions will count as “interchromosomal” computationally – but their actual physical origin is not interpretable as such. Similarly, it is unproductive to use the ≤/≥ 20 kbp contacts ratio on its own as a QC metric for α-amanitin libraries, since the disappearance of domain compaction is what the effect of those treatments is.

In terms of the comparisons of the different data sets across conditions, we are unsure how our conclusions would be affected by the differences in read depth to which the reviewer is referring, as there are no such major differences between the conditions. Our major conclusions, i.e. that the strength of the contact domains change across the various perturbations we measure, is not affected by the differences in sequencing depth between those datasets. A larger resolution (50-kb) is used for

those comparisons, and, most importantly, there is no major difference in sequencing depth between them. We also specifically examined the effect of downsampling the matrices across conditions to the same number of reads, reaching identical conclusions, as discussed in the previous round of review (see p. 9 and p.10 and Response to Reviewers Figure 4 in that document).

We would also like to point out that we carried out multiple time courses and generated both biological and technical replicates for all conditions, with the results reproducing throughout all of them. This means that none of our results hinges on a particular individual library that might have been of lower quality (and we have not seen signs of such issues for any of the libraries included in the manuscript) and it is extremely unlikely that when a result is replicated by 3 to 4 different sets of libraries, it is an artifact of data quality that only affects a subset of samples. Moreover, Hi-C libraries for all time courses were processed at the same time to minimize batch effects.

Still, to show beyond any doubt that conclusions are not affected by such factors, we have now resequenced key libraries to a much greater depth and shown that the disappearance of dinoTADs upon α-amanitin treatment – and thus our conclusions - hold (Response To Reviewers Figure 1).

> 3. Page 3. "DinoTAD number correlates with chromosome size (Fig. 1D), and they are considerably larger than mammalian TADs (Fig. 1E)." Please note that these conclusions are irrelevant, since the number of TADs and their size depends on the resolution of the data. One can take the 4.5 million contacts from a specific cell type from Rao et al Cell 2014, downsample the reads to various numbers, call TADs, and find that the TAD size increases and TAD number decreases as the number of contacts used for the analysis decreases.

Before we discuss this critique, we would like to reiterate the main results of our study, and how they were derived.

1. Dinoflagellate genomes exhibit strong interaction domains
2. These domains closely coincide with pairs of divergent gene arrays
3. They do not exhibit any signs of being formed through imposing constraints on loop extrusion
4. They are transcription-dependent, as transcription inhibition leads to their degradation.
5. These observations are currently best explained by a model in which domains are formed by transcription induced supercoiling.

These conclusions are reached primarily by examining maps over the whole genome, which does not in any way depend on calling TADs. After a careful manual examination of the whole genome (snapshots for ~15% of it are

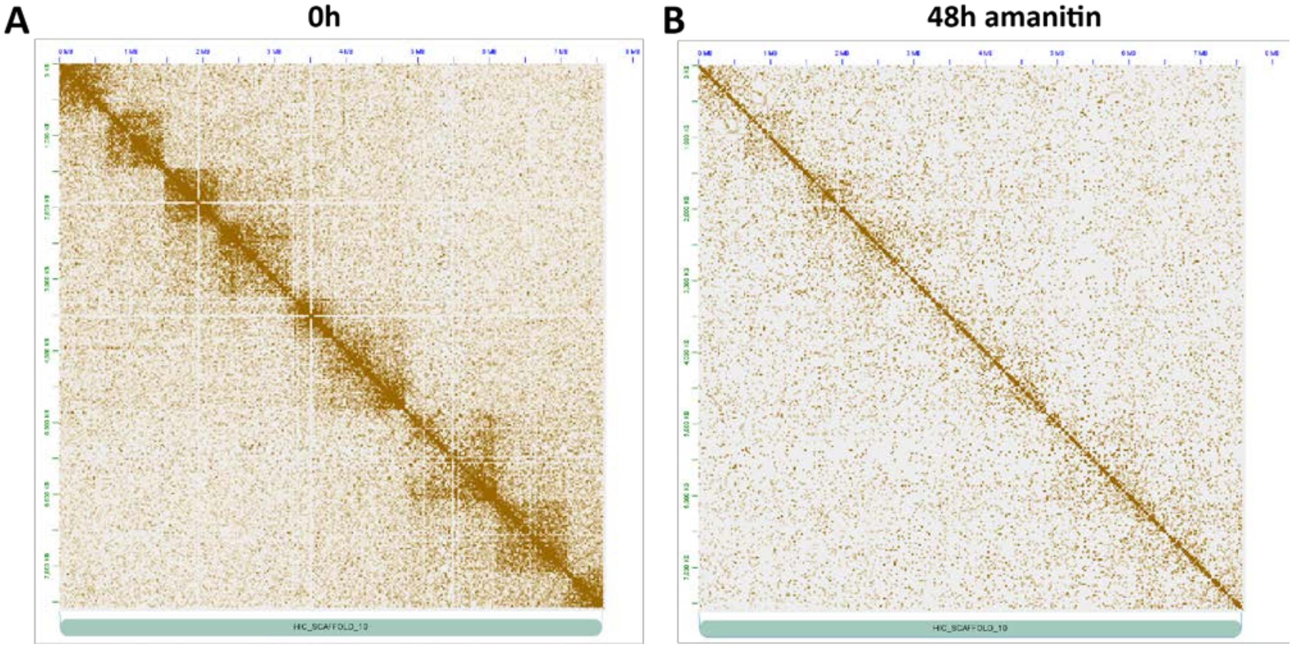

Response to Reviewers Figure 1: Deeper sequencing of α-amanitin treated and control samples reveals the same patterns of erosion of dinoTADs as observed with shallower libraries. The two libraries were sequenced to a depth of ~117 million and ~121 million contacts for the 0-hour and the 48-hour α-amanitin, respectively

shown in the Supplementary Materials), the conclusions were confirmed and generalized by examining aggregate metaplots based on automated TAD calling.

As we discussed on p.4 and p.5 in the previous Response To Reviewers, conclusions regarding overall topological features were carried out on a map that, taking into account the quadratic relationship between the size of the Hi-C matrix and genome size, and the size of the *B. minutum* assembly we are working with relative to the human genome, is at least as deep as the 4.5-billion contact map from Rao et al. 2014. This means that loop extrusion features should have been abundantly evident in our data (as demonstrated in our previous response, where we showed that these features are readily detectable with <100M contacts for a 1.3-Gbp vertebrate genome that we had assayed for an unrelated project), but they are not.

Do separate contact domains exist in this organism, independent of gene array boundaries? It is true that ultrahigh-resolution maps generated using Micro-C have recently uncovered previously unappreciated domain structure in mammals at a sub-TAD scale. However, that has not changed the definition of the TADs at larger scales. Similarly, the main result regarding dinoflagellate TADs is that

they closely coincide with gene arrays, and this is a very clear based on visual inspection of the genome at high resolution, and it is immediately obvious by examining maps at any resolution finer than the length of gene arrays. If finer scale structures are eventually discovered with, for example, 50-billion contact maps on these ≤1-Gbp genomes, this finding would not invalidate the existence of the larger dinoTADs as presented in this work.

The features we describe are two orders of magnitude larger than our effective interaction resolution. We are interested primarily in their characteristics at those length scales and discuss them in this context. We needed to set a threshold and define our working unit, so to speak, of analysis, and to do so in an automated way. The metaplots shown in Figure 1, which are aggregated around automatically called TAD boundaries, show that these units match the features observed by visual examination of maps quite well.

However, in order to do our utmost to address the reviewer's concerns as we have interpreted them, we have nearly doubled the depth of our map under untreated conditions, by carrying out additional sequencing and combining libraries from the untreated conditions from the time courses, to ~1.05B contacts. This is, on a per-basepair basis, even given the relative linear sizes of the genomes, above the depth of Rao et. al. 2014. There were no changes to our conclusions from our deep map and our original map (Response To Reviewers Figure 2).

In addition, we have explored the parameter space of our TAD caller, including bin size, to explicitly show how binning and parameters affect reported TAD calls. Based on this analysis, we conclude broadly that a higher bin resolution makes the calls more sensitive to the parameters. It is possible to call larger or smaller TADs on our high-resolution data, but this is arguably primarily a function of the parameters. Calls at larger resolutions were more stable around the 600-900 kb size range and produce between 500 and 800 domains, consistent with the calls we made in our maps (Response to Reviewers Figure 3)

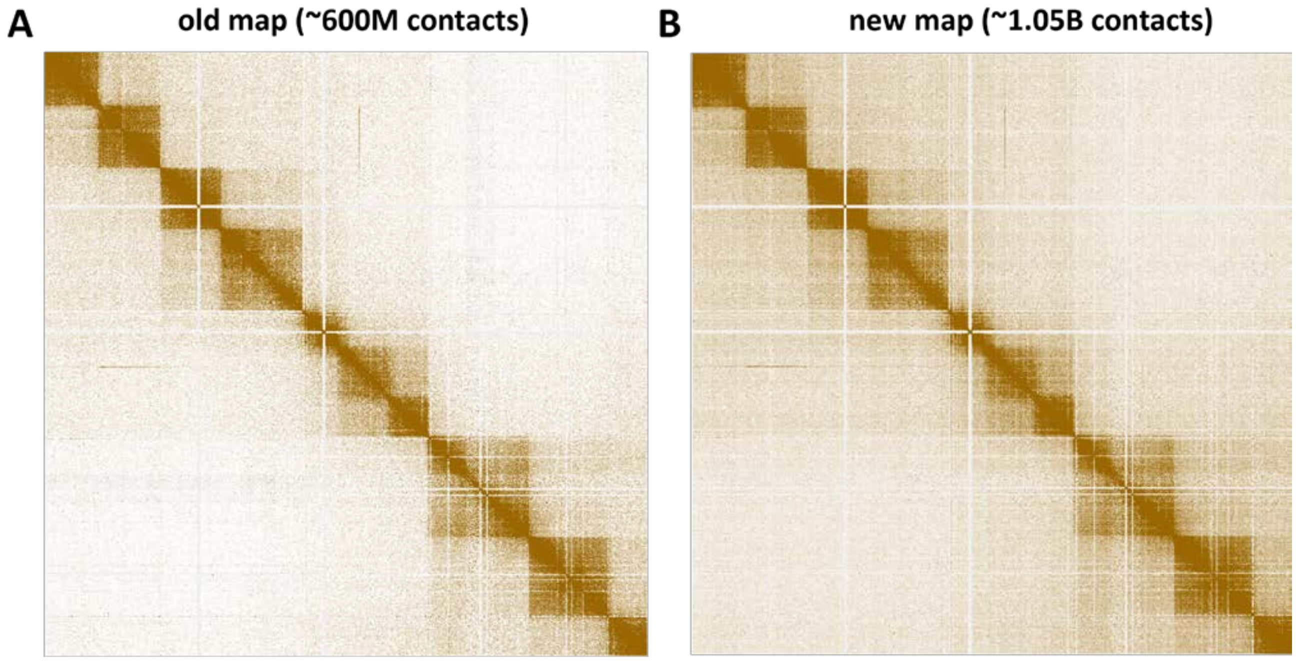

Response to Reviewers Figure 2: Deeper sequencing map across untreated conditions reveals an identical picture to the map originally used in the manuscript. Libraries from all untreated conditions together with additional sequencing were combined, with identical results to what was originally presented.

To further attempt to allay the reviewer's concerns, we have edited this sentence to state: "Detected DinoTAD number correlates with chromosome size (Fig. 1D), and observed DinoTADs are considerably larger than mammalian TADs (Fig. 1E)."

> 4. Page 3 and Figures 1G-H. Please explain this in more detail so that the reader can evaluate the significance of this conclusion. Did authors use kb resolution i.e. 5 kb bins to map the location of the boundaries? If so, does this mean that the boundaries are within +/- 5 kb of the site were genes converge? What is the distance between convergent genes? This information is important to understand possible mechanisms by which the dinoDomains form.

We apologize for the confusion here. The results presented use domains defined with 50 kb bins, which was stated in the Methods and we have now added an additional explicit reference to that in the figure legend. We used the HiCExplorer tool "hicFindTADs" on matrices binned at 10 kb, 25 kb, and 50 kb, using default settings and FDR correction. These outputs produced similar, but not identical results. Visual inspection suggested that the boundaries of the 50 kbp maps matched visually defined domains and

gene arrays best, and were thus a better anchoring point for our downstream comparisons of dinoTAD strength.

We would also be happy to provide more details about this analysis and the interpretation.

Regarding gene arrays, which, as mentioned above, are defining the dinoTADs, the average intergenic length in this species is quite short – roughly 2 kb – which has been known for some time (Shoguchi et al 2013). The length of gene arrays in units of gene number is, even if based on the previous, fragmented assembly (but only including complete such arrays, i.e. this is probably a mostly accurate count) 20 to 50 genes. The average gene length is ~11

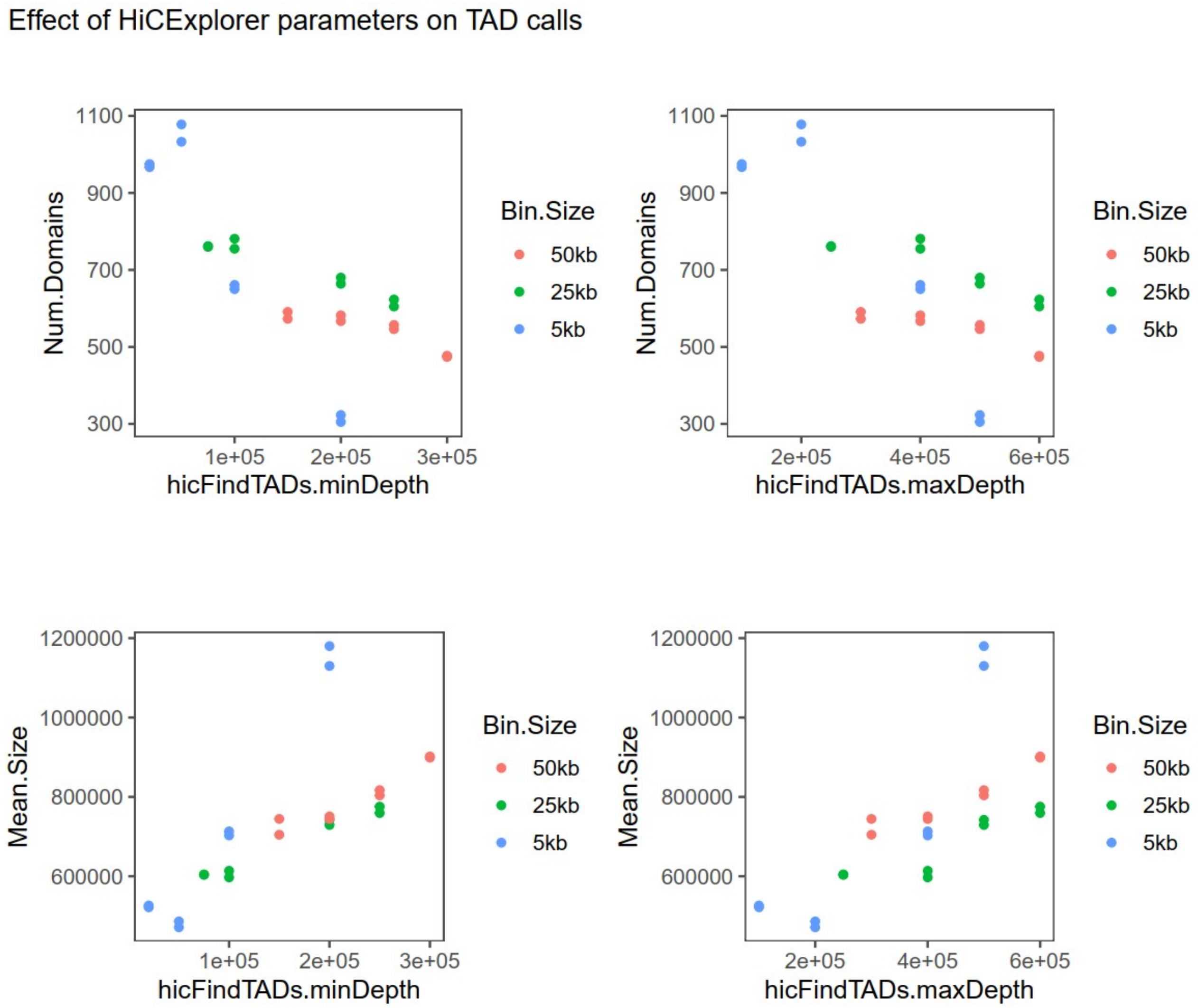

Response to Reviewers Figure 3: Comparison of domain size and number as a function of TAD calling parameters and input matrix bin size. High-resolution maps are sensitive to calling parameters, while 25kb and 50kb resolution maps produce fairly stable results.

kbp, and each dinoTAD is formed by a pair of divergently-oriented arrays. Thus, even before ever considering our evidence, gene arrays could therefore be safely assumed to be several hundred kilobases in size, with some pairs of gene arrays approaching a megabase and exceeding that size on occasions.

Several points are important to note here: First, these estimates are similar to our called domain sizes (as they should be, given the correspondence between these features). Second, the sizes are an order of magnitude larger than the boundary size we specified in our automatic TAD caller. Third, these lengths are two orders of magnitude larger than the effective resolution of our primary contact map.

As pointed out above, the primary purpose of our use of a computational domain caller was explicitly to generate an automated, systematically derived set of domain boundary calls to support our visual inspection approach with generalized results and allow a global quantification of the strength of these domains.

In their documentation, the authors of the HicExplorer package explain: “It is recommended to test multiple parameters of TAD calling with hicFindTADs before making conclusions about the number of TADs in a given sample or before comparing TAD calling between multiple conditions.” Because the features we aimed to quantify were clearly relatively large scale, we wanted to make sure the TAD calling algorithm was identifying the features that were visually obvious in the data sets. Given that consideration, and the advice of the HiCExplorer authors, we explored different resolutions in the 10–50 kb range, trying 10 kb, 25 kb, and 50 kb boundary resolutions and comparing them with each other, as well as with the manually defined domains. In the end, we felt that the 50-kb domains identified the boundaries of dinoTADs more accurately (Response To Reviewers Figure 4) These domains captured the overall patterns observed in the data, as is evident from the aggregate plots in Figure 1G and H, produced using these computational calls. This suggested to us that the broad conclusion were intact, namely, that domain boundaries coincide with convergent transcription.

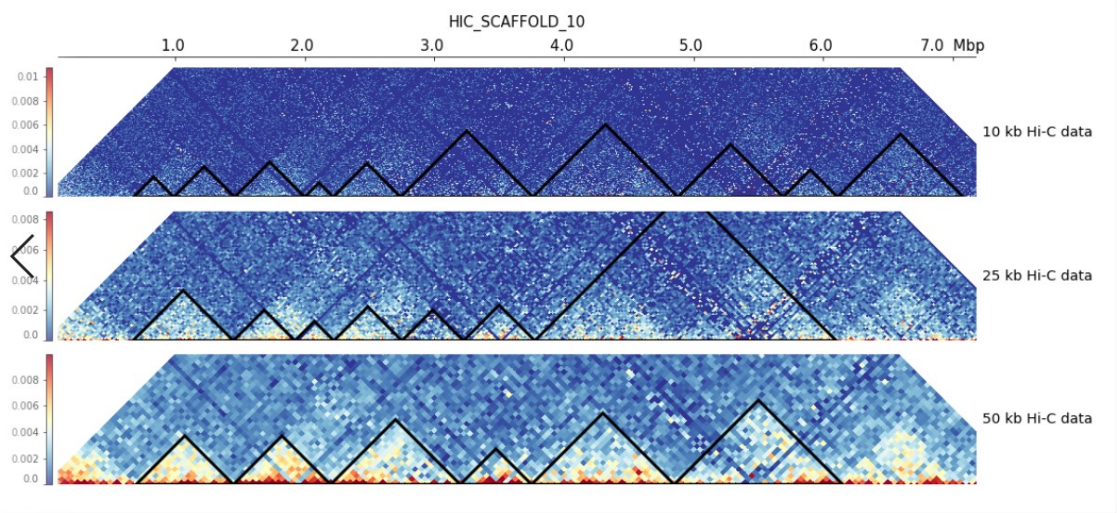

Response to Reviewers Figure 4: Optimization of TAD calling parameters. Domains were called at 10-kbp, 25-kbp and 50-kbp and compared to manually defined/visually obvious domains. The 50-kb threshold captured dinoTAD boundaries both sufficiently well on its own and better than the 10-kbp and 25-kbp domains.

To reiterate: The computational calls were performed using the deeply sequenced high-resolution contact matrices. To capture large scale features, boundaries were called at a resolution of 50 kb and validated against manual calls. Their purpose was to first, validate the visual evidence of domain insulation from 5 kb resolution contact maps; second, provide a framework to visually summarize genome-scale results; and third, establish reproducible domain calls for future re-analysis.

Regarding the comparisons between conditions, we would again like to point that results were evaluated in two independent ways. First, maps were directly visually examined and compared against each other, and the erosion of dinoTADs was observed. This does not depend in any way on TAD calls. Second, we generated metaplots based on the automated domain calling. We reached identical conclusions in both analyses.

> 5. Figure 2D. Since the Hi-C libraries in the a-amanitin and triptolide samples have fewer reads, were the treatment libraries downsampled for this analysis? Please describe the resolution at which these comparisons were performed.

As discussed and shown in the previous round of revisions (Response to Reviewers #1 p. 9 and p. 10; Response to Reviewers #1 Figure 4), the results shown in the figures are not based on downsampled matrices, but we did explore the effects of downsampling before the original submission, and we found

that results are identical with or without downsampling. The resolution of the matrices used for that analysis (50-kb) is also clearly stated in the figure legend.

As shown by the additional sequencing presented above (Response To Reviewers Figures 1 and 2), after sequencing libraries to a much greater depth our conclusions hold, as expected.

1. Bottom of page 1. How is "applied chromosome conformation capture" different from standard chromosome conformation capture. Please explain.

The sentence should have read "we applied chromosome conformation capture". We have made an additional correction to the text and thank the reviewer for pointing out this error.

Reviewer 3:

The authors have performed additional experiments addressing my remarks. Most of my questions have been satisfactorily addressed. However, I am still confused by some replies from the authors.

The authors carried out additional experiments (inhibiting transcription for 48 hours with alpha amanitin and triptolide, then removed the drug, and carried out Hi-C after another 48 hours). Surprisingly, the authors observed only partial restoration of domains. They did not carry out addition Hi-C after 48 hours with drugs removed, because the authors were concerned that "at much further past that point, cells dividing and potentially restoring domains through other mechanisms could become a confounding factor".

One of the major points of the paper made by the authors is that the mechanism behind the formation of dinoTAD structure could be the transcription-driven supercoiling of the chromatin. I am quite surprised that the authors here are suggesting "other mechanisms" may restore the domain structure by saying "cells dividing and potentially restoring domains through other mechanisms". I guess we can exclude replication as "other mechanism" because the cells were not synchronized at any point, basically the cells for Hi-C could be at any stage through the cell cycle in their experiment. So I am wondering exactly what "other mechanisms" the authors are considering if it is not "transcription" or not "replication".

If the authors could conduct Hi-C after 48 hours of the removal of drug, why could they carry out Hi-C at 72 or 96 hours? The authors' concern that other mechanisms could become a confounding factor does not seem to me compellingly convincing, especially when they did not name any other potential mechanism that "could become a confounding factor".

We apologize for the confusion here, and we appreciate the opportunity to make this clearer. We did not intend to imply that mysterious “other mechanisms would restore domains beyond 48 hours. Instead, our point was only that when the timescales of these experiments begin to substantially exceed the cell division timescale, we might worry that other processes other than steady-state gene expression to come to bear in unknown ways. For example cells that have had a chance to divide after relaxation of inhibition might regenerate their chromosomal organization as part of the process of forming a new nucleus, thereby confounding our interpretations. Clearly, if one were to wait for, for example, 3 weeks, cells would revert back to their usual state after transcriptional perturbation, but such observations would not be especially informative for our hypothesis. When designing the time course, we felt that the 48-hour time point was the “cleanest” test for our hypothesis, as data collected much later after the release of inhibition were likely to show stronger structuring, but would potentially be confounded by these other factors (chief of which is the impact of cell division)

Decision Letter, second revision:

Our ref: NG-LE55211R1

11th Feb 2021

Dear Will and Georgi,

Thank you for submitting your revised manuscript "Transcription-dependent domain-scale 3D genome organization in dinoflagellates" (NG-LE55211R1). It has now been seen by reviewer #2 and their comments are below. I have discussed the reviewer's feedback with my colleagues and we'll be happy in principle to publish your manuscript in Nature Genetics, pending minor revisions to satisfy the referees' final requests and to comply with our editorial and formatting guidelines.

We are now performing detailed checks on your paper and will send you a checklist detailing our editorial and formatting requirements in about a week. Please do not upload the final materials and make any revisions until you receive this additional information from us.

Thank you again for your interest in Nature Genetics. Please do not hesitate to contact me if you have any questions.

Congratulations!

Best wishes,

Tiago

Tiago Faial, PhD

Senior Editor
Nature Genetics
https://orcid.org/0000-0003-0864-1200

Reviewer #2 (Remarks to the Author):

I have spent a lot of my time reviewing this manuscript. I have tried to point out issues that, in my humble opinion, could be easily addressed to improve the manuscript. Obviously, these are my own views and it is possible that I'm wrong, so I will not insist that the authors perform the very minor changes I suggested in my previous review. However, I would still like to point out the following. In my previous comment #3 I raised the possibility that the number of domains will depend on the resolution of the data i.e. the size of the bins used to analyze the information. The authors argue that this is not the case and show Response to Reviewers Figure 2 to support their claim. However, Response to Reviewers Figure 4 shows exactly the issue I raised. The domains are completely different, and the boundaries are different, depending on the resolution chosen to perform the analysis. I still think that if they chose to use 50 kb bins, and the domain boundaries are therefore located at +/- 50 kb with respect to sites of convergent transcription, that the authors should make the reader aware of this point.

Author Rebuttal, final revision

Transcription-dependent domain-scale 3D genome organization in dinoflagellates. Response to reviewer comments #3

Reviewer 2:

> I have spent a lot of my time reviewing this manuscript. I have tried to point out issues that, in my humble opinion, could be easily addressed to improve the manuscript. Obviously, these are my own views and it is possible that I'm wrong, so I will not insist that the authors perform the very minor changes I suggested in my previous review. However, I would still like to point out the following. In my previous comment #3 I raised the possibility that the number of domains will depend on the resolution of the data i.e. the size of the bins used to analyze the information. The authors argue that this is not the case and show Response to Reviewers Figure 2 to support their claim. However, Response to Reviewers Figure 4 shows exactly the issue I raised. The domains are completely different, and the boundaries are different, depending on the resolution chosen to perform the analysis. I still think that if they chose to use 50 kb bins, and the domain boundaries are therefore located at ±50 kb with respect to sites of convergent transcription, that the authors should make the reader aware of this point.

We thank the reviewer for the effort invested in improving the manuscript.

As we discussed in our previous response, the goal of calling domains was not to call domains in the abstract, but to have a systematic way to define boundaries that is not dependent on manual curation but still preserves the visually obvious data structure (i.e. that dinoTAD domains correspond to gene array pairs). That domain calls are generally parameter-dependent is not an issue we have any intention disputing, the point is that the parameters that we chose to use capture the biologically relevant domain boundaries best, and this is why we use those parameters. We have added a clarification noting this in the Methods section.

Final Decision Letter:

In reply please quote: NG-LE55211R2 Marinov

17th Mar 2021

Dear Georgi,

I am delighted to say that your manuscript "Transcription-dependent domain-scale three-dimensional genome organization in the dinoflagellate Breviolum minutum" has been accepted for publication in an upcoming issue of Nature Genetics.

Prior to setting your manuscript, we may make minor changes to enhance the lucidity of the text and with reference to our house style. We therefore ask that you examine the proofs most carefully to ensure that we have not inadvertently altered the sense of your text in any way.

Once your manuscript is typeset and you have completed the appropriate grant of rights, you will receive a link to your electronic proof via email with a request to make any corrections within 48 hours. If, when you receive your proof, you cannot meet this deadline, please inform us at rjsproduction@springernature.com immediately.

Your paper will be published online after we receive your corrections and will appear in print in the next available issue. You can find out your date of online publication by contacting the Nature Press Office (press@nature.com) after sending your e-proof corrections. Now is the time to inform your Public Relations or Press Office about your paper, as they might be interested in promoting its publication. This will allow them time to prepare an accurate and satisfactory press release. Include your manuscript tracking number (NG-LE55211R2) and the name of the journal, which they will need when they contact our Press Office.

Before your paper is published online, we shall be distributing a press release to news organizations worldwide, which may very well include details of your work. We are happy for your institution or funding agency to prepare its own press release, but it must mention the embargo date and Nature Genetics. Our Press Office may contact you closer to the time of publication, but if you or your Press Office have any enquiries in the meantime, please contact press@nature.com.

Acceptance is conditional on the data in the manuscript not being published elsewhere, or announced in the print or electronic media, until the embargo/publication date. These restrictions are not intended to deter you from presenting your data at academic meetings and conferences, but any enquiries from the media about papers not yet scheduled for publication should be referred to us.

Nature Genetics is a Transformative open access journal. In approximately 10 business days you will

receive an email with a link to choose the appropriate publishing options for your paper and our Author Services team will be in touch regarding any additional information that may be required.

Please note that Nature Research offers an immediate open access option only for papers that were first submitted after 1 January, 2021.

You will not receive your proofs until the publishing agreement has been received through our system.

If you have any questions about our publishing options, costs, Open Access requirements, or our legal forms, please contact ASJournals@springernature.com

In the event that you choose to publish under the subscription model, Nature Research allows authors to self-archive the accepted manuscript (the version post-peer review, but prior to copy-editing and typesetting) on their own personal website and/or in an institutional or funder repository where it can be made publicly accessible 6 months after first publication, in accordance with our self-archiving policy. Please review our self-archiving policy for more information.

Several funders require deposition the accepted manuscript (AM) to PubMed Central or Europe PubMed Central. To enable compliance with these requirements, Nature Research therefore offers a free manuscript deposition service for original research papers supported by a number of PMC/EPMC participating funders. If you do not choose to publish immediate open access, we can deposit the accepted manuscript in PMC/Europe PMC on your behalf, if you authorise us to do so.

If you have posted a preprint on any preprint server, please ensure that the preprint details are updated with a publication reference, including the DOI and a URL to the published version of the article on the journal website.

To assist our authors in disseminating their research to the broader community, our SharedIt initiative provides you with a unique shareable link that will allow anyone (with or without a subscription) to read the published article. Recipients of the link with a subscription will also be able to download and print the PDF.

As soon as your article is published, you will receive an automated email with your shareable link.

You can now use a single sign-on for all your accounts, view the status of all your manuscript submissions and reviews, access usage statistics for your published articles and download a record of your refereeing activity for the Nature journals.

An online order form for reprints of your paper is available at https://www.nature.com/reprints/author-reprints.html. Please let your coauthors and your institutions' public affairs office know that they are also welcome to order reprints by this method.

Sincerely,

Tiago

Tiago Faial, PhD
Senior Editor
Nature Genetics
https://orcid.org/0000-0003-0864-1200
